# Word-of-mouth generated influences of different prepared dishes via online consumer purchases: Preliminary text-based research findings from "Jingdong Mall" flagship shops

**DOI:** 10.1371/journal.pone.0297972

**Published:** 2024-03-08

**Authors:** Xiaotian Xu

**Affiliations:** Business Administration, Faculty of Business, City University of Macau, Macau, China; Wroclaw University of Environmental and Life Sciences: Uniwersytet Przyrodniczy we Wroclawiu, POLAND

## Abstract

With the rapid development of China’s prepared vegetable product market, consumer demand for prepared vegetable products is increasing. The study adopts a qualitative research method to construct a model of factors influencing the generation of consumer word-of-mouth (IWOM) in the online consumption context, taking the real consumer word-of-mouth of Jingdong’s own flagship shop of prepared dishes as the object of the study. The model states that the objective factors that promote the generation of word of mouth include specific consumption context (emotionality, initiality, scarcity, convenience and process) and specific product attributes (richness, safety, accessibility and emotionality). Meanwhile subjective factors design consumer satisfaction with the product (satisfaction with a single attribute and satisfaction with the brand as a whole) and emotions (positive self-conscious emotions, high arousal positive emotions and medium arousal positive emotions). Objective factors may contribute directly to the generation of word-of-mouth, or they may further contribute to word-of-mouth generation through subjective consumer factors. In addition, IWOM is often generated not by a single factor, but by a combination of factors.

## 1. Introduction

The market size of prepared dishes is booming in China with the accelerated pace of life and aging trend, becoming an integral part of people’s daily diet [1]; It not only simplifies the steps of daily food preparation and shortens cooking time, but also meets consumers’ demand for nutritious, healthy and functional food [2]. As a standardised pre-packaged dish, word-of-mouth (WOM) evaluation plays a crucial role in the prepared food market. Consumers’ word-of-mouth evaluations of prepared dishes can not only influence other consumers’ purchasing decisions, but also have a significant impact on the image and reputation of prepared dish brands. Therefore, it is important to study the influencing factors of consumers’ word-of-mouth generation of prepared dishes in the social network context to improve the awareness and reputation of prepared dish brands and to promote the development of the prepared dish market.

With the booming development of the Internet, it has become increasingly important for consumers to be keen on purchasing food products online, especially meat and meat-related products, which play a crucial role in the normal functioning of the nervous system and immune system [3]. In recent years, the rapid development of e-commerce platforms and the increasing improvement of cold chain logistics have led to an increasing number of consumers preferring to purchase meat products online. This shift has not only enriched consumers’ shopping choices, but also brought some new challenges. Consumers are particularly concerned about health and food safety issues, not only because of previous major food safety incidents, but also because they are skeptical about the safety standards of the meat industry. With the popularity of the Internet, Internet Word of Mouth (IWOM) is becoming a key factor influencing consumer purchasing behavior. In a U.S. study, IWOM and online communities played an important role in influencing consumer purchasing decisions, with consumers more inclined to trust and accept online reviews and recommendations from other consumers than traditional advertising media [4]. In Karachi, Pakistan, a city in South Asia, studies have shown that word-of-mouth has a positive impact on improving consumer purchasing decisions, and that word-of-mouth not only influences consumers’ willingness to buy, but also their evaluation of products or services [5]. Similarly, in China, online word-of-mouth has a significant impact on consumers when purchasing items, and consumers are more inclined to trust the reviews and recommendations of other purchasers.

In modern society, social networks have become an important platform for people to obtain information, disseminate information and exchange opinions. By posting comments, liking and sharing on social networks, Consumers can evaluate the dishes they have purchased by word-of-mouth (WOM) products and spread them to other consumers, thus influencing more people’s purchasing decisions. Therefore, brands should actively use social networks to manage word-of-mouth information to ensure accuracy, authenticity, and positive influence of word-of-mouth information. The rapid development of the prepared food market and consumers’ reliance on word-of-mouth (WOM) reviews make it particularly important to improve the awareness and reputation of prepared food brands and promote the development of the prepared food market by investigating the mechanism of consumers’ influence on the generation of word-of-mouth (WOM) about prepared food under social network conditions. Therefore, this study attempts to use a qualitative research method based on rootedness theory to comprehensively examine the factors affecting consumers’ word-of-mouth generation in social network situations by taking consumers’ real word-of-mouth in the purchase reviews of prepared dishes inside the Jingdong self-owned flagship shop as the research object, in order to supplement the literature related to word-of-mouth generation. Meanwhile, the study of consumers’ word-of-mouth evaluations of prepared dishes can help them better choose and purchase prepared dishes, improve consumers’ purchasing experience and satisfaction, and is of great significance to the development of the prepared vegetable market and the improvement of consumers’ living standards.

## 2. Brief literature review

Prepared dishes originated in the United States and emerged in Japan, Europe, the United States and Japan’s prepared dishes developed earlier, has formed a more perfect and mature system. Early research in foreign countries mainly focused on the impact of raw materials and their processing methods on the sensory quality of products [6,7], the difference between the vacuum low-temperature treatment process and the nutritional quality of traditional dishes [8,9]. With the passage of time, the research direction is gradually shifting to the establishment of the risk model for preventing bacterial contamination of prepared dishes [10], detection of MSG addition in food products [11], nutritional quality assessment of prepared dishes [12], Sterilisation technologies for the treatment of prepared dishes [13], the development of functional products, such as low-sodium seasonings [14] and the impact of industrialised prepared dishes and traditional dishes on public consumption habits [15]. In recent years, foreign research has focused more and more on improving the quality of prepared dishes. In recent years, foreign research has increasingly focused on improving safety control systems, regulating the nutritional value of prepared products, and using new technologies to improve product quality and safety [16–20].

Early domestic research on prepared dishes was mainly based on the industrial production of pre-mixed meat products and dishes [21]. Sun Baoguo [22] and Wang Jing [23] analysed the key scientific issues in the industrial production process of major Chinese traditional food products and dishes, and proposed a development direction for the modernisation of Chinese traditional food products. Zhang Yujie [24] and Li Manxiong [25] discussed the preservation technology of aquatic seasoned products, the processing of freshwater fish prepared dishes and the progress of technological research. In addition, Dequan Zhang [26] and Zhang Hong [27] also discussed the industrialisation technology of prepared dishes, the formation of the quality of prepared dishes and the development trend of the industry. There is a growing concern about the hygiene and safety of prepared dish enterprises in China, and Wang Jichuan [28] investigated and analysed the microbiological contamination when investigating the prepared dish enterprises. An Junwen [29] and Tang Jiaquan [30] conducted a study on the current situation and future development trend issues of the industry of prepared dishes industry, as well as Tsai [31] and Hu Hanzhong [32] considered that the catering industry chain for the processing and production of prepared dishes can be developed by adopting a central kitchen, etc. Meanwhile, Li Dongmei [33] believes that the whole industry of Chinese cuisine should be innovated. Other researchers also put forward their insights on local specialities, such as Zhang Yuhao’ [34] suggestions on the development of Sichuan-style prepared dishes, Xu Yujuan [35] on of Cantonese prepared dishes and Wang Jianhui [36] on of Hunan cuisine.

Prepared dishes, as a convenient and fast cooking method, have gained widespread attention in the restaurant market in recent years. The influence mechanism of its word-of-mouth is an important consideration for consumers to choose prepared dishes. In this context, the research results of Ali et al. and Lai provide this paper with an in-depth perspective to analyze the two major factors, objective and subjective, that influence the word-of-mouth of prepared dishes [37,38]. Objective factors: food taste: taste is the most direct and intuitive feeling consumers have about prepared dishes. A good taste can instantly grab consumers’ attention and increase their acceptance and satisfaction of prepared dishes. Food quality: Quality is the lifeline of food, especially in the prepared food market. High-quality prepared food means fresh ingredients, excellent craftsmanship and safe and hygienic production process. Price: Price is an important basis for consumers to judge the cost-effectiveness of prepared dishes. Reasonable pricing strategy can reflect the value of prepared dishes and attract consumers’ willingness to buy. Hygienic conditions: Hygienic conditions are directly related to the safety of prepared dishes. A good hygienic environment can make consumers trust the prepared dishes, and then promote the spread of word of mouth. Subjective factors: word-of-mouth recommendation: word-of-mouth recommendation is one of the important ways for consumers to get information about prepared dishes. The content of word-of-mouth recommendations, such as close friends and online reviews, can often influence consumers’ purchasing decisions. Satisfaction and Loyalty: Consumers’ satisfaction and loyalty to prepared dishes are important drivers of their word-of-mouth communication. When consumers are satisfied with a particular prepared dish, they are more likely to become loyal advocates and spread positive word of mouth about it.

Ladhari has suggested that emotions and satisfaction are key factors in shaping consumer word of mouth, where emotions are further acting on word of mouth by influencing satisfaction [39]. Based on this, this study further extends the influencing factor model to include important objective factors—consumption context and product attributes. Specifically, the consumption context was subdivided into five major categories: emotional, initial, scarcity, convenience and process. Among them, affective consumption situations mainly refer to gift-giving situations (e.g., harvesting gifts, giving gifts to others, and self-gifting) [40]; initial situations are when consumers try a product for the first time, having never had the same experience before [41]; and convenience situations refer to the situations in which consumers choose to buy or use a product that can quickly satisfy their needs, save time and energy, and achieve convenient and efficient life [42]; Scarcity context refers to include fire sales and limited time promotions [43]; process context refers to mainly include consumer expectations and fast delivery [44].

Product attributes, on the other hand, cover product richness, approachability, safety, and emotionality. Among them, product richness involves rich taste and nutrition; product safety involves health certification; product accessibility involves affordable price; and product emotionality represents celebrity endorsement [45,46]. In addition, this study delved into the multidimensional characteristics of emotions, which were refined into positive self-awareness emotions, high arousal positive emotions, and medium arousal positive emotions, in which high arousal positive emotions include surprise, satisfaction, etc.; medium arousal positive emotions include liking, happiness, etc.; and positive self-awareness emotions include happiness [47,48].

**Fig 1:**
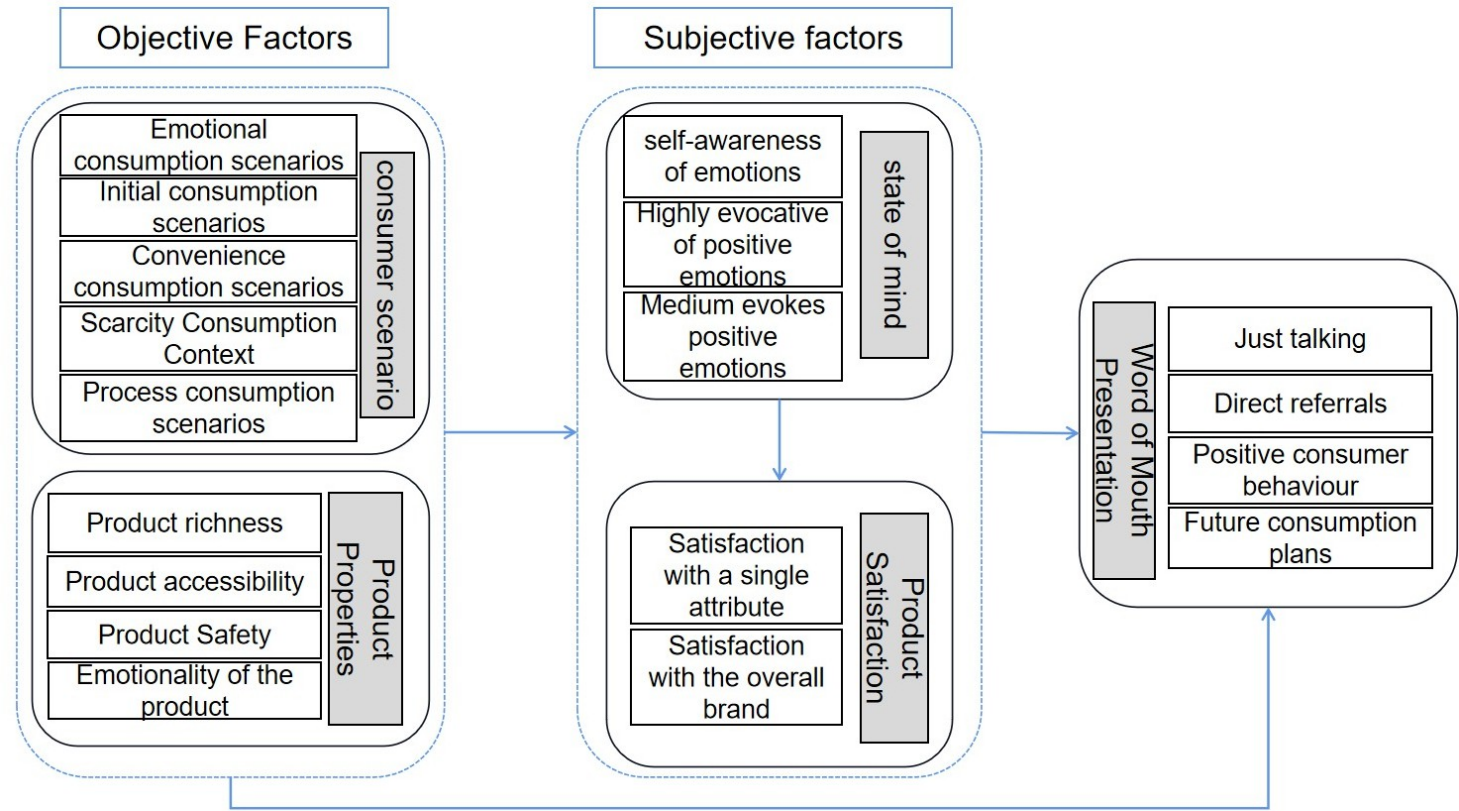
Mechanisms influencing the generation of word-of-mouth about online consumer purchases of prepared dishes.

The presentation of word-of-mouth includes just talking about it, direct recommendation, positive consumption behavior and future consumption plans. Berger [49] proposed three aspects of word-of-mouth, namely "direct recommendation", "picture presentation" and " mere talk". Scholars such as Li Yan extended two aspects based on this, including "positive consumption behavior" and "future consumption plan" [50]. "Positive consumption behavior" means that the content of word-of-mouth includes consumers’ positive experience of using the product or service. "Future consumption plan" means that the IWOM content involves a description of one’s future consumption behavior in terms of one’s willingness to repeat the purchase or consumption of the product. The interactions among the factors in the model are shown in(Fig 1), and will be explored one by one in the subsequent model elaboration.

Domestic and international studies on IWOM generation can be classified into positive, neutral and negative IWOM based on efficacy. However, many studies about consumer IWOM do not subdivide positive IWOM and neutral IWOM [49,51]. Similarly, consumer IWOM referred to in this study includes both positive and neutral IWOM. Previous research on IWOM generation has focused on the psychological motivation of consumers to post IWOM [52–55], social contexts that influence IWOM generation [56,57] and product characteristics [57,58] etc. While the psychological motivation of consumers to post IWOM, the influence of social contexts, and product characteristics have been relatively well researched in the past, the factors influencing consumer IWOM generation have not yet been fully explored in the field of prepared dishes.

## 3. Research methodology

### 3.1 Data collection of word-of-mouth texts

Given that this channel has the characteristics of real consumer identity, immediacy, openness, etc., the researcher is able to observe a more free and real speech, which meets the basic requirements of being rooted in theory and reality, and systematically collecting and analysing data [59]. The reason for choosing the "Jingdong Mall prepared dishes Flagship Store Reviews" is that the platform is rich in product reviews, and consumers usually mention the quality and taste of the products when evaluating them, and there are also real consumer identities and a high degree of information richness. The study selected four Wangjiadu, Qianwei Yangchu, Anjing, and Guolian prepared dishes self-owned flagship shops on Jingdong Mall, which all belong to the top ten prepared dish brands in the list of top ten prepared dish brands in 2023. Each of these items was selected according to the degree of processing of the prepared dishes, and can be divided into four main categories: ready-to-eat food: Wangjiadu (luncheon meat, grilled sausage), ready-to-heat food: Qianwei Yangchu (fancy no-cut cake, dumpling), ready-to-cook food: Anjing (hot pot meatballs, pickled fish), and ready-to-complete food: Guolian (Shrimp, Basa catfish) (See Table 1). Two products from one company were selected as representatives for each category. In this study, a total of 8 keywords were set to be searched, and 110 pieces of matching IWOM information were searched based on each keyword, and a total of 880 pieces were collected, of which 800 pieces of IWOM information were used as model construction, while the remaining 80 pieces of IWOM information (10 pieces of each keyword were set aside) were used as tests of the theoretical saturation of the model.

**Table 1:** Keyword settings for word-of-mouth text collection.

Types of prepared dishes	Product Explanation	Representative companies
instant food	Refers to prepared food products that can be consumed directly after opening and can be summarised as "ready-to-eat" food.	Wang Jiadu (Luncheon meat A, Grilled sausage B)
Heated food	Refers to foods that are ready to eat after only being heated, and can be summarised as "heat-and-eat" foods.	Qianwei Yangchu (fancy no-cut cake C, Dumpling D)
ready-to-cook food	Refers to semi-finished ingredients that have been relatively deeply processed (heated or shallow fried) and portioned into portions to be stored refrigerated or at room temperature, ready to be served immediately in the pan. Foods that are seasoned with the addition of condiments are in the category of semi-finished prepared dishes.	Anjing (Hot pot meatballs E, Pickled Fish F)
ready-to-eat (RTE) food	It refers to small pieces of meat and raw vegetables that have undergone preliminary processing (physical processing only) such as washing and cutting. Generally, raw materials are the main ingredients, and the cook has to mix various seasonings on his own, and after stir-frying and heating, they become cooked and can be eaten, which can be summarised as "raw heated and seasoned" food.	Guolian (Shrimp G, Basa catfish H)

The word-of-mouth text information used in the study is different from normal review text information and is extremely intrusive when searching; many consumer reviews will mention the product, but not the word-of-mouth we need. This time it is necessary for the authors to understand the real meaning of word-of-mouth as well as to determine whether the usage is correct or not in order to identify the real word-of-mouth. The study established the following requirements when selecting the sample IWOM texts: first, they must be positive or neutral IWOM, not negative IWOM. Second, when searching for texts, it is necessary to exclude abnormal texts or sales information such as "swipe", "duplicate reviews", "reviews of other products", etc., and select only the real reviews of consumers (or endogenous IWOM). information (or endogenous word-of-mouth) rather than business incentivised word-of-mouth. Third, the publisher of the product should be the person who published it, not others, and the same publisher should not be selected to review the product multiple times. Fourth, the text of the IWOM should be about the product or brand, not about the product or brand in passing.

### 3.2 Rooted theoretical approach

Rooted theory is a method in qualitative research whose main purpose is to build a theory from empirical data. The researcher generally has no theoretical assumptions before the study begins, and starts directly from actual observation, drawing empirical generalisations from the primary data and then moving up to theory. This is a method of building substantive theories from the bottom up, i.e., searching for core concepts reflecting social phenomena on the basis of systematically collected information, and constructing relevant social theories through the connections between these concepts. The general process of the rooted theory approach involves open coding, principal axis coding and selective coding of qualitative data to construct a theoretical model.

#### 3.2.1 Open coding

In accordance with open coding requirements, the study coded the 800 selected IWOM texts. The formation of additional concepts and categories was facilitated through progressive conceptualisation and categorisation of qualitative information and continuous comparison. Each message was analysed with respect to the context, IWOM target characteristics, consumer satisfaction, consumer sentiment, and IWOM presentation patterns. In order to save space, a number of categories and initial concepts were abstracted from the textual information after labelling and attaching and repeatedly collating and analysing each IWOM message.

#### 3.2.2 Spindle coding

Principal axis coding involves analysing segmented information in open coding with class clustering to establish associations between different categories. Principal axis coding aims to better develop and refine the main categories by further exploring and enriching the nature and dimensions of the individual categories to make them more precise and closely linked. At the same time, it also endeavours to link the separate categories and to reveal and construct the underlying logical relationships between these categories [60]. In this study, by applying the spindle analysis method, a particular pattern of categorisation or correlation between antecedent factors in the process of generating consumer word-of-mouth has been identified (See Table 2).

**Table 2:** Main categories of spindle code formation.

form	main category	Corresponding category	Connotation of scope
consumer scenario	Emotional consumption scenarios	gift-giving situation	Consumers typically have positive emotional experiences in scenarios where they offer a gift, receive a gift from someone else, or buy a gift for themselves
	Initial consumption scenarios	first attempt	Consumers try a product for the first time when they have never had a similar experience before
	Convenience consumption scenarios	Convenient and time-saving	Consumers are choosing to buy or use products that meet their needs quickly, save time and effort, and achieve convenient and efficient living situations
	Scarcity Consumption Context	fire sale	A situation where consumers show high interest and demand for a product, making it a fast seller in the marketplace
		time-limited promotion	Situations based on buying opportunities where supply is at a certain level and demand is increasing
	Process consumption scenarios	Consumer Expectations	Consumers usually engage in a relatively long process of anticipation before making a purchase or consuming, a process that is characterised by a certain amount of uncertainty but often also accompanied by a positive emotional experience
		Rapid delivery	Consumers can obtain delivery of goods within a short period of time after purchasing the product, and although there is a time lag between purchase and acquisition, there is greater certainty of acquisition
Product Properties	Product richness	rich in flavour	A wide range of ingredients in the product to create a rich and varied taste experience and to stimulate the consumer’s desire to buy.
		nutritious	The product contains a lot of nutrients, the human body’s daily nutritional needs to provide assistance, to attract consumers to come to buy
	Product accessibility	affordable	The product has the characteristics of common, ordinary, etc., the price is cheap, the necessity of life.
	Product Safety	health certification	There are no additives and some harmful ingredients in the product, making the ingredient list cleaner.
	Emotionality of the product	celebrity endorsement	The association of products with stars and celebrities creates opportunities for mutual communication and interaction with others, and increases the ways in which they can be emotionally experienced.
state of mind	self-awareness of emotions	bliss	Emotions felt by consumers when they feel positive and good emotions in their self-awareness
	Highly evocative of positive emotions	astonished	Positive emotions felt by consumers when they feel that something surprising and unexpected has happened, with high levels of emotional arousal
		meet (the needs of)	Positive emotions of consumer satisfaction as a result of purchasing and acquiring a product, with a high level of emotional arousal
	Medium evokes positive emotions	Like, happy.	Consumer’s positive emotions of liking or being happy as a result of buying or getting a product
Product Satisfaction	Satisfaction with a single attribute	Recognition of efficacy	Satisfied with the efficacy of the product, the efficacy experience exceeded the expected results
		Recognition of the environment	Satisfied with the environment in which the product was produced and transported, and the environmental experience exceeded expectations
		Approved Appearance	Satisfied with the appearance or form of the product, the design exceeds expectations
		Recognising value for money	The actual value for money of the product exceeded expectations, as there was a high level of satisfaction with the price or quality of the product at the same level of price or quality
	Satisfaction with the overall brand	Brand satisfaction	Satisfied with the brand name as a whole and the actual experience exceeded expectations
Positive word of mouth	The way word of mouth is presented	Just talking	Discusses only the product or merchant itself and does not obviously indicate a recommendation
		Direct referrals	Actively recommending products or businesses in order to promote purchase or consumption by others, attempting to persuade others
		Positive consumer behaviour	Description of the product they have fully utilised or made multiple purchases, description of their previous consumption behaviour
		Future consumption plans	One’s own desire to repeat or consume the product, a description of one’s future consumption behaviour

#### 3.2.3 Selective coding

Based on principal axis coding, selective coding further systematically deals with category-to-category connections. It does this by mining the core categories within the main categories and analysing the connections between the core categories and the main and other related categories, as shown in Table 3. This study constructs and develops a theoretical framework of the factors influencing the generation of consumer word-of-mouth (IWOM) based on a typical relational structure, as shown in Fig 1. There are both objective and subjective factors that motivate consumers to release word-of-mouth, and both of them may directly contribute to word-of-mouth generation; at the same time, objective factors may also have an impact on word-of-mouth through subjective factors. In many cases, it is not a single objective or subjective factor that explains IWOM generation, but rather multiple interacting elements that contribute to the process.

**Table 3:** Typical relational structure of main categories.

Typical relationship structure	Connotation of the structure of the relationship
Consumption context → word-of-mouth behaviour	Specific consumption contexts such as emotionality, initiality, convenience, scarcity, and process are objective contextual factors that promote consumer word-of-mouth generation
Product attributes → word-of-mouth behaviour	Attributes such as product richness, accessibility, safety and emotionality are objective product factors that promote consumer word-of-mouth generation
Consumer sentiment → word-of-mouth behaviour	Some highly and moderately aroused positive emotions as well as positive self-conscious emotions are subjective emotional factors that help to facilitate the generation of consumer word-of-mouth.
Satisfaction with the product → word-of-mouth behaviour	Consumer satisfaction with the brand as a whole or with a particular attribute of a product is a subjective cognitive factor that helps to promote the generation of consumer word-of-mouth
Consumption context → subjective factors → word-of-mouth behaviour	Specific consumption situations may contribute to consumer word-of-mouth by influencing subjective factors (emotions and product satisfaction).
Product attributes → subjective factors → word-of-mouth behaviour	Specific product attributes may contribute to consumer word-of-mouth through their influence on subjective factors (emotions and product satisfaction).
Emotions → satisfaction with the product → word-of-mouth behaviour	Emotional evaluations of products have a significant impact on the formation of consumer word-of-mouth, as consumer emotions may contribute to word-of-mouth by influencing satisfaction with the product.

#### 3.2.4 Theoretical saturation test

The theoretical saturation test is one of the criteria for the end of sampling, meaning that no new data can be acquired to enrich the analyst’s further knowledge of a feature category [61]. To test for theoretical saturation, the remaining 80 IWT texts were coded and analysed in the same way, with five IWTs listed below as examples:

Text 1: C45 "The osmanthus cake was a gift, I had thought that the gift was not very good, but when I ate it, I found that the business is still quite conscientious, the osmanthus cake is very tasty, sticky and soft, and it tastes faintly sweet, but it’s just right for people like me who don’t love sweets!" This word-of-mouth generation process conforms to the path of "emotion (high arousal positive emotion: surprise)—word-of-mouth" in Fig 1.

Text 2: F5 "This brand is needless to say, it is for this brand to come, at least rest assured. It’s much more affordable than the supermarket! The taste is also very awesome, like me these kitchen little white can also easily manage." The word-of-mouth generation process conforms to the "product satisfaction—word-of-mouth" path in Fig 1.

Text 3: A34 "Small packages are especially suitable for a family of three a breakfast, the meat is delicate and full, delicious, bread and milk is an excellent partner, enough to provide the body with the required protein and nutrition, will always buy back" The IWOM generation process is in line with the pathway of "product attributes (product richness: nutritious)—word of mouth" in Fig 1. The process of word-of-mouth generation is in line with the path of "product attributes (product richness: nutritious)—word-of-mouth" in Fig 1.

Text 4: B38 "Brand products, trustworthy, well packaged, easy to take, inside the vacuum packaging, eating is also convenient, clean and hygienic. The taste is delicious, adults and children like to eat, frying and cooking, are very convenient." The IWOM generation process is in line with the path of "Consumption Context (Convenience Consumption: Convenience, Time Saving)-Word of Mouth" in(Fig 1).

Text 5: G20 "The shrimp is exceptionally large, clean and no ice, the texture of the teeth, not the kind of powdered noodles, after purchasing countless times, as always, good, Guolian’s emerald shrimp children, although a little expensive, but the quality is really good, catching up with promotions on the stockpile to buy some more, vacuum divided into a few small packages for storage, freshness and save the land." The IWOM generation process conforms to the path of "consumption context (scarcity consumption context)—product satisfaction—IWOM" in Fig 1. According to the results, the remaining word-of-mouth text is basically consistent with the path of the model in Fig 1, and no new categories or relationships are generated. According to the viewpoint of rootedness theory, if the new cases cannot shake the previous themes or hypotheses, then the research is sufficient [62]. Therefore, it can be concluded that the model in Fig 1 is saturated.

## 4. Discussion of research findings

### 4.1 Objective factors: Consumption contexts contribute to word of mouth generation

Specific consumption situations are objective factors that trigger consumers to generate word-of-mouth, and not all consumption situations trigger word-of-mouth. According to this study, emotional, initial, scarcity, convenience and process consumption situations are prone to trigger word-of-mouth.

#### 4.1.1 Affective consumption situations

It mainly refers to gift-giving situations (e.g., gift harvesting, gift-giving, and self-gifting). Gifts represent an important symbolic gesture in Chinese social relationships, especially when they occur in the context of power differentials and relational closeness in social relationships [40]. One of the purposes of gift-giving is to strengthen the emotional connection between the giver and the recipient of the gift. At the same time, social connection is an intrinsic motivation for consumers to share word-of-mouth [49]. Therefore, the motivation to strengthen social ties not only promotes individual gift-giving behaviour, but also the generation of word-of-mouth sharing. Meanwhile, word-of-mouth feedback and interactions from gift recipients also promote word-of-mouth formation. In addition, self-gifting is a symbolic means of individual self-communication, and individuals can engage in self-gifting by rewarding themselves [63]. Sharing gift-giving-related word-of-mouth in the after-sale of the Jingdong self-owned flagship shop is also a way for consumers to gain mental self-rewards by displaying their selves.

#### 4.1.2 Initial consumption situations

Primarily refers to first attempts. First try is when a consumer tries a product for the first time, having never had the same experience before. During the initial purchase stage, consumers are usually curious about the product. Since people’s attention span is limited, they tend to pay more attention to new and innovative products. Therefore, consumers are often curious about new or innovative products. Consumers’ curiosity is a positive consumption attitude that can motivate them to make positive evaluations of products [41]. Therefore, curiosity increases consumer satisfaction with a product during the initial trying stage in an initial consumption situation, which promotes positive word-of-mouth.

#### 4.1.3 Convenience consumption scenarios

It mainly refers to convenience and time-saving. Convenience and time-saving means that consumers choose to buy or use products that can quickly meet their needs and save time and energy to achieve a convenient and efficient life situation. When consumers buy food in the choice and purchase of food, consumers are more inclined to choose the food that is convenient and time-saving to do, these foods usually bring a certain degree of pleasure and satisfaction, but also meet the need to save time and energy at the moment, which can allow consumers to enjoy life more efficiently [42]. Therefore, consumers in a convenience consumption situation will be more favourable to foods that they can save time, thus promoting the formation of good word of mouth.

#### 4.1.4 Scarcity consumption situations

This mainly includes fire sales and limited time promotions. A fire sale is a situation in which consumers show a high level of interest and demand for a product, resulting in a rapid sale of the product in the marketplace. Time-limited promotions are situations where there is an opportunity to buy based on supply at a certain level and increasing demand. The reality of many consumer situations in the purchase opportunity will be gradually reduced with the commodity fire, such as commodities recently in doing holiday activities will be discount promotions, at this time the commodity will limit the sales volume, limit the sales time. At this time the goods will be subject to consumer impulsive consumption under the purchase of scarce goods, to achieve to meet the consumer’s tasting needs, thus promoting the formation of a good word of mouth [43]. Thus, fire sales and limited time promotions are classified as scarcity consumption situations.

#### 4.1.5 Process consumption scenarios

This mainly includes consumer expectations and fast delivery. It was found that positive consumption expectations change consumers’ actual perception of food quality [44]. As shown in the article, positive consumer expectations increase satisfaction with the product and also enhance willingness to share by word of mouth. Especially in online shopping, the short interval between the purchase time and the arrival time of the goods without a long waiting time can also have a positive signalling effect on the product due to the very fast delivery speed.

### 4.2 Product factors: Product attributes inspire consumer word-of-mouth generation

Specific product attributes are objective factors that trigger consumer word of mouth. This study found that product richness, safety, accessibility, and emotionality are likely to trigger word-of-mouth generation. Richness involves rich taste and nutrition, safety involves health certification, accessibility involves affordable price, and emotionality represents celebrity endorsement. Previous studies have shown that when we care about a product, the social currency value of that product goes up and we share and spread it [45]. Affordability increases how much people care about a product, which promotes word-of-mouth generation. Safety or abundance will also cause word of mouth, possibly because people will care more about how well the food is produced and how nutritious it is. Previous studies have found that people are more likely to share symbolic products than functional products when it comes to word-of-mouth communication, and that the identity signalling effect of symbolic products can be more pronounced, so sharing these products with symbolic attributes can better create a good image for consumers [46].

Celebrity endorsement is categorised as the emotional nature of product attributes. Celebrity endorsement refers to the ease of triggering word-of-mouth generation when a product is clearly associated with a particular person. Examples include the endorsement of track and field athlete Su Bingtian and the endorsement of skater Wu Dajing. Past research has found that when the person issuing the word-of-mouth and the person receiving the word-of-mouth share a common cognitive base, then it can lead to an increased emotional connection between the two parties, making the communication between the messages more effective [49]. For example, celebrities are often used as spokespersons in emotional advertising, and people draw on their positive emotions towards the celebrity, which are then transferred inside the product.

### 4.3 Subjective factors: The influence of specific emotions and product satisfaction

#### 4.3.1 Influence of specific emotions on consumer word of mouth generation

Specific emotions are subjective factors that trigger word-of-mouth among consumers. These emotions can be described in two ways: potency and arousal. Potency reflects how pleasurable the emotion is, while arousal indicates the level of physiological activation that the emotion brings about [47]. Emotions can be classified into two types: basic emotions and self-conscious emotions. Specific emotions in the model include high or medium arousal positive emotions and positive self-conscious emotions. According to the dimensional theory of emotions, high arousal positive emotions include surprise, satisfaction, etc., medium arousal positive emotions include liking, happiness, etc., and positive self-conscious emotions include happiness [48]. Westbrook points out that highly arousing emotions motivate consumers to spread word of mouth, and that the influence of emotions on word of mouth does not require a cognitive premise based on satisfaction [64]. According to the Extended and Constructed Theory of Positive Emotions, positive emotions help to broaden attention, making it easier for people to perceive opportunities in their environment and to choose behaviours in a flexible way to maximise the benefits of these opportunities [65]. Pleasant experiences can broaden the scope of what an individual expects to do at the moment, which also includes the act of spreading word of mouth.

In terms of the level of emotional arousal, consistent with previous research, this study also found that emotional arousal has a significant impact on word-of-mouth generation. For example, Berger et al. showed that news stories that were highly emotionally arousing were more likely to be forwarded via email [52]. This means that when individuals experience a certain level of psychological arousal, they tend to express and disseminate more opinions, insights, or experiences. Thus, in the process of word-of-mouth generation, even if it stems from behaviours or events that are not emotionally related, the possibility of triggering word-of-mouth effects through active participation and sharing still exists. Typically, low levels of arousal are difficult to trigger a word-of-mouth effect among consumers [39]. Passion and reason in Passion and Reason refer to self-awareness emotions as encompassing happiness, and self-awareness emotions differ from other basic emotions in that their emergence is accompanied by complex perceptions about the self [66]. Happiness is an important emotional state that plays an active role in driving word-of-mouth generation. When individuals experience positive outcomes such as delightfulness, fulfilment, or success, they develop a deep and beautiful emotion—namely happiness. And this happiness can lead to individuals being more inclined to share their opinions, insights, and positive experiences. By conveying positive messages of pleasure, achievement or satisfaction associated with a product and translating them into an authentic and engaging narrative (e.g., brand storytelling), you can inspire a sense of inner happiness in your customers. This positive self-awareness will further drive word-of-mouth generation, making consumers more willing to share their positive experiences and opinions, which will lead to more attention, recognition and word-of-mouth for the brand or product [67].

Specific emotions can contribute directly or indirectly to IWOM generation, and their generation may be influenced by objective or other subjective factors. This study suggests that emotions play an important role in triggering IWOM generation, and that the IWOM effect usually occurs after specific emotions are aroused. For example, "first try-satisfaction-word of mouth", "consumer expectation-surprise-word of mouth", "product health certification-surprise-word-of-mouth" and other processes can be seen in this association.

#### 4.3.2 Influence of consumer satisfaction with products on word of mouth generation

Consumer approval of products responds to higher levels of consumer satisfaction. Word of mouth is an outcome of consumer satisfaction and there is a strong link between high levels of customer satisfaction and positive word of mouth communication. When consumers are highly satisfied and have received a good service experience, they are more likely to share their positive evaluations and recommend them to other potential customers. Also when consumers are satisfied with a product or service, they usually associate this satisfaction with the brand and perceive the brand as trustworthy [68]. Previous research has explored the reasons for the positive relationship between consumer satisfaction and word-of-mouth, Altruistic motivation: consumers are motivated by a desire to help others by sharing their own positive buying experiences and product reviews to recommend to other potential customers. This altruism motivates consumers to participate in word-of-mouth and to see it as beneficial or supportive of the social group; instrumental motivation: some consumers tend to use word-of-mouth as a tool to show that they are knowledgeable, smart and have expertise. By sharing what they know and becoming a ’connoisseur’, they increase their social status and gain a sense of recognition; Self-defence: when individuals suffer dissatisfaction, negative experiences or disappointment, they may choose to express this and warn other potential customers to avoid similar situations. Doing so can be seen as a way of protecting one’s rights and interests [69]. In this study, we were only able to observe high levels of consumer satisfaction with the product and were unable to observe the motivational factors that influenced them to post word-of-mouth. In fact, high consumer satisfaction with the product is largely a necessary precondition for other influences to work.

### 4.4 Relationship analysis of factors influencing consumer word of mouth generation

#### 4.4.1 Consumer word of mouth presentation driven by different factors

Word of mouth is presented in terms of talk only, direct recommendations, positive consumption behaviour and future consumption plans [49]. Berger proposed three aspects of IWOM, i.e., "direct recommendation", "picture presentation" and "just talking about it". Li and other scholars expanded on this by adding two more dimensions, including "positive consumption behaviour" and "future consumption plans" [50]. The present study builds on the work of Li and others. This study improves on the framework of Li and other scholars and makes adjustments to its content. Specifically, we exclude the factor of "picture display" in this study. This is due to the fact that when we chose to select reviews from the self-owned flagship shop of Jingdong Mall, we found that the number of reviews with pictures available for reference was relatively small. In order to ensure that the results of this study are true and reliable, we decided not to adopt "picture display" as one of the ways of presenting word of mouth. At the same time, the study redefines and explains the concepts of "active consumption behaviour" and "future consumption plan" as mentioned by Li Yan and other scholars to fit the current target audience and objectives.

"Positive consumer behaviour" refers to word-of-mouth content that includes positive experiences with a product or service. It is also a description of one’s previous consumption behaviour on behalf of having made full use of the product, or having made several large purchases. Positive consumption behaviour refers to a behaviour taken by a consumer after purchasing a product, finding the target product very tasty and evaluating it positively. For example, products are purchased in large quantities many times and authentic positive evaluations are a concrete demonstration of positive consumption behaviour. The study concludes that consumers’ descriptions of positive consumption behaviour play an important role in word-of-mouth presentation, thereby positively influencing word-of-mouth recipients.

"Future consumption plans" refers to word-of-mouth content that involves one’s intention to repeat or consume the product and describes one’s future consumption behaviour. This behavioural plan has a socially sanctioned effect and has a reference group effect on other consumers [50]. People often get guidance on how to act by observing those around them, and information from reference group members about product choices can influence them to form brand preferences. Word-of-mouth audiences are often more likely to be positively influenced when a word-of-mouth publisher describes plans for future consumption than when the product itself is simply mentioned.

#### 4.4.2 Logical relationships between different factors

The model shown in the study involves both objective and subjective factors in the generation of consumer word-of-mouth, and there is a logical relationship between them in the following ways:

First, objective factors can be divided into two categories: contextual factors and product attributes. These two types of objective factors are independent of each other, and they can either affect IWOM generation individually or have a superimposed positive effect on IWOM generation at the same time.

Secondly, subjective factors can be categorised according to product satisfaction and emotions. In terms of product satisfaction, when consumers are extremely satisfied with a particular product attribute or the overall brand gives them satisfaction, it helps to promote word-of-mouth formation, while in terms of emotion, word-of-mouth behaviour is more likely to be triggered after experiencing positive emotions as well as positive self-awareness. This again validates that expressing personal emotions is one of the most important motives that drive word-of-mouth generation.

Third, positive emotions may trigger more word-of-mouth behaviour by increasing consumer satisfaction. Prior research has demonstrated that certain positive emotions (e.g., happy, hopeful) have a positive effect on promoting consumers’ willingness to generate word-of-mouth, and that this effect is realised through increased consumer satisfaction. Furthermore, other studies have shown that positive emotions can have an impact on consumer satisfaction in service scenarios through a dual mediating effect—perceived benefits and perceived risks [70]. Therefore, the role that emotions potentially play in shaping product satisfaction is particularly emphasised in the model constructed in this paper.

Fourth, specific consumption situations may directly contribute to IWOM generation, however, it is more common to indirectly contribute to IWOM formation by influencing consumers’ emotions or satisfaction. For example, when consumers try a product for the first time, they are often surprised and happy, and this stimulates word-of-mouth behaviour.

Fifth, specific product attributes have the potential to directly stimulate word-of-mouth (IWOM) formation and at the same time indirectly contribute to IWOM generation by influencing subjective consumer factors such as emotions and satisfaction with the product. Health-certified products, for example, have the advantage of increasing consumer satisfaction, thus effectively contributing to word-of-mouth generation.

### 4.5 Characteristics of word-of-mouth generation in the context of online consumer purchases of prepared dishes

In order to fully understand the link between word-of-mouth generation and consumers’ overall perceptions of prepared dishes among consumers’ purchases of prepared dishes in the self-owned flagship shop of Jingdong Mall, a focus group interview session was introduced in this study. A total of 30 undergraduate and graduate students were recruited and divided into five groups for focus group discussions. Each group lasted approximately one hour and was guided by a moderator. The discussions centred around the question "What are the differences in word-of-mouth (IWOM) about buying prepared food at the Jingdong Mall flagship shop compared to other contexts? The facilitator was responsible for introducing the concept of IWOM generation, limiting the scope, asking questions, and controlling the core issues. Based on the transcripts, we summarised 200 valid focus group textual data, and based on these textual data, we derived opinions about the characteristics of IWOM generation in the context of purchasing prepared food at the Jingdong Mall’s flagship shop.

Firstly, about the characteristics of the content of the word-of-mouth on prepared dishes: in my observations, I found the following aspects. The first is information richness and diversity. Consumers provided detailed and comprehensive information when sharing their product experience and quality evaluation of prepared dishes, and demonstrated their use of the product and expressed their feelings through text descriptions, pictures or videos. Secondly, there is a strong tendency to be emotional. Consumers tend to closely tie their personal emotions to their eating experience, expressing different emotional responses such as favourite, satisfaction or disappointment in their reviews, and passing on these emotions to other potential buyers.

Second, about the word-of-mouth generation process: usually, after purchasing and trying a particular prepared dish product on the Jingdong Mall’s own flagship shop platform, users have the opportunity to leave evaluations and comments against the relevant pages of the product. These evaluations can include aspects such as how the food the user receives tastes, whether it looks appealing, and whether the delivery service is punctual and reliable. In addition, on social media platforms, users can generate word-of-mouth by sharing their purchase experience, cooking process, and eating experience. This information spreads quickly across social networks and triggers interest and discussion about the product among other users.

Finally, the possible impact after IWOM generation: the word-of-mouth generation of prepared dishes is of great significance to consumers. Firstly, it can provide a reference point and decision support for potential buyers. When they read about other people positively evaluating a particular prepared dish product, they are likely to be attracted to it and increase their motivation to try the item. In addition, the large number of positive or negative reviews formed in the context of Jingdong Mall’s own flagship shop will also directly affect the brand’s reputation as well as sales performance. If the majority of consumers are highly complimentary, this will help to build a good brand image and promote more people to choose to buy; conversely, a high number of dissatisfied or negative reviews may lead to customer attrition and the need to take steps to ameliorate the problem and restore trust.

In the Jingdong Mall self-owned flagship shop context, the content of the word-of-mouth information formed for the prepared dish products is more concise and clear; at the same time, it also presents more diversified characteristics in terms of form, including semantics, pictures and videos. This is because in the Jingdong self-owned flagship shop, consumers can only comment on the product without interacting with others, thus realising the combination of unidirectionality and interactivity in information dissemination. Compared to offline IWOM contexts, sharing IWOM of prepared dishes in the Jingdong Mall flagship shop has the following advantages: firstly, it has a wider audience and spreads quickly; secondly, it can help users quickly find resonance and promote social interaction to generate IWOM. In addition, in many cases, consumers are driven by impulsive emotions to generate word-of-mouth evaluations of products, and the finding is consistent with our research model.

### 4.6 Theoretical contributions and directions for future development

The theoretical contributions of this study are mainly in the following aspects: firstly, most of the past literature has discussed the causes of consumer IWOM generation in a fragmented manner, while existing articles have verified some of the factors influencing IWOM generation mainly from a quantitative perspective. However, research through the use of quantitative methods tends to cover only a small number of variables and potential influences, and lacks a comprehensive compendium of the logical relationships between these variables. To fill this gap, this study uses a qualitative research method combining rooted theory and group interviews for analysis. Through this approach, the various factors that influence online consumers in their purchases of prepared dishes are integrated into a theoretical framework (see Fig 1) and fill in the gaps that existed in previous quantitative studies.

Second, Regarding the contextual factors of IWOM generation, previous studies have mainly focused on the influence of social context and scarcity context on IWOM generation. However, this study effectively explores specific consumption contexts that have not been mentioned in the previous literature, such as the convenience consumption environment and the limited-time promotion link. In addition, this study adds product attributes such as richness, accessibility, and safety to the existing literature on IWOM generation.

Third, While only a small number of past studies have examined the effect of emotion type on IWOM, the present study adds to this by examining the role of specific positive emotions (e.g., happiness, contentment, and gladness) in inducing IWOM generation. This finding theoretically refines our knowledge of the emotional mechanisms involved in IWOM generation.

Fourth, the existing literature is lacking in exploring the characteristics of word-of-mouth generation in online prepared dish purchase contexts. To fill this gap, this study used focus group interviews to initially explore the characteristics of the content, process, and subsequent impact of word-of-mouth generation in online prepared food purchase contexts. These features make word-of-mouth generation in online prepared food purchase contexts significantly different from that in other formats.

The main shortcoming of the study is that it fails to include consumers’ motivations for posting word-of-mouth in the modelling considerations. This is because it is difficult to effectively identify the hidden motivation behind posting in IWOM texts, and consumers usually do not directly disclose their intrinsic motivation for sharing IWOM. Although we have explored the relationship between consumer satisfaction and word-of-mouth in depth, there is a lack of detail on the motivational factors behind posting behaviour. However, complementary perspectives have been provided in previous quantitative studies, and the theoretical model and analytical framework can be further optimised by drawing on findings from the literature on the motivational aspects of word-of-mouth (IWOM). By combining the results of previous research in the areas of individual or group reasons for engaging in communication activities, social influence, and psychological needs, we can more fully explain and predict the complex psychological processes involved in consumer engagement and the promotion of a positive brand image.

There is still much to consider for future research, especially as there are many fascinating research questions that deserve to be further explored in future studies. For example, why do consumers like to talk about products associated with celebrities? Alternatively, we could examine what factors influence the way word-of-mouth is communicated and the form in which it is presented. Although this study has been fruitful in some aspects by using rootedness theory as a qualitative research method, it still has limitations in terms of credibility and validity. To make the conclusions drawn from this study more convincing, follow-up empirical quantitative research is needed to validate and deepen the understanding of the relationships between the various perspectives, models and findings. By implementing quantitative surveys, experimental designs, or other means of empirical data collection, and by using statistical analysis tools for data processing and interpretation, we will be able to more accurately assess the relationships between different variables and provide more precise and comprehensive conclusions. Such follow-up quantitative confirmatory experiments will help consolidate the current qualitative results and enrich the field’s understanding of the motivations, behavioural characteristics, and influencing factors of IWOM. By combining qualitative and quantitative research methods, we can gain a more comprehensive understanding of the motivations, behavioural patterns, and influences behind consumer engagement with IWOM. This will help companies develop more precise and effective marketing strategies and provide practical suggestions to promote positive word-of-mouth generation and maximise its benefits. In addition, since this study only examines the online shopping of "Jingdong Self-managed Flagship Store", other types of online shopping may affect IWOM generation due to the differences in the attributes of the platform channels. Factors such as anonymity and psychological distance of online consumption may have a significant impact on IWOM generation.

## 5. Concluding remarks

The study provides a comprehensive and systematic examination of the factors influencing the generation of word-of-mouth about the purchase of prepared dishes by online consumers. The main findings are as follows:

First, specific consumption contexts (emotional, initial, convenience, scarcity, and process) may promote IWOM generation; specific product attributes (richness, accessibility, safety, and emotionality) may promote IWOM generation; specific emotions (high or medium arousal positive emotions, positive self-conscious emotions) may promote IWOM generation; consumer satisfaction with the product may promote IWOM generation; objective factors (specific consumption situations, specific product attributes) may contribute to IWOM by influencing subjective factors (emotions, product satisfaction); specific emotions may contribute to IWOM by influencing consumer satisfaction with the product.

Second, specific contexts can promote consumers to generate word-of-mouth. Taking the gift-giving scenario as an example, businesses encourage consumers to voluntarily purchase and give the product to their friends by providing appropriate themed packaging or promotional activities. Meanwhile, consumers are more likely to be motivated to post word-of-mouth information when aspects such as consumer expectations and fast delivery are met, which suggests that a prepared dish business that exceeds customer expectations will prompt customers to spread positive word-of-mouth more actively. In addition, scarcity is one of the important factors driving word-of-mouth in purchase situations. For example, when the number of products is limited but the price is affordable, some customers may feel that they are getting good value for their money when they receive the product and evaluate it positively, thus advertising the prepared dish company for free. This is particularly important for large companies, as only when brand recognition is established can customers be attracted to purchase the company’s products; therefore, prepared food companies need to pay close attention to the issue of product safety.

Third, create opportunities to trigger specific positive emotional responses. When consumers experience an unexpected positive experience, it tends to create a surprise emotion that in turn prompts word-of-mouth. This surprise effect depends to a large extent on companies continuing to innovate. Innovation can involve the product, packaging, production environment, consumption environment, and shopping process, and can be demonstrated at all stages before, during, and after the purchase. In addition, some companies endeavour to make consumers enjoy the taste of food at home and inspire a sense of well-being even when they are out of the care of the family, for example, by adapting food recipes several times. Based on this premise, companies can also complement word-of-mouth marketing campaigns to capitalise on the effects of voluntary customer advocacy.

Fourthly, it promotes positive consumer purchasing behaviour. While past research has focused mainly on the pre-purchase stage, the fact is that post-purchase behaviour is crucial to the long-term development of a business. If consumers find out that a product is of poor quality, tastes bad or has problems after they have purchased it, they will regret their decision and this negative experience will affect their negative impression of the brand image. On the contrary, consumers tend to be more satisfied with their initial choices after they have had a stunningly tasty experience while enjoying the product. This suggests that companies should pursue sustainable brand development with the strategic goal of achieving positive consumer buying behaviour. Truly effective word-of-mouth marketing relies on a foundation of quality products and superior goods and services delivered on the basis of customer needs, followed by targeted, well-designed and market-responsive word-of-mouth campaigns. Word-of-mouth is the result of a company receiving awards, free publicity, and attention for its outstanding products and services. It represents the free publicity and special attention that a company receives.

## Supporting information

S1 File (DOCX)
