# Supplementary material for: Word-of-mouth generated influences of different prepared dishes via online consumer purchases: Preliminary text-based research findings from "Jingdong Mall" flagship shops
Source: PLoS One. 2024 Mar 8;19(3):e0297972. doi: 10.1371/journal.pone.0297972 (PMC10923486; doi:10.1371/journal.pone.0297972)
Supplement: S1 File — (DOCX) [file pone.0297972.s001.docx]

| serial number | Word-of-mouth text (Luncheon meat A) | offerings |
| --- | --- | --- |
| A1 | In the Jingdong supermarket one-stop hoarding life goodies self-supporting purchase Wang Jiadu low-temperature luncheon meat, cooking, bread in the middle of the sandwich meat, shabu-shabu hot pot are particularly tasty, flavorful and tasty, more bells and whistles for dinner are tasty, it is my family preferred luncheon meat, will continue to support! Courier boy delivery especially fast! Powerful! | Original Flavor 640g |
| A2 | Wang Jiadu is my favorite lunch meat, but now the activity is less and less, and no longer possible to have the previous price, think back when the cheapest time almost ten dollars can buy a stick, now the lowest is also to 13.14, that is also much better than the usual price, early in the month to buy six, this time I bought 16, I have no place to put it! So the back of the ten selected the latest delivery time, before I rush to the fridge and freezer! | Original Flavor 640g |
| A3 | The first time I bought this brand of lunch meat, it seems to have opened the door to a new world Previously it was Merlin's lunch meat, this meat is so full of flavor Jingdong logistics is also very strong, home delivery is very convenient with a baby at home, we need this kind of convenient services | Popularity】Original Flavor 320g |
| A4 | Especially like to eat lunch meat, eaten a lot of brands, and finally now has been locked in the Wang Jiadu brand, because it is eaten all the best taste inside. Whether with instant noodles or under the hot pot and on the steamed bread are very satisfied. | Original Flavor 640g |
| A5 | [New customer taste] Original flavor 198g  Whether it is to eat and drink, or wear wear, make use of, have indicated that I am satisfied with the Jingdong self-supporting shopping on the delivery service and customer service handling. Whether it is to eat and drink, or wear wear, make use of, have indicated that I am satisfied with the Jingdong self-supporting shopping on the delivery service and customer service handling. Do not have to choose any other what brand, this brand is a word, good. Cold chain delivery, fast arrival. Not very convenient to eat, if the solo packaging is very convenient. Wang Jiadu of this low-temperature refrigerated lunch meat in the Jingdong self-supporting cold chain bought many times, has always been a high repurchase rate, this lunch meat meat content is high, and - some with starch lunch meat has a big difference, whether it is a hot pot or sandwiches, cold food can be eaten, hot food is even better, it is worth recommending. | New Customer Tasting】Original Flavor 198g |
| A6 | This square box of pork, I personally feel that the low-temperature luncheon meat is better than the box, the texture is very tender, the logistics is also very fast. Have bought several times, eaten delicious, and buy some for the elderly, fried rice, shabu-shabu hot pot are very good. | Original Flavor 640g |
| A7 | The lunch meat at Wang's Ferry is really superb! Super delicious!!! Cooking noodles In the morning for breakfast Brushing hot pot ～～～～ can be put in a gourmet Super delicious Scrambled eggs Scrambled eggs can also be put a little lunch meat The whole family loves it! | Popularity】Original Flavor 320g |
| A8 | Bought several times, very tasty, very fragrant, low-temperature cold food, the texture is fragrant, the big brand is trustworthy, very good, Jingdong logistics is also very fast, the same day, quality and quantity. | 【Store Manager's Recommendation】Chicken Original Flavor 594g |
| A9 | The date is very fresh, regular spare, easy to take out of the house, the summer will usually have some, fried fried fried cut any how to eat, the date is very fresh, regular spare, easy to take out of the house, the summer will usually have some, fried fried fried cut any how to eat, the | Popularity】Original Flavor 320g |
| A10 | I have purchased this low temperature lunch meat many times, my kids love it and it's very convenient for making sandwiches, but the additives are a bit too much, so you can't eat too much amount at once. The meat is soft and tender, better than any other brand. | Original Flavor 640g |
| A11 | Wang Jiadu lunch meat purchased many times, shabu-shabu hot pot, stir-fried vegetables mixed vegetables to eat are good, very fragrant, the flavor of the taste of lunch meat eaten as a child, every time there are activities will buy a few boxes of preparation, Jingdong Mall shipping delivery are very fast praise! | Original Flavor 640g |
| A12 | Have purchased multiple times. Love the flavor and even more so the tenderness and juiciness, very tasty. Whenever I'm craving for meat sausage, I immediately think of Wangjiadu Low Temperature Lunch Meat, which is delicious when eaten directly cut up or after frying. I prefer to eat it fried, flavorful, tender, juicy, great. | Original Flavor 640g |
| A13 | Wangjiadu's luncheon meat is particularly tasty, the meat is delicate and fresh, and each shipment is sent in an insulated box and warm bag to ensure the freshness of the goods. Children eat noodles and breakfast with this lunch meat, the flavor is good. | Original Flavor 640g |
| A14 | Wang Jiadu, low-temperature luncheon meat , product quality is very good, the ingredients are clean, the material is real, convenient and quick food, the product date is fresh, the packaging is intact, the courier service attitude is very good, the courier delivery speed is also very fast. | Original Flavor 640g |
| A15 | It is really so delicious! A must-have at home during the New Year's holidays, no matter whether it is eaten by yourself or entertaining guests is very tasty, and my relatives asked me where to buy it, saying that it tastes very good and is very fresh. Jingdong Fresh is very powerful, can deliver such delicious food. | New Customer Tasting】Original Flavor 198g |
| A16 | It's really delicious, I don't remember how many times I've bought it back, but I'm sure I'll buy it for New Year's festivals. Whether the elderly children or dogs are super love to eat ah! Dragon Boat Festival activities are very powerful, a lot more affordable than the supermarket. | [Store Manager's Choice] Black Pepper Flavor 320g |
| A17 | Wang Jiadu this brand of refrigerated packaging, the entrance is very delicate, than the Hormel and other brands taste a lot better, every time the Jingdong has activities, will order to buy tun some. Packaging is also very simple and generous. Satisfaction | Original Flavor 640g |
| A18 | Children especially love to eat this, breakfast cut a few slices to fry, with bread, it is very convenient, low-temperature lunch meat, the meat looks good, very fragrant, the activities will be stocked up when the Jingdong buy the most affordable well | Original Flavor 640g |
| A19 | Logistics and delivery fast, packaging intact, cold chain delivery temperature is appropriate, lunch meat within the package is also very good, big brands are trustworthy, the production date is new, many times enough to buy, but also the family favorite, support | New Customer Tasting】Original Flavor 198g |
| A20 | Date is very good, May 30, bought five, this brand of lunch meat bought five years, have always loved, air fryer heating flavor absolutely absolute absolute son, can also be cut into pieces fried rice to eat, shabu shabu hot pot can be! | Original Flavor 640g |
| A21 | So in love with their lunch meat, I stock up on some for every event. This is a low-temperature lunch meat, the meat is firm, tender and has a great meat flavor. Storage temperature is 0-4 ℃ for five months, I usually put frozen, can be stored for more than half a year, will not affect the taste. | Original Flavor 640g |
| A22 | Delicious, cheap, affordable and delivery is very good. If the price is cheap, it is very worth stocking up, usually eat pasta with a few pieces of lunch meat is really very satisfied, I hope to do more activities put! | Original Flavor 640g |
| A23 | Taste a very good lunch meat, in addition to expensive no problem, the taste is particularly great, tender meat, catch the Jingdong 618 activities to buy, cost-effective, direct delivery is very convenient, the service is very on point ~ | 【Store Manager's Recommendation】Chicken Original Flavor 594g |
| A24 | This lunch meat is definitely YYDS, it's very tasty, it's good for fried rice and burger embryo, and it's also very tasty when you don't have a dish to bake in the oven. Delivery was fast, refrigerated tupperware delivered. | Popularity】Original Flavor 320g |
| A25 | Wangjiadu low temperature lunch meat sausage, the flavor is very good, you can make sandwiches, meal egg noodles. Fry it will be more delicious, nourishing flavor, a mouthful of very satisfied. Lazy people preferred, fast delivery, you can stock up. | Original Flavor 640g |
| A26 | Bought, to the child, when the breakfast, very tasty, but also very convenient, Jingdong self-support, delivery ****, very good, are already my fifth repurchase, eat continue to repurchase again! | Popularity】Original Flavor 320g |
| A27 | This is a great brand that is very trustworthy and we buy it all the time in our house. Elderly, kids love it. Good taste. Can be eaten directly, but also can do risotto, do fried rice, coleslaw eat with a little very good. | Popularity】Original Flavor 320g |
| A28 | Eating food or have to recognize the big brand, with the assured, single fried to eat, add in are very tasty, especially fragrant, flavor is very fresh, the child is very like, there are activities when it is very cost-effective, while the activities of the cheap and hurry to stock up on some. | Original Flavor 640g |
| A29 | I've bought this lunch meat many times, it's the best lunch meat ever, it's so delicious when you fry it in a little bit of oil, it's so delicious, kids and adults love it, you can eat it directly, make sandwiches, it's all good. | Popularity】Original Flavor 320g |
| A30 | This lunch meat is very good, and not salty, very tasty, much better than the World Stick, the morning sandwich bread is very tasty ~ Jingdong logistics is very fast, the next day after the order arrived, the hard work of the courier master ~ | Popularity】Original Flavor 320g |
| A31 | Wangjiadu is really our meat heart good not only have meat flavor dry taste will not be very salty cost-effective many times back to buy black pepper flavor meal favorite delicious delicious delicious delicious can not have meat can not do without Wangjiadu lunch meat | [Store Manager's Choice] Black Pepper Flavor 320g |
| A32 | All stocked Wang Jiadu, hometown brand quality is guaranteed. Very good, the flavor is quite good, delicious doped. Jingdong is also convenient and cost-effective. Much better than other platforms, before a certain sound was tricked into buying a younger brother version, or regular electricity supplier does not come to false, no misleading. | Original Flavor 640g |
| A33 | Jingdong logistics is quite powerful, delivered to the insulated box delivered, or ice, bought a box, eaten back to buy, this lunch meat flavor is very good, I am fried rice with more, recommended, will buy back again. | Original Flavor 640g |
| A34 | Small packages are especially suitable for a family of three a breakfast, the meat is delicate and full, delicious, bread and milk is an excellent partner, enough to provide the body with the required protein and nutrition, will always buy back | New Customer Tasting】Original Flavor 198g |
| A35 | This Wangjiadu lunch meat is especially like the taste of lunch meat eaten as a child, then there are not so many preservatives, meat aroma is very full. Chicken flavor texture is better than pork, breakfast to half a steamed bun, Chinese breakfast can be more smooth than sandwiches and bread like this. | 【Store Manager's Recommendation】Chicken Original Flavor 594g |
| A36 | Wang's Lunch Meat, great flavor, good texture, very fresh, no impurities, adults and children in the family? all like it? , cooking noodles, making sandwiches? will all add a piece of it in! | Original Flavor 640g |
| A37 | This sausage is very good, I like it very much, the kids love it, it's convenient, it saves time, when you don't know what to cook, it's the best thing to fry it, it's a good deal when you buy it, you buy a lot of it. | Original Flavor 640g |
| A38 | Very good lunch meat, bought many times, children like to eat, the production is also convenient, pan frying a little on the good, do not have to put oil, very healthy, taste is also very good, very satisfied with a shopping, and the Jingdong Express is still as fast as ever service is also very good. | Original Flavor 640g |
| A39 | This low-temperature lunch meat from Wangjiadu is full of ingredients and matches the description of the product, it can be used for frying, stir-frying and stewing, and the 640g package is superb value for money and tastes quite good, so if you haven't tried it before, you can buy it with confidence. | Original Flavor 640g |
| A40 | I bought a piece during a previous event and stocked up on larger grams this time around! It's delicious in fried rice, hot pot, etc., or fried and put in bread! It's not greasy and has a nice texture! You can trust this brand of lunch meat! | Original Flavor 640g |
| A41 | Meizhou Dongpo quality has nothing to say, lunch meat flavor is great, containing more meat, salty and light moderate, less additives so the shelf life is short, usually the price is really not cheap, but catching up with the activities of the price that is quite forceful, Jingdong self-supporting this speed has nothing to say, the packaging is complete and unbroken! | Original Flavor 640g |
| A42 | This ham has a great taste and keeps coming back for more than the canned ones, it's just a little salty, it would be better with pasta, and it's a small piece with just the right amount of portioning, so you don't worry about wasting it. Children also like. Jingdong self-supporting things are really great, affordable, home delivery, very fast, praise praise praise praise praise praise praise praise | Original Flavor 640g |
| A43 | Wang Jiadu, low-temperature luncheon meat, product quality is very good; clean ingredients, with real materials, convenient and fast food, the product date is fresh, the packaging is intact, the courier service attitude is very good, the courier delivery speed is also very fast! | Original Flavor 640g |
| A44 | The store shipped very quickly, good service attitude, should be genuine, a very pleasant shopping (? -? -?) ?? The price is super favorable, the performance is also very good, perfect a shopping is genuine, and the same as the entity store, the price is also preferential, decisively turn to powder value for money, words do not say much, these four words have said everything! | Original Flavor 640g |
| A45 | I've purchased it numerous times, the meat is firm and not loose like some lunch meats, it's grainy. Recommended! Jingdong logistics yyds, morning order night to night order sleep wake up to! In the future shopping preferred self-supporting! | Original Flavor 640g |
| A46 | Commodity quality: the product is generally good packaging open solid, fast delivery, really super like, very supportive, the quality is very good, and the seller street described exactly the same, very satisfied, really like, completely exceeded the expectations of the shipment speed is very fast, the packaging is very careful, strict, the logistics company has a very good service attitude, the delivery speed is very fast, very satisfied with a shopping | Popularity】Original Flavor 320g |
| A47 | Meizhou Dongpo lunch meat home essential, good flavor, high lean meat content, add less, so the shelf life is short, frying and cooking and frying everything can be, both young and old, the activities of the price is strong, Jingdong self-processing this speed has nothing to say quite wow, cold chain logistics insulation effect is very good! | Original Flavor 640g |
| A48 | I got it! It's really great! I can't say enough about the quality. I'm very happy with it. Happy happy? Taking advantage of the activities to get cost-effective, fast delivery, well-packed, very good, new experience, praise praise praise! (* ^ __ ^ *) very satisfied with a shopping, value for money, more affordable than the entity store! | Original Flavor 640g |
| A49 | Jingdong self-supporting this speed has nothing to say simply unbeatable, the next day up to the name of rain or shine is trustworthy, the packaging is complete without damage very heart! Meizhou Dongpo produce high quality, lunch meat taste great, containing a high proportion of meat, short shelf life without coloring and flavoring to add more healthy, convenient to carry on the road! | Original Flavor 640g |
| A50 | N times to buy Wangjiadu low-temperature lunch meat, Jingdong self-supporting store order the next day, the courier brother delivered to the door, the speed is really fast, lunch meat fried vegetables remember to oil fry both sides, fragrant really good, will buy back! | Original Flavor 640g |
| A51 | Logistics is fast, delivered in a foam box with ice packs inside, frozen solid, not melted at all. This lunch meat is a must-have for breakfast in my family, and has been repurchased many times. It has a high meat content, and it is a good choice for frying when eating, cooking noodles, and making sandwiches. | Popularity】Original Flavor 320g |
| A52 | I just bought it a few days ago, it was my first time buying it, I used to buy another old brand. It's not bad, I feel like it tastes pretty good, and I read the description that it has a higher meat content, so it tastes pretty good anyway. | 【Store Manager's Recommendation】Chicken Original Flavor 594g |
| A53 | The date is quite fresh, I thought it was April, but it turned out to be May, and this shelf life is 5 months. Jingdong logistics is very fast and fresh. Looked at the flagship store is April, the price is slightly cheaper than this, but it is SF two days to now the weather is hot. Or Jingdong logistics is more quality. | [Store Manager's Choice] Black Pepper Flavor 320g |
| A54 | Bought a total of three, the other would like to buy, but there is no goods, too hot, after all, is a big brand of Wang Jiadu, order too many people! Dragon Boat Festival activities are very powerful, and the family at home to enjoy the food less Wang Jiadu! | Popularity】Original Flavor 320g |
| A55 | This luncheon meat from Wangjiadu Dongpo Restaurant is one of the varieties that will not be out of stock in the refrigerator at home, and can be used for sandwiches, sushi rolls, and noodle soups. I've recommended it to many friends and they all say it's good. | Original Flavor 640g |
| A56 | Fast logistics and distribution, packaging intact, cold chain delivery at the right temperature, the big brand is trustworthy, the production date is new, has been many times enough to buy, but also the family favorite, support | Original Flavor 640g |
| A57 | Wang Jiadu lunch meat must not need to say more, we should have eaten, lunch meat I only eat Wang Jiadu, the major supermarkets offline, but the activities of the Jingdong greater price fair delivery fast, but also delivery to the door don't mention how convenient! | Popularity】Original Flavor 320g |
| A58 | Previously bought a small package, to make sandwiches or hot pot for children are good, the meat is quite tender, occasionally eat or can be eaten, 618 activities are very powerful, more favorable than usual, I hope that more activities | Original Flavor 640g |
| A59 | Bought many times, before always in the supermarket Sam and other physical stores to buy, this time to take advantage of the 618 have promotional activities, also do not have to run to the physical store, online order express door to door, save trouble, the Jingdong logistics is always powerful, praise! Will continue to buy! | New Customer Tasting】Original Flavor 198g |
| A60 | I myself as a child also super like to eat lunch meat, now their own daughter also super like to eat, this is the taste of childhood, very good, is a little expensive, occasionally there are activities, the price is cost-effective. | Original Flavor 640g |
| A61 | Ah, this is what I have eaten, really is considered very tasty lunch meat, because it is soft, and then, uh, tender, in short, it is quite tasty, more delicious than I used to buy in the supermarket, to be tasty later will repurchase, is a little bit expensive ah, this thing how so expensive ah, you do some more activity, do activities, and then we'll be more Tuen a little bit | Original Flavor 640g |
| A62 | Lunchmeat only from Wangjiadu.  Low-temperature preservation.  Tender meat.  Fresh date.  Quality and quantity guaranteed.  Ideal for sandwiches, chow mein, fried rice | Original Flavor 640g |
| A63 | Has been many times back to buy Wangjiadu low temperature lunch meat, lunch meat meat firm, flavorful, easy to fry, Jingdong fresh June 1 activities, the price is preferential, a stockpile of several boxes slowly eat! | Original Flavor 640g |
| A64 | I've bought Wangjiadu's lunch meat many times, and when it comes to events, I have to stock up on a lot of lunch meat, and buy some from both the big and small boxes. It's very tasty, small package of lunch meat, the family's morning is enough, very convenient. | Original Flavor 640g |
| A65 | Wang Jia Du low temperature lunch meat is a regular food in my refrigerator, the meat is tender and tasty, often used in stir-fry, with homemade toast or burger embryo in the morning, with cucumber lettuce, and a cup of milk or coffee, full of energy. | Original Flavor 640g |
| A66 | I used to buy this sausage, I love it, and I bought a lot of boxes for the 618 event, so I'll keep them for a long time, they're good, they're reliable, and the logistics is fast, so I ordered them on the first day, and they were delivered to your door the next day. | Original Flavor 640g |
| A67 | This lunch meat is often purchased, the family loves to eat, used to shabu-shabu hot pot feel more delicious, but also very greasy, occasionally fried food is also good, is now very little activity, you need to be placed in the refrigerator to freeze! | Popularity】Original Flavor 320g |
| A68 | Previously bought in the supermarket Wang Jiadu lunch meat, eaten really amazed me, I did not expect this lunch meat kill all the domestic brands I have eaten before, and even not lose the foreign brands, meat content is large, meat flavor, no additives, lunch meat to eat that is elastic and glutinous, tender and juicy texture, full of meat. | Original Flavor 640g |
| A69 | Always buy this lunch meat, good flavor, lean meat, make sandwiches, or vegetable salad put some lunch meat are very good, the family loves to eat. It's a good idea to get some discounts on the price. | New Customer Tasting】Original Flavor 198g |
| A70 | Breakfast worried about it, buy some of everything, to the breakfast colorful some, do hand pancakes put some, make sandwiches put some, porridge put some luncheon meat granules, the child is very facetious, eat very good. It's also great for cooking instant noodles. | Original Flavor 640g |
| A71 | Another purchase of Wangjiadu lunch meat, delicious flavor, lean meat, can be used to make breakfast sandwiches, you can also do vegetable salad, are very good, the whole family likes to eat, is a little expensive, and then can be a little more favorable would be better! | Popularity】Original Flavor 320g |
| A72 | Friends introduced, said it was very good, the second time I bought, good quality, home children like, a lot more cost-effective than the supermarket, cold chain delivery, to the hand is cool, Jingdong distribution is very reliable. | Original Flavor 640g |
| A73 | King's Ferry's lunch meat, tops? s delicious. Eat it with breakfast bread, with baklava, with large pancakes, with steamed buns. It's also very good to make spicy hot pot and stir fry with green vegetables. Just wine is also very good. Stock up when the deals are great! | Original Flavor 640g |
| A74 | The first time to eat this brand of products, packaging design is very innovative, high-grade, chicken is very tasty, flavorful, not greasy, pork looks a little white meat, greasy texture, there is a meat fishy, I do not like, express delivery is very fast, thank you! | 【Store Manager's Recommendation】Chicken Original Flavor 594g |
| A75 | Ham Wang Jia Du is simply yyds, compared with his flavor personally think may be Sam's, but I still think this is the most fragrant. Long-term repurchase of good things, properly one of the main fast food, breakfast or dinner, a handful of fried on the OK! | Original Flavor 640g |
| A76 | Has been back to buy a lunch meat, high meat content, very satisfied with a shopping, business service is good, fast shipping, logistics is also very strong, baby received very like, buy peace of mind, with peace of mind! | Original Flavor 640g |
| A77 | Wang Jia Du Low Temperature Lunch menu meat. It is the original flavor of pork, read the ingredients and cooking method recommendations, and is . I bought it to try. I bought it to try. This morning, I took out the menu and wanted to eat a piece of it, but it's a whole piece! It's not very convenient to eat, it's very convenient if it's packaged separately. I'll open it next time when I can finish it in one go for other dishes. | Popularity】Original Flavor 320g |
| A78 | Wang Jiadu brand, completely at ease, this small package, a just enough for my family to eat a meal, not afraid to open on the dry bar, not wasteful very humane, breakfast fried two slices with fried eggs, bread, perfect. | 【Store Manager's Recommendation】Chicken Original Flavor 594g |
| A79 | This luncheon meat is very special, the ingredient list is relatively simple, see there are activities will buy some tuning, but this shelf life is only six months, or to be refrigerated, the taste is quite good, you can eat the meat, breakfast slices fried? Bread excellent. | Original Flavor 640g |
| A80 | Wang Jiadu's this low-temperature refrigerated lunch meat has been bought many times on the Jingdong self-supporting cold chain, and it has always been a high repurchase rate, this lunch meat has a high meat content, which is very different from some lunch meat with starch, whether it is hot pot or sandwiches, it can be eaten cold, and is even better when it is eaten hot, so it is worth recommending. | Original Flavor 640g |
| A81 | Whether it's food and drink, or wear and wear, make use of, all indicate that I am satisfied with the Jingdong self-supporting shopping on the delivery service and customer service handling. Whether it's food and drink, or wear wear, make use of, all indicate that I am satisfied with the Jingdong self-supporting shopping on the delivery service and customer service treatment. Do not have to choose any other what brand, this brand is a word, good. Cold chain delivery, fast arrival. Not very convenient to eat, if the solo package is very convenient. Wang Jiadu of this low-temperature refrigerated lunch meat in the Jingdong self-supporting cold chain bought many times, has always been a high repurchase rate, this lunch meat meat content is high, and some of the lunch meat with starch is very different, whether it's a hot pot or a sandwich, cold food can be eaten, and hot food is even better, it is worth recommending. | New Customer Tasting】Original Flavor 198g |
| A82 | Has been many times to buy Wangjiadu low-temperature lunch meat, breakfast, shabu-shabu hot pot, are very good side dishes, juicy, fat and lean ratio is appropriate, the taste is better than the general ham and sausage is too good, will come back to patronize! | Popularity】Original Flavor 320g |
| A83 | I've bought this Wang's Lunch Meat many, many times, it tastes great and the brand is reassuring! Bought it for my kids to make fried rice and sandwiches are especially delicious, and my kids love it! Jingdong logistics is also fast, will continue to buy back! | 【Store Manager's Recommendation】Chicken Original Flavor 594g |
| A84 | I love it so much, I've bought this product for four years now, for my family and relatives, it's super yummy, I love the air fryer to heat it up, and the oil stains are old and delicious! You can also fry rice or shabu shabu, just great! | Original Flavor 640g |
| A85 | Wang Jiadu's ham and sausage bought many times, used to buy pork, a lot of meat inside, just cut a little oil. This time to buy chicken to try to see, is also very good, worth recommending the purchase of | 【Store Manager's Recommendation】Chicken Original Flavor 594g |
| A86 | Multiple purchases, Jingdong self-supporting logistics is very fast. Activity price is cheaper than the entity store, the flavor is good baked fried shabu shabu when shabu shabu are very good, packaging is also very good two pieces of a box. Thanks to the courier brother every delivery before the phone contact in advance attitude is very good! | Original Flavor 640g |
| A87 | This bought many times, the flavor is not bad, not salty, usually do a hand pie, fried egg fried rice are very good with, is the need to put in the refrigerator, a bit of space, nothing to cut a few slices to put the noodles or use the air fryer to test a little is also very good, praise! | Original Flavor 640g |
| A88 | Commodity quality: the product is generally good, tight packaging, fast delivery, really super like, very supportive, very good quality, and the seller describes exactly the same, very satisfied, really like, completely exceeded expectations, the delivery speed is very fast, the packaging is very careful and strict, the logistics company has a very good service attitude, the delivery speed is very fast, very satisfied with a shopping. | Popularity】Original Flavor 320g |
| A89 | Very good, the taste is very tasty, the ingredients are sufficient, the taste is very fresh, the production date is sufficient, the quality control and quality is still very reliable, the elderly and children at home are very much like to eat, the next time will continue to buy. | 【Store Manager's Recommendation】Chicken Original Flavor 594g |
| A90 | This luncheon meat taste very good, since I saw on the Jingdong, in the Jingdong have already bought several hundred dollars of money, and recommended to my friends, delicious, but also can be rolled in the middle of the bread, great! | Original Flavor 640g |
| A91 | Jingdong logistics is super fast, the evening order, the next morning delivery, cold fresh products or insulation bag processing, get the hand is cold temperature, especially 618 activities affordable very large, good value for money family timely items renewed on the Jingdong self-supporting order, quality, speed, after-sales service are guaranteed, thank you Jingdong self-supporting. | Original Flavor 640g |
| A92 | Have been using this, very good, parents like to use, catch up with the 618 activities, more tuned a few bags, can insist on using to the double eleven no problem, the logistics speed is also very fast, bought yesterday's today can arrive. | Original Flavor 640g |
| A93 | The first time in Dingdong to buy, found that the Jingdong also have, and reasonable price, good value for money, delivery to the home is also very convenient, well-packed, the product quality is very good, good taste, a penny for a penny! | Popularity】Original Flavor 320g |
| A94 | Date is very good Oh, Jingdong self-supporting cold chain do very good, fast delivery, there is this kind of fresh to get the hand will not be bad at all, the big night courier brother will still be delivered, will also confirm whether the home delivery. Of course, the main thing is that there are activities when the price is too much cheaper than the super! | Original Flavor 640g |
| A95 | Jingdong logistics speed is fast, and the price of the event is favorable.  Wang Jiadu lunch meat, many times back to buy, good flavor.  Pork original flavor, there are two inside a box, make a sandwich in the morning, or shabu-shabu hot pot are very good, take advantage of the activities, buy 5 boxes at a time! | Original Flavor 640g |
| A96 | First of all, the packaging is very good, with a foam box, which is insulated with ice packs. In addition, I have purchased "Wangjiadu" luncheon meat many times, and it has a delicate and smooth texture, and the meat is tasty. It can be used for breakfast, shabu-shabu, or as a side dish in stir-fries. | Original Flavor 640g |
| A97 | I believe that this product, believe in health, professional athletes should not be bad choice, logistics is still super fast, in time for the event to get cost-effective, I hope that in the future, Jingdong can continue to launch more favorable activities. | 【Store Manager's Recommendation】Chicken Original Flavor 594g |
| A98 | This lunch meat flavor is very good, in addition to the price is a little expensive, nothing else is wrong, very like to eat is also very convenient, is the repurchase of many times the product, every time there is an event when the purchase is more cost-effective | 【Store Manager's Recommendation】Chicken Original Flavor 594g |
| A99 | Logistics is very fast, well-packed, high meat content, very satisfied with a shopping, good business service, fast delivery, logistics is also very powerful, baby received very much like, buy rest assured, with peace of mind! | Original Flavor 640g |
| A100 | Lunchmeat for sandwiches, Wangjiadu's is so good, you can eat it right out of the box every time, and it's so good with egg and cheese slices, and it's so good to get it right away. | Popularity】Original Flavor 320g |
| A101 | This lunch meat is quite delicious, we have bought a lot of times, we like this, put a little when cooking noodles, air fryer frying is also very good look! | New Customer Tasting】Original Flavor 198g |
| A102 | Delivered when the insulated box was delivered, or ice, this time it is a repurchase, this lunch meat flavor is very good, so a one-time purchase of 5 boxes, so that counts 32 a box, very cost-effective, very recommended, and will be repurchased again! | Original Flavor 640g |
| A103 | Inexpensive, fresh date, children especially like this, cut into small pieces, air fryer 200 degrees for 4 minutes, the outside of the scorched inside, and then sprinkle some cumin, don't mention how fragrant, eat the beautiful. | Original Flavor 640g |
| A104 | The whole family loves to eat lunch meat, repurchase many times, whether it is to make sandwiches, or hot pot to eat, are super tasty, Dad is often used to make fried rice, very fragrant, high meat content, eat a little starchy feeling are not it! | 【Store Manager's Recommendation】Chicken Original Flavor 594g |
| A105 | Wangjiadu low-temperature lunch meat, Renxin brand, quality is absolutely guaranteed, rich flavor, great taste look forward to activities, offers continue to recommend friends and relatives to share! | Original Flavor 640g |
| A106 | After careful comparison, after a long time of screening, I finally chose this product, the quality of the stuff is very good, the delivery speed is very fast, the staff's service attitude is very good, remember to engage in activities when you buy Oh. | 【Store Manager's Recommendation】Chicken Original Flavor 594g |
| A107 | Wang Jiadu cryogenic lunch meat is a convenience food that is often repurchased at home, the quality of the big brand is good, safe to eat, the lunch meat is ready to eat in the open bag, eat alone, with a meal or cook noodles with a very tasty. | Popularity】Original Flavor 320g |
| A108 | Bought this lunch meat many times, especially delicious, the children also love, every weekend to eat sandwiches, a hundred times to eat, no starchy texture, the activities of a little more will be better, recommended! | Original Flavor 640g |
| A109 | Found the most delicious a lunch meat, the original has been to buy the world stick, too salty, but also a little meat fishy flavor, this brand is not, the family children and adults are very much like, the activities of the price is super affordable, cheaper than half of the box horse. | Original Flavor 640g |
| A110 | Wang Jiadu low-temperature lunch meat original flavor 640g ready-to-eat breakfast sandwiches meat sausage sausage hot pot shabu ingredients convenient instant food is the first time to buy, 320g a package, the size is appropriate, after frying and grilling is quite tasty, a little bit of salty, it is very good. | Original Flavor 640g |

| serial number | Word-of-mouth text (Grilled sausage B) | offerings |
| --- | --- | --- |
| B1 | This King's Ferry Popping Sauce Grilled Sausage has been repurchased again by my family. Overall, the texture is good, and I feel that the meat content is also very acceptable, and the original flavor is not too salty and not too light just right. Steamed and grilled are good. | 【Store Manager's Recommendation】Grilled Sausage with Popping Sauce 480g |
| B2 | Meat is very tasty, unlike the outside of the starch sausage, this flavor is very tasty Oh, it is worth buying, Wang Jiadu's really different, lunch meat is also very tasty ah, and is still our Meishan enterprises, support, with my silver ear soup, very nice | 【Store Manager's Recommendation】Grilled Sausage with Popping Sauce 480g |
| B3 | Wang Jiadu Superior Popping Sauce Grilled Sausage, very good quality, tender meat, fresh date, packaging intact, the whole cold chain transportation, received without thawing. Courier service attitude is very good, courier delivery speed is also very fast. | 【Store Manager's Recommendation】Grilled Sausage with Popping Sauce 480g |
| B4 | Not bad not bad oh! Bought it twice now. There are activities to get down! Good good good oh! Bought twice. There are activities to get down! Good good good oh! Bought twice. There are activities to get down! | 【Store Manager's Recommendation】Grilled Sausage with Popping Sauce 480g |
| B5 | This sausage has been repurchased many times, the meat inside is real and chewy when steamed, delicious. It can be stir-fried vegetables, fried rice, both with good. The meat is the right amount of fat and lean, and I'll buy it again in the winter when I'm done. | Savory Sausage 440g |
| B6 | At that time in order to make up the ingredients to order, and then eat actually very good, there is a light smoky flavor, the children like to eat, leftovers the next day in the pan fried is also very tasty, very recommended. | Hot Pot Series] Hot Pot Crispy Sausage 480g |
| B7 | The product is after a long time of research, but also asked friends who had purchased, all feel very good, and the Internet to look at the relevant comments, it seems to buy is very assured, buy the outer packaging looks very beautiful, the product is also good. | 【Store Manager's Recommendation】Grilled Sausage with Popping Sauce 480g |
| B8 | Usually often eat grilled sausage, the first time to buy this brand, which is a small package, easy to eat, processing methods are varied, single food or fried vegetables can be, the flavor is not bad, it is very convenient to shop in the Jingdong, delivery is very fast. | 【Store Manager's Recommendation】Grilled Sausage with Popping Sauce 480g |
| B9 | My friend introduced it to me and strongly recommended I try it, and I just happened to see it in this order, so I bought one. Should be good, do dry pot when put in, soon to be eaten up, but unfortunately did not taste. Jingdong delivery, quickly received. | 【Store Manager's Recommendation】Grilled Sausage with Popping Sauce 480g |
| B10 | Before I bought a la, feel very good, buy back again. Inside is a small package of 4 small packages, very convenient, mainly children like this small sausage, hot pot essential, Jingdong order is super convenient and hassle-free. | Hot Pot Series] Hot Pot Crispy Sausage 480g |
| B11 | Shopping at Jingdong Mall is convenient and fast, ordered last night and the courier delivered this morning. The quality of the food is guaranteed, the box is clean and tidy, and the food tastes good???? , after eating continue to purchase. | 【Store Manager's Recommendation】Grilled Sausage with Popping Sauce 480g |
| B12 | This is the first time to buy this brand of kielbasa I tried it in the air fryer when it arrived, and it's just as the picture says, it's got a lot of juices and a lot of meat, and it's very satisfying when you bite into it, so I'll buy it again when it's finished. | 【Store Manager's Recommendation】Grilled Sausage with Popping Sauce 480g |
| B13 | Baby received, very surprised, the quality is very good, very satisfied with a shopping. I will continue to buy back, the store is trustworthy. Jingdong self-support is trustworthy, and the logistics speed is also very fast, recommending that friends in need of purchase! | 【Store Manager's Recommendation】Grilled Sausage with Popping Sauce 480g |
| B14 | I bought this sausage** omelette and said that there are several ways to make it, but I think the most reliable is to fry it in the oven or air fryer, it's very difficult to fry it in a pan. Taste is good, a band inside divided into four small bags individually packaged, quite convenient. | 【Store Manager's Recommendation】Grilled Sausage with Popping Sauce 480g |
| B15 | Wang Jiadu Dongpo spicy sausage meat is fresh, spicy and spicy, pleasant aroma, worthy of a big brand, authentic flavor! Bought three times in a row, each time to buy two packages of spicy sausage, but also bought other sausage, the holidays to the family, everyone said it was very tasty! Logistics fast, the night before the order was received the next day, courier delivery, good service attitude, commendation! | Spicy Sausage 440g |
| B16 | The flavor is quite good, the texture is good, it will burst with juice and it is full of meat, it is a little small, it is only possible to eat two at a time. Courier delivery to the inside are dry ice, not frozen, and the logistics industry is strong, the next day to receive the goods. | 【Store Manager's Recommendation】Grilled Sausage with Popping Sauce 480g |
| B17 | Buy a good grilled sausage, with seasoning, black pepper, etc., in the preparation of the air fryer, you can wait for the delicious out of the Sam's supermarket members recommended, worth having, looking forward to delicious grilled sausage! | 【Store Manager's Recommendation】Grilled Sausage with Popping Sauce 480g |
| B18 | Someone else recommended the popping juice grilled sausage. I just followed up brainlessly. The logistics was still pretty fast, I received it the next day after ordering. The date is okay, the shelf life of 12 months.  The taste flavor will come back to write additional comments when I finish it. I hope it can satisfy me. | 【Store Manager's Recommendation】Grilled Sausage with Popping Sauce 480g |
| B19 | The first time to buy it ...... packaging is good, small packages are also convenient, put into the air fryer bake a bake, or pan fry, can be, quite delicious, hahahahahahahaha, may be back to buy. | 【Store Manager's Recommendation】Grilled Sausage with Popping Sauce 480g |
| B20 | Looked at a very large box, but there are only 12 sausages inside, is not much, the date is relatively fresh, the specific flavor and so on cooking to know, after eating in the back of the back to comment on the Wang Jiadu things slightly lower cost, but overall it is quite good, it's worth trying! | 【Store Manager's Recommendation】Grilled Sausage with Popping Sauce 480g |
| B21 | This Wangjiadu, we have been buying that sausage, grilled sausage for the first time, not particularly surprised, the taste is okay, now I can not find any good sausage, the juice is not too much, the price is not very cheap. | 【Store Manager's Recommendation】Grilled Sausage with Popping Sauce 480g |
| B22 | This brand is really the first time to eat, meat and flavor, feel the North Street sold on the volcano stone barbecue sausage is delicious, the quality is also assured, the price is cheaper than the supermarket point, take out the air fryer 10 minutes to eat, the | 【Store Manager's Recommendation】Grilled Sausage with Popping Sauce 480g |
| B23 | This brand of things are pretty good, before the low-temperature slow-cooked lunch meat and this crispy sausage taste pretty good, grilled taste better, the child is also more acceptable, will come back to buy! | Hot Pot Series] Slightly Spicy Crispy Sausage 480g |
| B24 | Purchased several times, looks a little fat, but after cooking the taste in the mouth is just right, almost finished before I remembered to evaluate, it seems to be back to buy. By the way, the sausage is very good to go, I do not eat sausage, this is very good! | Savory Sausage 440g |
| B25 | In Sam bought a spicy flavor, but the rest of the family can not eat, this time specially in Jingdong to buy a savory flavor, taste good, I feel that it is still spicy delicious, Wang Jiadu is the bottom of the Meizhou, trustworthy, sausage fat and thin, oil but not greasy, very gluttonous! | Savory Sausage 440g |
| B26 | Made spicy hot pot to eat, very tasty, very much like this sausage, will come back to buy, Wang Jiadu brand is really good Oh, a good experience, repurchase must repurchase it, will also buy other dishes to try under the | 【Store Manager's Recommendation】Grilled Sausage with Popping Sauce 480g |
| B27 | Wangjiadu burst juice grilled sausage or the first time to buy, more attractive to me is inside is independent of 3 a package, for me very good, every time a big packet of open to eat waste, this is very suitable. | 【Store Manager's Recommendation】Grilled Sausage with Popping Sauce 480g |
| B28 | This crispy sausage from Wang Jia Du tastes good? A box of 4 small packets, used to often buy to shabu-shabu hot pot to eat, this time hit the flower knife fried to eat, put a little pepper salt, and fried chicken pieces together, is a good dish. Jingdong delivery fast! | Hot Pot Series] Hot Pot Crispy Sausage 480g |
| B29 | Wang Jiadu also out of the roasted intestines, while the Jingdong Mall to do activities hurry to buy to taste, a box of three bags of a total of 12, with the air fryer to do, the outside of the charred inside, a mouthful of burst juice, the flavor is really good. | 【Store Manager's Recommendation】Grilled Sausage with Popping Sauce 480g |
| B30 | Lunch meat is the children's favorite, every time many packets a buy. Jingdong's things are trustworthy, and the platform is also trustworthy. Often buy a lot of single together. Cost-effective is very high. Will buy back | Hot Pot Series] Hot Pot Crispy Sausage 480g |
| B31 | The best bacon and sausage in the world, love it, love it too much, keep repurchasing, be kind to yourself during the epidemic, eat this sausage and bacon is very appetizing, thank you, the flavor is rich and sweet and delicious and fresh Logistics is very fast, hard work, will buy again. Cheer up. | Savory Sausage 440g |
| B32 | This ham will be a little spicy flavor, suitable for adults, children are still difficult to accept, adults can be used for breakfast, cooking is good, good taste. Think this brand is still very good, will come back to buy the original flavor | Hot Pot Series] Slightly Spicy Crispy Sausage 480g |
| B33 | The kids can't go wrong with steak and sausage for breakfast but the brand of sausage they always buy the kids are tired of it they've had their sausage and this is the first time I've tried this kind of grilled sausage it tastes so good when you fry it in oil it's full of meat and the kids like it superbly | 【Store Manager's Recommendation】Grilled Sausage with Popping Sauce 480g |
| B34 | Repurchase many times the salami, although a little expensive, but the taste is really good, the meat is also very fresh, spicy flavor is very positive, seasoning and meat are of good quality, absolutely guaranteed, big brand trustworthy. | Spicy Sausage 440g |
| B35 | This Wangjiadu grilled sausage we have not the first time to buy in this, the flavor that goes without saying, it is too good, grilled to eat a little bit of fire, the kind of bubbling oil and the charred flavor of the meat is particularly good! | 【Store Manager's Recommendation】Grilled Sausage with Popping Sauce 480g |
| B36 | This product is very tasty, our family are very like to eat, with a variety of cooking methods, the price are quoted flavor super invincible delicious, baked to eat will squeak oil, we eat the end of the store will also be purchased! | Hot Pot Series] Hot Pot Crispy Sausage 480g |
| B37 | Wang Jiadu Hot Pot Crispy Sausage 480g (120g*4) Crispy sausage sausage spicy hot pot hot pot hot pot barbecue shabu frying ingredients brand products, trustworthy, in this store to buy several flavors of barbecue sausage, are very tasty. | Hot Pot Series] Hot Pot Crispy Sausage 480g |
| B38 | Brand products, trustworthy, well packaged, easy to take, inside the vacuum packaging, eating is also convenient, clean and hygienic. The taste is delicious, adults and children like to eat, frying and cooking, are very convenient. | 【Store Manager's Recommendation】Grilled Sausage with Popping Sauce 480g |
| B39 | Previously in the Sam shop inadvertently bought, which is very tasty, the taste is very much like Yu is almost like the sausage, oil and not greasy, especially to relieve the craving, this time also bought savory, if you can eat spicy or spicy delicious, three can be steamed a plate, it is worth saying that the Jingdong delivery is cold chain, packaging is very good, the gift of hot pot sausage, and so on to eat the hot pot time and then try it again! | Spicy Sausage 440g |
| B40 | Jingdong order is so convenient, do not have to go to the store can also eat authentic sausage. Especially like the original flavor, and the taste of the store no difference. Clean and hygienic delivery speed, delivery to the door, to the home is still frozen. | Savory Sausage 440g |
| B41 | Meizhou Dongpo sausage is very good, often go to the store to eat, eat and buy some to take home, inadvertently saw the Jingdong flagship store to buy very happy. Fast shipping, door-to-door delivery tastes the same as the store. | Spicy Sausage 440g |
| B42 | very good the child is older sometimes eat several pieces in the morning quite delicious I hope more activities to buy the right This is sold in the supermarket is very expensive, but the best to buy the best cheap the next day can be delivered | Hot Pot Series] Slightly Spicy Crispy Sausage 480g |
| B43 | The sausage that I've been buying again and again, my parents are getting old and have no teeth, they can eat it, and it tastes great, it's fresh, it's better than the supermarket, it's delivered to your door, and now it's delivered in two hours, it's great. | Savory Sausage 440g |
| B44 | Wang Jiadu's Dongpo sausage 440 grams of salty and fresh flavor sausage received, a very tasty meat products, fresh meat made without additives and preservatives and other nutritious and healthy, the taste is also very good, the family often purchased things, the date of production is fresh, the merchant shipment speed is very fast, the logistics company the whole frozen transport delivery to the home is very good. | Savory Sausage 440g |
| B45 | Wangjiadu's lunch meat has been a consistently repurchased item, whether it's hot pot or fried breakfast, it's very convenient and delicious, this time I want to try how their grilled sausage is, and buy a box to try it. | 【Store Manager's Recommendation】Grilled Sausage with Popping Sauce 480g |
| B46 | Six stars, one more star is not afraid of your pride, still hesitant friends hurry up, conscientious recommendation, really tasty, cost-effective and high, the future is still in this buy. In short: satisfied! Satisfaction! Satisfied! | Spicy Sausage 440g |
| B47 | This grilled sausage, there are 4 bags in one package, each bag contains 3 sticks, thaw and put in the oven or air fryer, very convenient. The ceiling of the grilled sausage world and the flavor is amazing. Never eat outside kielbasa again! | 【Store Manager's Recommendation】Grilled Sausage with Popping Sauce 480g |
| B48 | This fondue sausage can be put in the fondue pot and cooked, or fried.  The flavor is indeed quite good, you can also bake, do breakfast when frying a few, with bread, milk nutrition is very good, the child is very good, it is worth recommending, the next time you will buy. | Slightly Spicy Flavor】Multi-meat Hot Pot Sausage 480g |
| B49 | Stock up before the holidays, this brand is also an old brand, Dongpo sausage is very tasty, and the store to eat the same taste, convenient dishes are very provincial, at home can also do a big dish, especially suitable for lazy people cooking, worth buying. | Savory Sausage 440g |
| B50 | The flavor of this kielbasa is still quite good. The meat is tender. It also comes out well in an air fryer. Use it on its own, or. With bread, greens, carrots and other ingredients. It's perfect to make into a hot dog for breakfast. | 【Store Manager's Recommendation】Grilled Sausage with Popping Sauce 480g |
| B51 | Wangjiadu Dongpo sausage, spicy flavor, 440 grams, a big pack, enough to eat for a while, it looks colorful and flavorful, 5 stars. | Spicy Sausage 440g |
| B52 | Wangjiadu's grilled sausage is particularly good, and I used to buy it regularly. It's good for shabu-shabu or frying in a pan, the main thing is that it has a special flavor and is also very soft, the kids especially like it and will continue to buy it in the future. | 【Store Manager's Recommendation】Grilled Sausage with Popping Sauce 480g |
| B53 | The order was taken in the evening and delivered the next morning, which is OK for speed and cold chain delivery. This sausage is the non-spicy one, which is the same as the one sold in the Meizhou store at a much cheaper price, and it comes with a free hot pot crispy sausage! | Savory Sausage 440g |
| B54 | This sausage is very tasty, and Meizhou Dongpo restaurant to sell the flavor is almost the same, the key is the sausage steamed without odor, very fragrant and delicious, the price is better than the supermarket to sell the cost-effective to be high, before the Spring Festival to buy 2 bags are eaten, after the Spring Festival to see the seller and stocked, grabbed another 2 bags, this time the store also sent a gift, thank you very much to the seller! | Savory Sausage 440g |
| B55 | The food in this store is very good, the grilled sausage can be grilled or fried, the flavor is very good, the crispy sausage shabu shabu tastes good and the texture is great! I will continue to buy from this store when I'm done eating. | Hot Pot Series] Hot Pot Crispy Sausage 480g |
| B56 | Already received it, very good, there are freebies, cost-effective too high ah, love love love, fried vegetables stewed rice are delicious, slightly spicy, super flavor, buy back many times ah, there are activities on the stockpile point, very satisfied! | Spicy Sausage 440g |
| B57 | Arrived on the can't wait to open, really did not let me disappointed, the quality is particularly good, the value is also quite high, satisfied with a shopping, repurchase many times, there are activities on the stockpile a little, casserole to eat adults and children like! | Savory Sausage 440g |
| B58 | Jingdong self-supporting platform, convenient logistics, courier brother serious and responsible, meat is very good, pure pork, taste authentic Sichuan flavor, spicy and fresh,, the date is very fresh, the activities of the price is affordable, many times back to buy! | Spicy Sausage 440g |
| B59 | buy a little more at a time children love to eat things to share together and grow up happy Jingdong self-managed logistics is very powerful arrival fast delivery fast to the home are still frozen not melted very satisfied with more to do a little preferential activities more perfect it | 【Store Manager's Recommendation】Grilled Sausage with Popping Sauce 480g |
| B60 | This bought a few times, the flavor is particularly delicious, and before in the Meizhou Dongpo restaurant store to eat the same taste. But the price is much more affordable than eating outside, this is also a special price to buy to stock up, but also sent the crispy sausage, good. | Savory Sausage 440g |
| B61 | Very good taste, breakfast to a very convenient, can be fried can be examined, sometimes steamed things by the way together with the steam is also very good, believe the quality of the brand, the whole family love to eat. | 【Store Manager's Recommendation】Grilled Sausage with Popping Sauce 480g |
| B62 | Wang Jiadu's popping juice grilled sausage packaging is very complete and tight, the meat of the grilled sausage is very rich Q elastic, great taste, the ingredient list inside the additives are also less, eat especially rest assured, overall very satisfied. | 【Store Manager's Recommendation】Grilled Sausage with Popping Sauce 480g |
| B63 | This spicy sausage from Meizhou Dongpo is especially good, just like the ones from the restaurant, I don't like the savory ones, I like the spicy ones, once I eat it, I'm addicted. This frozen is also good, keep longer! | Spicy Sausage 440g |
| B64 | Each one of the meat is very sufficient, the flavor is also very fragrant, after baking out of the oil nourishing. Eat in the mouth only meat flavor, especially strong, the production time is also relatively new, or relatively fresh, breakfast and dinner are like to eat! | 【Store Manager's Recommendation】Grilled Sausage with Popping Sauce 480g |
| B65 | Distribution is very fast, and fresh delivery with ice, very good, after unpacking there is no abnormality, do boiled rice put in a little very fresh, the flavor is also very authentic, is currently on the market is very good | Spicy Sausage 440g |
| B66 | Jingdong attentive outer packaging to ensure that the food packaging is intact, cold chain transportation products, the site is already in the case of insufficient manpower express delivery, kudos. But received the food flavor of young and old, Meizhou Dongpo brand, Wang Jiadu, taste very good, I hope the business owner has the opportunity to promote more. | 【Store Manager's Recommendation】Grilled Sausage with Popping Sauce 480g |
| B67 | Tried the savory sausage of the Dongpo Hotel Wangjiapo, always thinking of spicy flavor, as soon as I saw the shelves immediately buy on, delivery is very timely, arrived within twenty-four hours! Immediately steamed a taste, the flavor is very positive! | Spicy Sausage 440g |
| B68 | It is worthy of the popping juice grilled sausage, there are large chunks of meat inside, rich meat, chewy, bite has full of juice, meat flavor, very tasty, additives are not too much, recommended to buy. | 【Store Manager's Recommendation】Grilled Sausage with Popping Sauce 480g |
| B69 | Jingdong shopping is very convenient, this is really delicious, Jingdong overall service is very good, and the follow-up will be purchased from Jingdong. It is very affordable, and the after-sales service is very good. It is worth recommending. The grilled sausage is also delicious. Oven roast | 【Store Manager's Recommendation】Grilled Sausage with Popping Sauce 480g |
| B70 | Very tasty grilled sausage, too much better than the outside unknown brand of grilled sausage, their own home grilling, reliable quality, safe and secure. Less starch, good flavor, small meat particles inside, especially delicious. | 【Store Manager's Recommendation】Grilled Sausage with Popping Sauce 480g |
| B71 | Still a big brand to eat at ease, the quality is also guaranteed, especially in time for the event when the purchase is more cost-effective. The taste and flavor are very good, and the physical store is the same. Delivery is also very fast, you can have the opportunity to Tuen some. | Savory Sausage 440g |
| B72 | When I ate at Meizhou Dongpo, I found this sausage is very delicious, so I bought some on Jingdong, during the epidemic supplies are very little, all rely on this sausage for me to eat, I have to say, it is really a godsend to eat ah! | Spicy Sausage 440g |
| B73 | Eating delicious, immediately ordered a second package, hemp hemp strength is very full, spicy flavor is also very fragrant, the family ate also feel good. I hope the next time there will be a discount ah, years ago the price is not cheap. | Spicy Sausage 440g |
| B74 | Exceeded expectations, the quality is very good, fine workmanship, satisfied with the special special great, comparison of three families before buying, the store host is very good, answering the question is very patient and detailed, there is a need will come again? Catch up with the time to do activities to buy, this price is worth every penny? , from the outside looking very beautiful? The material and workmanship are good? , feel buy value, and logistics super fast, praise! | Hot Pot Series] Hot Pot Crispy Sausage 480g |
| B75 | I buy some of this Szechuan sausage every winter, unwrap it and steam it for dinner, it's a great meal. The sausage is of good quality, fat and thin, and tastes great. I don't want to eat too much pickled food, but it's not a big deal to eat a little bit now and then! | Spicy Sausage 440g |
| B76 | Very delicious, love this Sichuan sausage from Meizhou Dongpo, always eat, and Meizhou Dongpo restaurant store taste exactly the same! Jingdong logistics fast and powerful very satisfied, well-known brand products are trustworthy! | Savory Sausage 440g |
| B77 | Original and authentic, delivered to your door, convenient and trustworthy! Meizhou Dongpo's sausage is very good, and it has been ordered many times in Meizhou Hotel! This time I found it in Jingdong and bought many bags at once. | Savory Sausage 440g |
| B78 | Meizhou Dongpo sausage spicy flavor this taste is particularly good spicy taste is very strong taste is very good the quality of its products is very good I have bought a lot of times all kinds of ways are delicious Jingdong's logistics is very fast delivery to home | Spicy Sausage 440g |
| B79 | Often go to Meizhou Dongpo restaurant to eat, every time must point of the product, these two sausage flavor is really very good, the choice of ingredients is very good, the production process to pay attention to, very clean, the food is a big health event, Meizhou do very well ~ ~ | Spicy Sausage 440g |
| B80 | Delicious to burst, true to its name, before not buying all the regret, now as long as there is an event to get together storage, a box of two boxes are early to do. Genuine food-grade products, much better than other stores, the next time continue ~ ~ ~ | 【Store Manager's Recommendation】Grilled Sausage with Popping Sauce 480g |
| B81 | Wangjiadu's salami is so good, a little spicy but to an acceptable degree ！！！！！ Very fragrant ！！！！！ It's good with rice rolls, and the stir-fry is also good ！！！！！！！ | Spicy Sausage 440g |
| B82 | Meizhou Dongpo Restaurant's food flavor is still good, craftsmanship is exquisite, the materials used feel good, but the price is expensive, if there is no activity, really don't want to buy, I hope to bring the price down, do not have to engage in activities to be cheaper, and I hope to become a daily consumables ~ | 【Store Manager's Recommendation】Grilled Sausage with Popping Sauce 480g |
| B83 | I do not know if the packaging has been changed, but overall, the taste is still good wax cicadas, if the flavor of the Meizhou Dongpo Wangjiadu should be more trustworthy, is able to be cheaper would be good! | Spicy Sausage 440g |
| B84 | The flavor is okay, the taste is not bad, not as greasy as other brands, maybe it's my subjective feeling, it's okay, a small piece won't be afraid to eat it,. Easy to store and easy to eat in one meal. | Savory Sausage 440g |
| B85 | The taste is okay, quite suitable for our side of the taste, but it is too much fat meat, white, eat two slices will be tired. Jingdong Express is good, cold chain delivery, no damage, quite good. | Savory Sausage 440g |
| B86 | I got a small box of Wangjiadu Premium Popping Sauce Grilled Sausage as a freebie when I bought Wangjiadu Lunch Meat, and once I ate it, it was still really good! This time, I bought three big boxes, so I had to grill them to get the unique flavor. | 【Store Manager's Recommendation】Grilled Sausage with Popping Sauce 480g |
| B87 | The price is cheap and sufficient, good things to get every day. Previously purchased the product, this time to take advantage of the activities, without hesitation to order the product, packaging simple and stylish, good taste, beautiful appearance, or a good good good product. | 【Store Manager's Recommendation】Grilled Sausage with Popping Sauce 480g |
| B88 | First of all, the same city courier is fast, delivery within 2 hours, secondly, the quality of the best, the raw materials are exquisite, 0 additives, no preservatives, healthy and nutritious, and finally, after steaming, the taste is authentic, light salty taste accompanied by mouth meat aroma, fat but not greasy, thin but not firewood, good taste. | Savory Sausage 440g |
| B89 | I hadn't bought this sausage before, but when I saw that there was an event and it was written that the national sports teams also ate this sausage, and that Wangjiadu's products are very hot these days, as well as the pavilions are open quite a lot, so I shouldn't go wrong, so I bought several boxes to try. | 【Store Manager's Recommendation】Grilled Sausage with Popping Sauce 480g |
| B90 | Taste and flavor: very tasty Must be grilled to burst juice, high heat, no need to thaw grilled to burst juice is delicious. Not grilled to burst juice will not have juice, do not know why, once is not grilled burst, the flavor is general, but grilled burst super super delicious and juicy! | 【Store Manager's Recommendation】Grilled Sausage with Popping Sauce 480g |
| B91 | This Meizhou Dongpo sausage is a gift for my parents, I bought a copy first, tasted good, much better than our family in previous years to do their own taste, but also save a lot of trouble! Next time I will buy, buy food or recognize the old brand! | Spicy Sausage 440g |
| B92 | This year, the family did not make their own sausage, ready to buy online, choose to choose to choose this Wangjiadu Dongpo restaurant with the same sausage, after all, is the same type of Sichuan restaurant, quality, taste are guaranteed, and really did not let our family disappointment, the sausage finished home-made. As Jiangsu people prefer this savory flavor, New Year's Eve dinner the whole family dried out a plate? | Savory Sausage 440g |
| B93 | Wangjiadu's lunch meat and sausages are good. The ingredients are of high quality, no sinewy impurities, and the workmanship is fine, so it tastes good and has a good texture. The shelf life is one year, so you can buy with confidence. | 【Store Manager's Recommendation】Burst Sauce Grilled Sausage 480g |
| B94 | Love this spicy Sichuan sausage! Although the savory one is also good, but the spicy one is more flavorful and numbing. It is close to the New Year's price is not cheap, next time to do activities if you buy a little more. | Spicy Sausage 440g |
| B95 | Classmates parents introduced, really good, with the air fryer baked ZiZi burst, with chili noodles really can not stop, bought a box, feel not enough to eat ah! Jingdong logistics, received the goods on the same day, used an ice pack, to the hand is still frozen. | 【Store Manager's Recommendation】Grilled Sausage with Popping Sauce 480g |
| B96 | It's the closest one I've ever had to the flavor of the delicious sausage in my memory, and I've repurchased it many times, except for one time when I bought it in the summer, and the sausage's flavorful texture was slightly worse than the other times, it's been good the other times. Cut a section and steam it with rice, so fragrant and delicious! | Savory Sausage 440g |
| B97 | Packaging is very good, the value is good, the quality is not a problem Shipping is very fast, the packaging is very good, the price is particularly affordable, there are so many efficacy is also good, it is worth buying quality is very good? , cost-effective and relatively high quality goods? , like the pro can directly get down do not hesitate oh? | Savory Sausage 440g |
| B98 | Sausage only believe in this brand, the taste is the same as the store dine-in, delicious, always also buy only savory flavor. To the field parents also often send, Jingdong quickly reliable. It's time to stock up again, almost out of food! | Savory Sausage 440g |
| B99 | Meizhou Dongpo Jingdong self-owned flagship store has two types of sausage, savory flavor and spicy flavor, I buy every time two kinds, this is 440g loaded spicy flavor, the family's great love, the taste of spicy and fragrant, fat and not greasy, full of meat, steamed rice when washed and wiped dry spread on the rice, steamed out of the rice are full of drops of sausage flavor, sausage on the recognition of his family? Jingdong has always been so trustworthy? | Spicy Sausage 440g |
| B100 | Meizhou Dongpo Jingdong self-flagship store of this salty and fresh taste of Sichuan sausage, a bag of 440g , there are six, packaging in place, good quality, clean, fresh taste, meat full, can also be used for stir-fry, fried rice, is my family's favorite, as long as the food is finished will be purchased, Jingdong has always been so trustworthy? | Savory Sausage 440g |
| B101 | Bought a number of times, engaged in the comments do not know how to write, the flavor is very good, young and old alike, simple steamed is a plate of wine dishes, complex, steamed rice, fried vegetables can also be, depending on your level ~ | Savory Sausage 440g |
| B102 | Has been countless times back to buy the family loves to eat this brand of spicy flavor children than the general spicy sausage meat lean taste authentic fried dishes or do spicy meat rice are very good And in the epidemic period Jingdong logistics is really powerful the next day can be received This is also I have been more like to buy Jingdong self-supporting products believe that the quality of the Jingdong and after-sales service Thank you courier brother in the special period is still stick to their posts to provide service for everyone! Support Jingdong | Spicy flavor 440g |
| B103 | Sausage fat and lean ratio is very good, income accounted for 70%, eat up should not be particularly greasy, the taste of this sausage is really I have eaten sausage inside the best flavor sausage. Meizhou Dongpo's restaurant inside this sausage is with the sliced directly after the table, the price in fifty or sixty dollars. Jingdong on the price is simply wholesale price, and affordable quality and good, love Jingdong. | Savory 440g |
| B104 | Have eaten the best salami, eat really found the new world, really delicious, steamed rice standard with salami, who eat who knows, a taste is delicious, normal price to buy very expensive, can only wait for the event, really cheap. | Sausage Savory 440g |
| B105 | The first time to buy this brand of sausage on the Jingdong, I do not know how to taste, the courier brother is very powerful, delivery is very timely, a time to buy three packages, the original flavor of two packages, spicy flavor of a packet, the first taste taste taste fresh. Packaging can | Spicy flavor 440g |
| B106 | The first time to buy this brand of sausage on Jingdong, first bought three packages to try, taste good or not, the courier boy is very powerful, delivery is very timely, but the production date is January 17th shelf life is one year. | Savory 440g |
| B107 | Frozen delivery, logistics is also very fast. Activity price for the first time to try to see the mind to buy three packages, see before buying is authentic Sichuan flavor, used to eat Cantonese sausage more afraid of family members are not accustomed to taste, worry about redundant. Immediately after receiving the goods steamed two, sliced without adding any seasoning to eat a piece, slightly salty, very fragrant, chewy, with the same taste as the previous food in the Dongbo restaurant. More delicious than Cantonese, after a careful look at the ingredient list, there is no additives and starch and so on, sausage or pig intestines, very rare, a lot of big brands of Cantonese have additives, sausage with not pig intestines but collagen coat. I hope that businessmen continue to maintain the traditional process of production, for diners in other places to provide quality food. After eating continue to repurchase. | Savory Sausage 440g |
| B108 | Already N many times to buy this product, big brand quality is guaranteed, although slightly more fat, but after steaming out of the slice, taste very, very fragrant! , a few slices less at a time, just to satisfy the appetite! The date of production is also relatively fresh, at the end of March this year. In addition, to share a little secret, I now buy chilled food, only buy Beijing has a warehouse, where the field to Beijing, I do not buy. Because, express cold chain transportation almost all the courier companies are not qualified! Summer is coming, this is very important, otherwise you will receive spoiled products! This time, Jingdong Express did a great job, because the goods were in the Beijing warehouse, and the sausages were still hard when they arrived home, which was great! Kudos! | Savory 440g |
| B109 | Shipping was fast, it was still frozen and not melted when it was delivered, it was packed in a foam box with several bags of ice packs, so it was still insulated this aspect is still possible. In addition, he was vacuum-packed, and no inflation, satisfied! | Spicy Sausage 440g |
| B110 | I've been buying Meizhou Dongpo sausages and they are of good quality. Especially the original flavor, not spicy, the texture is very good to eat. Jingdong self-supporting resumed delivery, yesterday's order was delivered this morning, Jingdong, as always, fast delivery, next time. | Savory Sausage 440g |

| serial number | Word-of-mouth text (fancy no-cut cake C) | offerings |
| --- | --- | --- |
| C1 | This red date pumpkin cake is very well made, tiny little bites, one bite at a time, very easy to eat and very well fermented. The texture is delicate and smooth, and the sweetness is just right, not very sweet. The material is very fresh and the workmanship is very delicate. Good packaging, affordable. This can be this assured purchase. | Pumpkin Cake with Red Dates 300g*3bags |
| C2 | With the corn flavor kitten scratch together to buy, black rice flavor is very strong, sweet and delicious, you can eat several, when the breakfast with milk is very suitable, recommended to buy, and will continue to buy, I hope to often engage in activities it. | Moe's Cat Claw Cake Black Rice Flavor 1000g |
| C3 | So cute and cuddly looking, really cute and cuddly, each one is a little cat paw. Bought two flavors at the same time, one is corn, this one is black rice, each has its own flavor, all delicious. Thawed a night in advance, the water boils and steams for a minute or two is enough, soft and fluffy, the texture is particularly good. | Moe's Cat Claw Cake Black Rice Flavor 1000g |
| C4 | So say it, it thief delicious, and I think the children certainly love to eat, inside did not add too much sugar, feel but is sweet, is fragrant sticky, soft, and in the morning when the water boiled, he was basically on the hot, very comfortable, if you want a cup of soya bean milk then it's even better, hey hey hey! | Pumpkin Cake with Red Dates 300g*3bags |
| C5 | The taste is very good, the child likes very much, has been seriously eating, can not wait to finish a plate of pumpkin pie, soft and sweet, color shape is very appetizing look, has bought two times, after eating will also buy back! | Pumpkin Cake with Red Dates 300g*2bags |
| C6 | Perfectly packaged, the courier took it out of the ice pack and delivered it to your door, very thoughtful, no need to worry about frozen food melting away and spoiling in the heat. In the morning, put it in the pot to steam for a few minutes and it's done, the texture is good, delicious! | Pumpkin Cake with Red Dates 300g*2bags |
| C7 | I have not eaten this before to buy back to steam, the taste is really good, sweet and soft and delicious, too delicious, the children also like to eat, when the breakfast is very good, good digestion, delicious flavor, very very very good good good good good good good | Pumpkin Cake with Red Dates 300g*2bags |
| C8 | This cat's paw cake is super cute, one by one small, cute and cuddly, with a particularly festive shape. Thaw it one night in advance, so that you can greatly reduce the steaming time and maximize the original flavor. Personal experience, thawed, water can be boiled after a minute or two. The corn flavor is very strong, soft but also Q Q Q Q, very popular with the family. | Moe's Cat Claw Cake Corn Flavor 1000g |
| C9 | Double flavor lactic acid bacteria roughage cake, cake is very delicious and soft, a little sweet, a large head, when the main food to eat is also OK, the top and bottom of the two layers, the taste is slightly different, cut into the shape of the stars, especially good, very satisfied. Jingdong shopping is very convenient and fast, the key is to deliver a good experience. It is also very convenient to return and exchange anything. Home food and drink things, but also with the baby's things, basically are Jingdong on the shopping. Hahahahahahahahaha | Double Flavor Lactobacillus Rough Cake 510g*3 bags |
| C10 | Logistics sent or hard, not melted at all, not steamed is a strong corn flavor, eat sweet sticky, and not excessively sweet, suitable for the elderly and children, the next time will buy. | Moe's Cat Claw Cake Corn Flavor 1000g |
| C11 | This flavor and the other flavor is very delicious, just a few days let me eat, mainly because they are both I have eaten similar products before, so that I know it is delicious, not so greasy to eat, but one thing is to wear gloves as much as possible to take it, because it's tray is full of oil! | White Sugar Osmanthus Cake Flower 300g*3bags |
| C12 | This pumpkin cake is very well done, a small one, one bite, no need to use a knife, cut very convenient, fermentation is very good, the texture is delicate and smooth, a little like Shunde's Lunjiao cake, accidentally eaten on a plate. | Pumpkin Cake with Red Dates 300g*2bags |
| C13 | It's great very good, the first delivery, the texture is soft and sticky, the sweetness is just right, not very sweet, gently stared in the microwave, a few minutes to eat, the material is very fresh, praise praise praise praise | Pumpkin Cake with Red Dates 300g*2bags |
| C14 | Good value for money, worth buying. Ordered on the same day and delivered the next day, fast logistics. The delivery was timely, and the logistician was very prompt with the delivery. The price is relatively favorable, after using the coupon, it is very cost-effective. The flavor is very good, is familiar with their favorite flavor, after the first taste, I like it. Purchased many times and will continue to support. | Moe's Cat Claw Cake Corn Flavor 1000g |
| C15 | Thousand flavors central kitchen pastries almost contracted my family's breakfast. Bought a variety of styles, every morning to change the pattern, not only to save trouble, but also delicious and nutritious, the family is very favorite. This is the first time to buy the cat's claw, baby like? | Moe's Cat Claw Cake Corn Flavor 1000g |
| C16 | The elderly at home like this cake very much, steamed out fluffy fluffy, but also very fragrant, through a strong corn flavor. Lactobacillus fermentation, healthy stomach and intestines to help digestion, very suitable for the elderly to eat. Face value is also super high, double layer of color, look at the appetite. | Double Flavor Lactobacillus Rough Cake 510g*3 bags |
| C17 | Good packaging, fast logistics, affordable, much cheaper than the supermarket cost-effective very high? The price is very high, you can rest assured to buy? The price is very high, you can rest assured to buy? , worth buying? Really super like, the quality is very good, consistent with the seller's description! | Pumpkin Cake with Red Dates 300g*3bags |
| C18 | I've received it! Very tasty! I will always buy this brand of products! I hope to always adhere to the quality of fine products, very tasty, I bought myself to taste, Mother's Day to my mom and boyfriend's mom also bought, both were touched hahaha, the words written on the back of the box is too good, I myself read all the moving, the writing praise! Too awesome! Received the goods and tasted it, it is really expensive, really delicious. | Moe's Cat Claw Cake Corn Flavor 1000g |
| C19 | I like cinnamon cake very much, this time I found that there is, without hesitation, I bought two bags, the result is very delicious, a good breakfast. Will be back for more often in the future. | White Sugar Osmanthus Cake Flower 300g*2bags |
| C20 | Health food, a piece of two flavors, also I like the soft and sticky kind of feeling, but unfortunately too sweet. Not as good as the cat scratch cake. Jingdong is really good, let me keep changing breakfast varieties, so happy (● °u ° ●)? "I'm so happy. | Double Flavor Lactobacillus Rough Cake 510g*3 bags |
| C21 | Has been purchased many times, this time the inventory is only one package left, baby said very want to eat rice cake, immediately after receipt of the pot steamed, baby very much like to eat, not very sweet flavor is also fragrant, the arrival of the goods will come back to buy. | Pumpkin Cake with Red Dates 300g*2bags |
| C22 | The first time I bought this cake, looked very tasty look, looked at the ingredients list, as if not really pumpkin to do, added pumpkin flavored high-tech it, tasted and then come back to the review. | Pumpkin Cake with Red Dates 300g*2bags |
| C23 | A very delicious hair cake, fluffy and sweet, slightly steamed according to the instructions can be eaten. Adults and children are more like delivery, the speed is particularly fast, when received is well-packed, need to be quick-frozen preservation. That intestines can only be kept for a short time, eat as soon as possible. | Pumpkin Cake with Red Dates 300g*2bags |
| C24 | Eaten a few times, and finally found that it is still cooled after eating is the best texture, the best flavor ......  The price is still particularly suitable if there are activities, cost-effective super high ...... | Double Flavor Lactobacillus Rough Cake 510g*3 bags |
| C25 | Very favorite snacks, the workforce is very suitable, put a few pieces in the steamer in the morning, wash between a few minutes to heat up, very convenient.  Very sweet flavor, often repurchase, taste is very good. | Pumpkin Cake with Red Dates 300g*2bags |
| C26 | Logistics is quick and easy, packaging is intact. It is already a multiple purchase, arriving at home still in a frozen state, the logistics did a good job, delivery, very good service. Affordable price, a must for breakfast. | Pumpkin Cake with Red Dates 300g*3bags |
| C27 | Fresh fragrance, there are red dates fragrance pumpkin fragrance, soft, a little sticky, Q bullet, children like to eat, and not support the stomach, easy to steam, high fire for thirteen minutes, rest assured to eat, convenient! Jingdong self-supporting, fast delivery, buy and eat with confidence! | Pumpkin Cake with Red Dates 300g*3bags |
| C28 | Jingdong self-supporting, fast delivery, this do breakfast well, water steam about ten minutes on the good, taste some sweet overdose, but also many times to buy, figure breakfast cheap and save some time, than do it yourself can save time! | Pumpkin Cake with Red Dates 300g*2bags |
| C29 | Although the double rice cake is written as coarse grains, but it tastes very delicate, and there is no lactobacillus flavor, it is just a normal sweet cake, or a very large one, eat two in the morning will be very full! | Double Flavor Lactobacillus Rough Cake 510g*3 bags |
| C30 | The shape is very cute and the cat's paws are adorable! Small and compact one, the flavor is also good, soft and sticky, the one I like. Not very sweet, eat will not be greasy, can safely eat three, four. | Moe's Cat Claw Cake Corn Flavor 1000g |
| C31 | Children and adults like to eat this, the Beijing East platform to buy this to do breakfast is good, the packaging is complete, the logistics and delivery is also okay, for breakfast worried about parents can pay attention to the | White Sugar Osmanthus Cake Flower 300g*3bags |
| C32 | Logistics is fast and convenient, the packaging is intact. It is already a multiple purchase, and it still remains frozen when it arrives at home, and the logistics are done in place, delivered to the home, and Fuzhou deserves some praise. Affordable price, breakfast essential. | White Sugar Osmanthus Cake Flower 300g*3bags |
| C33 | The flavor is very good, the price is also cheap, but the additives are really much, you can look at the picture, do not dare to give children more food, you can occasionally buy it. Jingdong delivery, received when still ice, overall can it | Double Flavor Lactobacillus Rough Cake 510g*3 bags |
| C34 | I bought a variety of them at one time, they are all frozen food, very suitable for breakfast, it is very convenient to get up in the morning and put them in the pot, and then you can take them out to eat after washing up. The flavor is also quite good | White Sugar Osmanthus Cake Flower 300g*2bags |
| C35 | The osmanthus cake is very sweet, with a light osmanthus fragrance, it should be the embryo made of glutinous rice flour and put the petals of osmanthus flowers.  After steaming the dim sum can feel a layer of oil on the surface, I do not know what kind of oil.  In addition, the instructions say to steam for thirteen minutes, in fact, steam for seven or eight minutes, the rest with residual heat can be.  Packaging is frozen transportation, and ice packs, very careful and thoughtful. | White Sugar Osmanthus Cake Flower 300g*3bags |
| C36 | All of the family's breakfast is bought by the Jingdong family, delivery and fast, inexpensive, convenient. All of the family's breakfast is bought by the Jingdong family, delivery and fast, inexpensive, convenient. | White Sugar Osmanthus Cake Flower 300g*3bags |
| C37 | Every day for the breakfast to do what worry, thousand taste central kitchen to solve a lot of difficulties, variety, good taste, very delicious and soft and a little sweet, a big head, divided into two layers, the flavor is slightly different, made into a star-like, very attractive. | Double Flavor Lactobacillus Rough Cake 510g*3 bags |
| C38 | Babies love to eat, very soft and easy to steam on the good, steamed with rice flavor. The entrance is soft and sticky, and adults also like to eat very much. Jingdong Logistics Frozen is very powerful, order today, tomorrow at noon can receive goods. Recommended for purchase | Pumpkin Cake with Red Dates 300g*3bags |
| C39 | Have not eaten, bought for the child as a breakfast, I hope it is delicious, the price is quite affordable, Jingdong logistics is very fast the next day, after-sales service is also very good, easy to buy, very assured, good next time to buy | White Sugar Osmanthus Cake Flower 300g*2bags |
| C40 | The taste is particularly good, soft and sweet, and I intend to repurchase in a while. Bought a lot of bags, convenient quick food, lazy people refrigerator must have a good thing. At night with a rice cooker reservation, the next day you can eat. | Pumpkin Cake with Red Dates 300g*2bags |
| C41 | Red dates pumpkin cake, slightly sweet, cut a small each piece, suitable for the elderly and children, taste good, soft, like, especially satisfied, looking forward to this brand in the future out of more delicious pastries. | Pumpkin Cake with Red Dates 300g*3bags |
| C42 | White sugar cinnamon cake, a very soft snack, suitable for the elderly and children, cut a very small piece, especially good, very good, not particularly sweet, eat as a staple food is fine, looking forward to the future out of more delicious snacks. | White Sugar Osmanthus Cake Flower 300g*3bags |
| C43 | Thousands of flavors central kitchen, only for catering, chef's choice. Red dates and pumpkin cake flavor is not bad, I have bought this red dates and pumpkin cake many times before, a small bag is enough for 3-4 people to eat, and then with some milk, very nice! | Pumpkin Cake with Red Dates 300g*3bags |
| C44 | Tasty and inexpensive, affordable, eat and buy. Outside the foam box, there are several ice packs, packaging and transportation are very assured, have eaten a thousand flavor central kitchen sesame ball is very delicious, I believe that this should not be wrong, before I have been buying the Sanquan, compared to a slightly more expensive, feel or thousand flavor of the right. | White Sugar Osmanthus Cake Flower 300g*2bags |
| C45 | The osmanthus cake was a gift, I had thought that the gift was not very good, but when I ate it, I found that the business is still quite conscientious, the osmanthus cake is very tasty, sticky and soft, and it tastes faintly sweet, but it's just right for people like me who don't love sweets! | White Sugar Osmanthus Cake Flower 300g*2bags |
| C46 | Already finished eating a bag, finished evaluation, taste is not bad, the morning with the eggs together in the egg steamer to steam a little bit to eat up very convenient, change the kind of buy some can do breakfast things to eat eat. | Double Flavor Lactobacillus Rough Cake 510g*3 bags |
| C47 | The brand is still good, the packaging is also very good, Jingdong is very fast, usually eat roughage is not too much, or to develop such a habit, this product is directly steamed, so it is very convenient to use. | Pumpkin Cake with Red Dates 300g*3bags |
| C48 | Baby received, the outer packaging is intact. The first time to buy this cake,, look up red dates pumpkin cake, looks good, now temporarily not eat, very much looking forward to, expect not to be disappointed. Jingdong self-supporting order, received in the afternoon, courier boy good service attitude, very satisfied, five praise! | Pumpkin Cake with Red Dates 300g*2bags |
| C49 | Good packaging, fast delivery, Jingdong shopping is convenient. Children quite like to eat cinnamon cake, buy some more to eat for breakfast, it is very convenient to heat up. I hope to do more activities ah! Eat up and repurchase ~ | White Sugar Osmanthus Cake Flower 300g*2bags |
| C50 | Really delicious ????  Great taste ????  Wife and son are all praise ????  Must give praise to the almighty Jingdong ???? | Pumpkin Cake with Red Dates 300g*2bags |
| C51 | Home often prepared semi-finished products, fast food, the operation is very simple, this cake cake steamed a few minutes on the good, soft and sticky very tasty, the price of the Jingdong cheaper than the entity store a lot, so stock up a little more | White Sugar Osmanthus Cake Flower 300g*3bags |
| C52 | The quality of the product is reliable and trustworthy, the thing is still good, can meet the needs of most people, the packaging is also very good, there is no damage, and the delivery speed of the Jingdong is still very commendable, very fast. | White Sugar Osmanthus Cake Flower 300g*2bags |
| C53 | Bought a variety of, when the breakfast to eat especially convenient, get up and steam, wash up just go to eat, do not have to worry about home children breakfast. Jingdong self-supporting, with a freezer box, a little bit did not melt. Eat and then buy back | Pumpkin Cake with Red Dates 300g*2bags |
| C54 | Children eating difficulties, bought jujube pumpkin cake for a change of taste, very fluffy, not very sweet, very tasty, adults love to eat, and Jingdong fresh delivery is very professional, freshness is also in place, to the home at all not melt. | Pumpkin Cake with Red Dates 300g*2bags |
| C55 | Daughter-in-law said that this cinnamon cake is better than the breakfast store, sweeter than the store, and there are a lot of cinnamon, it is easy to do, and catching up with the promotional activities is especially cheap, very suitable, after eating back to buy! | White Sugar Osmanthus Cake Flower 300g*2bags |
| C56 | Logistics speed is particularly fast, today's order the next day to, open the outer packaging is tight, no damage, on the pot steamed half a day especially delicious, flavor bar, the solution to the problem of cooking in the morning, especially convenient! | Pumpkin Cake with Red Dates 300g*2bags |
| C57 | New Year's time to buy, eaten before coming to evaluate, this date pumpkin cake taste great, very much in line with my taste, light date flavor, sweet and soft in the mouth, the baby is also very much like to eat, will buy again. | Pumpkin Cake with Red Dates 300g*3bags |
| C58 | Golden rice cake is a little sweet, for the baby to eat breakfast in the morning, or very good, healthy and delicious, Jingdong express delivery is very good, the whole cold chain foam box to the home, very praise, very praise, believe in Jingdong self-production | Pumpkin Cake with Red Dates 300g*2bags |
| C59 | Baby love cinnamon cake, sweet, very delicious, affordable, very convenient, hot in the morning can eat, suitable for working people, very small piece, get 3 pieces in the morning, the baby is very happy to eat. | White Sugar Osmanthus Cake Flower 300g*3bags |
| C60 | Family children especially like to eat rice cakes, this is also a family we often buy ou, texture and so on are very good, soft and sticky very tasty, adults and children are very like hahaha. Really good it. | Pumpkin Cake with Red Dates 300g*3bags |
| C61 | Quite good, the flavor is very good, compared to the general hair cake has a cinnamon flavor, steamed to eat the flavor is very good, can be directly eaten as a staple food, so after eating will come back, great! | White Sugar Osmanthus Cake Flower 300g*3bags |
| C62 | This adults and children can eat, sweet mouth, do not want to eat sweet may not like it too much, I do like to eat this, the child can also eat, the texture is good, every morning steamed to eat, very can. | Pumpkin Cake with Red Dates 300g*2bags |
| C63 | This cut cake purchased for the second time, simple and good to do, slightly sweet and not greasy, nice plate, children like to eat, New Year's Eve on the table with a festive atmosphere. Delivered to the home on the same day, Jingdong logistics is indeed too powerful. | Pumpkin Cake with Red Dates 300g*2bags |
| C64 | One inside three bags, the taste is quite good, a piece of a piece of separation, want to eat to take a piece out, the children also quite like to eat, do breakfast is very good, very convenient, the taste is very good, get a good soft hoo-hoo, very great! | Pumpkin Cake with Red Dates 300g*3bags |
| C65 | Very, very good, very good ????? The steam is really too fragrant, soft and sticky, very entrance, chewy, a bite can eat several, the key is that my children also like, hahaha! | White Sugar Osmanthus Cake Flower 300g*2bags |
| C66 | It's very, very, very, very good, soft and sticky, very Q-bomb, easy to make, our whole family loves it, especially my kids and me, hahahahahaha? It's worth recommending! | Pumpkin Cake with Red Dates 300g*2bags |
| C67 | Now buy New Year's Eve, Jingdong is the most reliable. Whether it's quality or logistics, it's all bang on, it's really the industry's bearer. The courier is particularly responsible, special period to the elevator, especially good attitude. The first day under the order the next day to, ask who else can do! | White Sugar Osmanthus Cake Flower 300g*2bags |
| C68 | Point takeaway when ordered, feel quite delicious, the child also loves to eat, and then saw in the Jingdong, decisive order, after receiving the feeling is good, after steaming is very tasty, good, convenient and hygienic, the price is right, logistics is also very fast, the overall is very good! | Pumpkin Cake with Red Dates 300g*2bags |
| C69 | The kids love it. It's been a long time since I bought it. Today there is an event. I bought a big bag. It's a great deal. Hopefully, there will be more favorable activities. The Beijing East Shopping Center is trustworthy! | Pumpkin Cake with Red Dates 300g*3bags |
| C70 | The big brand is trustworthy! The appearance of a beautiful atmosphere upscale, well-chosen materials, fine workmanship, exquisite materials, delicate taste! The value of the color is very high a variety of fillings to choose from. Good service attitude, logistics speed, will continue to patronize! | Pumpkin Cake with Red Dates 300g*2bags |
| C71 | Daily good helper, simple steam is good, good, good, very recommended, do food at home, at least the staple food to solve the problem, the flavor is good, easy to do, recommend that we all come to taste, haha, like Jingdong | White Sugar Osmanthus Cake Flower 300g*2bags |
| C72 | It's not bad. Steam a ten minutes can eat, especially convenient. The texture is soft and slightly sweet, but the cinnamon flavor is rather weak, slightly strengthened may be better. I hope the business more of this kind of prepared food | White Sugar Osmanthus Cake Flower 300g*3bags |
| C73 | My son loves this pumpkin cake more, it's soft and has a little bit of a pudding like texture. Anyway, every kind of instant pasta in his house is good. It's slightly sweet and not greasy. | Pumpkin Cake with Red Dates 300g*2bags |
| C74 | Red dates pumpkin cake flavor is not bad, do for breakfast convenient and quick, the texture of soft and sweet, pumpkin and dates taste great, rushed to buy two packages of activities at the right price. Breakfast essential food it ！！！！ | Pumpkin Cake with Red Dates 300g*2bags |
| C75 | Very convenient, the taste is also good, is my favorite pumpkin, jujube, after getting up in the morning, the first steam on, you can go to wash, two without delay, is this Jingdong positive feedback to get Beijing beans need to write 60 words, it is really not necessary, which has so many insights, good, not good, bad, not good chanting | Pumpkin Cake with Red Dates 300g*2bags |
| C76 | very good cinnamon sugar cake a want to do it yourself, and afraid of trouble this buy to eat eat very good, is not a lot of portions, a family to eat a may be a little less a person to eat one is about the same, I hope that there has been a discount will continue to come buy | White Sugar Osmanthus Cake Flower 300g*3bags |
| C77 | Received only to find that the portion is quite full, eat up, crispy catalyst, mixed with dates it, eat up will not be greasy, the more you eat the more you want to eat. The shape is beautiful, the date is new, like the pro can get it oh. | Pumpkin Cake with Red Dates 300g*2bags |
| C78 | This person taste or quite good, buy over to eat breakfast, and then also more fill it, children prefer adults to steam a steam can be eaten, and then the amount of healing is not large, and then he in fact, if he becomes that rice ball, it is a very small piece of it, so the breakfast then one time more than one time to win a bag is a minimum of it I think it's not quite good | White Sugar Osmanthus Cake Flower 300g*2bags |
| C79 | Eaten before I want to get up to evaluate, smooth texture is not sweet and greasy, on the pot steam a few minutes to eat, breakfast afternoon snacks are a good choice, the family adults and children like to eat, the date is fresh, Jingdong delivery speed. | Pumpkin Cake with Red Dates 300g*2bags |
| C80 | I love pumpkin and dates, the flavors of these two together should be a perfect match! It's a good choice for breakfast and snacks, fluffy and sweet is my favorite food! | Pumpkin Cake with Red Dates 300g*2bags |
| C81 | I've purchased this hair cake several times from BOE. It tastes good and is easy and quick to eat for breakfast. This time did not see clearly the activities of the Jingdong buy one get one free. Received the goods some regret, forgetting that itself is 2 generations of a package, which all of a sudden 8 generations do not know whether to eat it all. And the production date is 22 years in October has been 4 months. Try to eat it is not a waste. But the Jingdong activity price is strong recommend everyone to buy. | White Sugar Osmanthus Cake Flower 300g*2bags |
| C82 | The child 15 months fast, every day is not to eat noodles or dumplings a little tired of eating, to the child to buy as a breakfast to eat, steamed once quite soft, a little sweet taste quite delicious. Jingdong logistics yyds | Pumpkin Cake with Red Dates 300g*3bags |
| C83 | Express speed is still very fast, the next day was delivered, on the spot unpacking, fresh packaging is quite good, buy a little late, the price is a little expensive, but during the New Year or like to eat some steamed cake, eat the feeling is quite tasty. | Pumpkin Cake with Red Dates 300g*3bags |
| C84 | Jingdong self-supporting, fast delivery, buy to do breakfast, this is a white cake, the flavor is not bad, is sweet, breakfast is also very convenient, steaming about ten minutes basically, more suitable for lazy people. | White Sugar Osmanthus Cake Flower 300g*2bags |
| C85 | Jingdong self-supporting, fast delivery, bought to do breakfast, bought a few times, the flavor is not bad, is sweet, breakfast is also very convenient, steamed about ten minutes basically, more suitable for lazy people. | Pumpkin Cake with Red Dates 300g*2bags |
| C86 | Baby received, the outer packaging is intact. Multiple purchases, good taste, sweet and sour very tasty, will continue to buy back. Jingdong order, fast shipping, courier delivery in a timely manner, very satisfied, five praise! | White Sugar Osmanthus Cake Flower 300g*2bags |
| C87 | Thousands of flavors central kitchen red dates pumpkin cake received, have not eaten it, looked good quite good. Jingdong logistics is very fast yesterday order today, the courier logistics packaging is very good, thank you Jingdong. | Pumpkin Cake with Red Dates 300g*2bags |
| C88 | I've already finished this pumpkin pie, so I had to take a little shot of what was available on my desktop that I purchased on Kyodo, which is the only one I trust for this frozen food because it arrives every other day and is still frozen when it arrives, mmmm. | Pumpkin Cake with Red Dates 300g*2bags |
| C89 | The pastries were delicious and chewy. | Pumpkin Cake with Red Dates 300g*3bags |
| C90 | Red dates pumpkin cake is very delicious, eat more roughage body good. The flavor is very sweet, divided and very convenient to eat. Jingdong self-supporting goods quality is very good, courier boy delivery upstairs, service attitude is very good! | Pumpkin Cake with Red Dates 300g*2bags |
| C91 | Thousand flavors central kitchen's thousand flavors of red dates pumpkin cake, fancy no-cut cake, sweet and soft taste, very delicious, good shape, the family likes to eat, do breakfast, color and flavor, the recipe is very nutritious, eat at ease. Jingdong cold chain transportation, received also frozen hard, Jingdong first-class service, next day delivery, eat and then come back to buy. | Pumpkin Cake with Red Dates 300g*2bags |
| C92 | This time I bought a lot of food, all of them are from Chimei Central Kitchen, there are sesame balls, fried dumplings, osmanthus cake, a big box full of them. The logistics is relatively fast, the outer packaging is strong, no damage. There was dry ice in the foam insulated box, and it didn't melt at all. Cinnamon cake is semi-finished products, take out a little thawed, heating can be, the operation method is simple, very easy to use. | White Sugar Osmanthus Cake Flower 300g*2bags |
| C93 | Shipped quickly, courier delivery to the home in advance of the appointment basically did not thaw?  First time to buy this cinnamon cake, just arrived to get eaten taste good, next time to wait for completely thawed and then on the pot will probably be better? |  |
| C94 | Home snacks toys skin care products are in the Beijing East to buy, good quality and cheap shipping fast! | Pumpkin Cake with Red Dates 300gX2bag |
| C95 | There are dates and pumpkin, comprehensive nutrition, good flavor, fluffy and soft, very suitable for the elderly and children. And the plate is very beautiful. I will buy it again after I finish these two. And already recommended to other people's children's mothers. | Pumpkin Cake with Red Dates 300gX2bag |
| C96 | Carefully selected, well-packed, also insulated express, low temperature preservation transportation, express very quickly, or trust the Jingdong self-supporting quality assurance, eat up is not bad, the whole family like, whether children or the elderly, as a staple food is very good, the early morning can also be. | Pumpkin Cake with Red Dates 300gX2bag |
| C97 | The first time I bought this white sugar cinnamon cake of its family, I feel that he is quite a look, first give a favorable comment, eat better, then will add a favorable comment on the price is quite cost-effective, compared with the supermarket is very favorable, the Jingdong small brother delivery is very fast. | White Sugar Osmanthus Cake 300gx2bag |
| C98 | The first time I bought taste good, easy to make, give my son as breakfast especially like. | Pumpkin Cake with Red Dates 300gX2bag |
| C99 | I've bought many times, prefer fried dumplings, do not like to sleep, this date pumpkin cake delicious, skin firm, pick up will not be loose, really convenient, convenient lunch, quick breakfast, every event will buy this | Pumpkin Cake with Red Dates 300gX2bag |
| C100 | The baby is delicious, and it's a great deal to put together a single shot. | Pumpkin Cake with Red Dates 300gX2bag |
| C101 | I've bought it a few times, it's oily, sweet but not greasy, very tasty and makes a great breakfast. | Pumpkin Cake with Red Dates 300gX2bag |
| C102 | As always, it is delicious, bought at the time of the event, the price is okay, steamed and fried can be eaten, but personally I prefer to fry, crunchy, very tasty, with a little porridge, perfect! Express beauty service attitude is very good, kudos! | Pumpkin Cake with Red Dates 300gX2bag |
| C103 | Looks like it should be good and easy, love these little homemade pastries! | Pumpkin Cake with Red Dates 300gX2bag |
| C104 | Super tasty, next time continue to buy, breakfast solution, eat a lot of flavor, big brand trustworthy is not the same, I hope that the business to protect food safety, for the general public service, the courier is also super fast, the Jingdong Express, really good! | Pumpkin Cake with Red Dates 300gX2bag |
| C105 | It's beautiful and flavorful, and the tray design is so cute that you buy two kinds of cake, eat half of each, and continue to freeze the rest by putting it together on a plate. | Pumpkin Cake with Red Dates 300gX2bag |
| C106 | This white sugar cinnamon cake is very tasty, often bought to eat early, breakfast with millet porridge or soybean milk is very nutritious, sweet but not greasy, and the description of the exact same, very satisfied, really like it, completely exceeded the expectations, the delivery speed is very fast, the packaging is very careful and strict, the deliveryman has a good attitude, the delivery speed is very fast, very satisfied with a shopping. | White Sugar Osmanthus Cake 300gx2bag |
| C107 | It's really delicious, kids love it...will come back for more when I'm done! | Pumpkin Cake with Red Dates 300gX2bag |
| C108 | Get up in the morning and eat some jujube cake is really very good, children are also very like to eat, soft and sweet very delicious, the pot on the steam steam a few minutes on the good, very convenient and quick, very easy to store! | Pumpkin Cake with Red Dates 300gX2bag |
| C109 | These fresh is very good, a buy bought a whole lot, is used and then evaluate; this pile of activities and just is the Jingdong buy, cheaper than the entity store, cost-effective; Jingdong logistics is also very fast as always, the first day under the single the next day delivery; can be said to be a very good shopping experience, full five-star praise to send, next time there will be the opportunity to come back to buy! | Pumpkin Cake with Red Dates 300gX2bag |
| C110 | Open is cut fan-shaped small pieces, easy to take when eating, good-looking and delicious. The color is also very good, eat sweet and delicious, solve the breakfast problem, eat and buy again. | Pumpkin Cake with Red Dates 300gX2bag |

| serial number | Word-of-mouth text (Dumpling D) | offerings |
| --- | --- | --- |
| D1 | This dumpling portion is very large, there are a lot of them, and then a few meals to finish, the logistics and delivery service is very good, there are ice packs to save are not melted, very good delivery, very satisfied. | Steamed Pork Dumplings with Corn 1kg |
| D2 | Pan-fried dumplings have a golden and attractive appearance, each one is full and complete, and when you bite into it, the skin is crispy and the meat is tender. In terms of texture, the pan-fried dumplings have a thin skin and filling, with a unique flavor that whets the appetite. Particularly awesome? | Steamed and fried pork and cabbage dumplings 1kg |
| D3 | Originally just 628 during the single to buy, never bought this brand before, do not know how good hey, the results found that this steamed dumplings ah especially good, did not try to fry the dumplings, but steamed dumplings are really really good ah very tasty children especially especially love! | Steamed and fried dumplings with mushrooms and three fresh ingredients 1kg |
| D4 | Let's put it this way, I am a fresh graduate of the working class, a month to earn that two money is also quite difficult to survive, buy takeout is very difficult, do it yourself also do not have the time, so it chose it, with a very good, and thieves delicious! | Steamed and fried dumplings with mushrooms and three fresh ingredients 1kg |
| D5 | The taste is very good, the speed is also very fast, purchased many times... steam pot on a steam, the aroma of the nose, frying pan fried, crispy and crisp, are very convenient... family breakfast rations must have the best! | Steamed Pork Dumplings with Shepherd's Purse 1kg |
| D6 | Jingdong discount time to buy very cheap, delivery very quickly the next day to the, always like to buy things on the Jingdong quality assurance this brand is a big brand product quality is very good, I like to recommend everyone to buy! | Steamed Pork Dumplings with Corn 1kg |
| D7 | Thousands of central kitchen only for catering chef's choice mushroom three fresh steamed fried dumplings 1kg (total of 50) steamed dumplings fried dumplings frozen dumplings pot stickers breakfast ingredients, dumplings taste very like, mainly very convenient to do very quickly about 20 minutes after the child has been very like to eat, boiled or steamed or fried dumplings, the taste is very good, especially catching up with the activities of the price of special power, corn pork, chestnut pork, mushroom The price is very favorable, corn pork, chestnut pork, mushrooms, all kinds of fillings have been bought, will buy again! | Steamed Pork Dumplings with Corn 1kg |
| D8 | The family's breakfast has always been dumplings, wontons at home, Jingdong self-supporting products have become the standard, this time to buy a lot of hoarding, slowly eat, this fried dumplings affordable, wrapped thick, with electric pan a few minutes to get, with a little soup on the way, Jingdong Express Godspeed, praise! | Steamed Pork Dumplings with Shepherd's Purse 1kg |
| D9 | This dumplings can be, first put the pan hot, put the oil, and then open the smallest fire slowly frying, people do not go away or will hurt paste, this is a big brand, Jingdong Supermarket goods are definitely worth having, especially suitable for students to do breakfast, advise parents not to take their children to eat roadside stalls for breakfast, the body is the first place! | Steamed and fried dumplings with mushrooms and three fresh ingredients 1kg |
| D10 | Jingdong fresh price is affordable, quality is guaranteed, the whole cold chain transportation, received intact, is now the first choice for families to buy fresh food. Multiple purchases of this steamed dumplings corn pork flavor, the price is much more affordable than the supermarket, the taste is delicious, the family loves to eat. | Steamed Pork Dumplings with Corn 1kg |
| D11 | I cooked it strictly according to the instructions on the package, I personally prefer potstickers, so I cooked it according to the frying method. No need to defrost the dumplings. Oil a pan and fry the dumplings on medium heat with the lid on for 2 minutes, until the bottom of the dumplings are slightly golden brown. Then add water to submerge the bottom of the dumplings, cover and fry until the water dries up, about 20 minutes, now this time of the year for fear of undercooked food to eat bad stomach can not be medical care, so more time to burn some. The final texture is still good, is imagined the feeling of fried dumplings, outside to buy fried dumplings because of the addition of more oil, so more crispy, and do it yourself fried dumplings more healthy. | Steamed and fried pork and cabbage dumplings 1kg |
| D12 | This dumpling is very good, first put the pan hot and then put the oil, open a small fire and slowly fry, this skin will never break. Student breakfast is particularly suitable, do not go outside the roadside stalls for children to eat garbage breakfast, this is a big brand, Jingdong supermarket, absolutely assured! | Steamed and fried pork and cabbage dumplings 1kg |
| D13 | Thousands of central kitchen only for catering chef's choice mushroom three fresh steamed fried dumplings 1kg (total of 50) steamed dumplings fried dumplings frozen dumplings pot stickers Breakfast ingredients, dumplings taste like, mainly very convenient to do it quickly about 20 minutes after the child has been very like to eat, boiled or steamed or fried dumplings, the taste is very good, especially catching up with the activities of the price of the special force, corn pork, caper pork, mushroom The price is very favorable, corn pork, chestnut pork, mushrooms, all kinds of fillings have been bought, and will buy again! | Steamed and fried dumplings with mushrooms and three fresh ingredients 1kg |
| D14 | Thousand Taste Central Kitchen Only for catering Chef's Choice Pork and Cabbage Steamed and Fry Dumplings 1kg (Total 50) Steamed Dumplings Fry Dumplings Instant Frozen Dumplings Potpourri Breakfast Ingredients, bought two kinds of stuffed steamed dumplings, packaged well, frozen well, each with their own flavors, it's very convenient and very tasty, not bad, not bad ！！！！ | Steamed and fried pork and cabbage dumplings 1kg |
| D15 | Breakfast ingredients, bought two kinds of filling steamed dumplings, packed very well, frozen well, each with its own flavor, very convenient and delicious, good, good! This brand to eat are quite good, always buy, will buy back series, each time is to buy a lot, very good. Next time will come again! Received not frozen, activity price to buy, affordable! Adults and children like to eat, as breakfast, very convenient, microwave heating can be. | Steamed Pork Dumplings with Corn 1kg |
| D16 | Jingdong logistics is still very fast, this kind of frozen will keep warm very well, will not let it go bad, this is already a number of times to buy, each time the several kinds of fillings all over again, the corn personally think it is very good | Steamed and fried pork and cabbage dumplings 1kg |
| D17 | Very good, very good value, cost-effective. This time there is an event, just bought, is a little buy more, the refrigerator have can not put. Fry a few every day, breakfast can be solved, very good. Next time will buy back. | Steamed Pork Dumplings with Corn 1kg |
| D18 | Things are good, there are discounts, you can rest assured to save money to buy, do not hesitate to be the ideal product? , the quality is good, the logistics is also very fast, worthy of praise! Baby is really great, personally very much like, friends also say good to come, recommended to buy! | Steamed Pork Dumplings with Corn 1kg |
| D19 | This dumpling is quite delicious, the flavor is suitable for salty and light, the filling does not feel so much high-tech, the dumpling skin is also relatively strong, fried dumplings are also very convenient, very much to my liking, and will be back to buy. | Steamed Pork Dumplings with Corn 1kg |
| D20 | There are several flavors of this fried dumpling, and I like the caper one the most, it is very fresh and has a very good texture, and the logistics is very fast, and I received the goods on the next day. Also refrigerated very well, did not melt at all, Jingdong Logistics is trustworthy. | Steamed Pork Dumplings with Shepherd's Purse 1kg |
| D21 | Bought a few times Aizen home of this dumpling, very like to eat, sometimes steamed, breakfast the whole few with some porridge, especially convenient, often buy things in the Jingdong, delivery speed, service is also good, happen to be the activities can be stocked up on some daily necessities or food, Jingdong things shelf life are good, of course, try to buy self-supporting. | Steamed Pork Dumplings with Corn 1kg |
| D22 | The dumplings taste okay, you can steam them or fry them, put them in the freezer and make a little when you want to eat them, it's still quite convenient. The portion is very large, the date is also very fresh, and the packaging is also OK. | Steamed and fried pork and cabbage dumplings 1kg |
| D23 | Thousand Taste Central Kitchen only for catering Chef's Choice Shepherd's purse pork steamed fried dumplings 1kg (total of 50) steamed dumplings fried dumplings frozen dumplings pot stickers Breakfast ingredients, put in the pan on low heat and fry for about 10 minutes, golden brown on both sides, according to their own preferences with side dishes or eat directly, the taste of crispy and delicious, when the breakfast is very convenient, a few minutes to get it done, small children go to school does not delay time, and children like to eat, add an egg nutrition can also be! can be, green onion flavor, not greasy, do not need to thaw, convenient and quick! | Steamed Pork Dumplings with Shepherd's Purse 1kg |
| D24 | This dumplings have not been bought before, now there is a promotion is very strong, randomly buy a whole lot, this dumplings thawed, after putting a long time will not stick together, now the weather is hot, and do not think about frying, is boiled, the taste is also good! | Steamed Pork Dumplings with Corn 1kg |
| D25 | Thousand flavors central kitchen pork and cabbage steamed fried dumplings has been the second purchase, a large bag of 1Kg, each dumpling is quite large, fried up with oil to eat, crispy skin, bite open the inside of the full of pork flavor. It is very delicious. Production date February 24, 2023, shelf life frozen 12 months. | Steamed and fried pork and cabbage dumplings 1kg |
| D26 | Thousand Taste Central Kitchen Corn and Pork Steamed Pan Fried Dumplings are delicious, thin skin and large filling, can be steamed and fried, nutritious and delicious, thin and translucent skin, strong taste, selected wheat flour, high-quality pork, sweet corn kernels preparation, first-class flavor, fresh and delicious, excellent taste, Jingdong fresh, quality is guaranteed, the whole process of cold-chain transportation, the date of production is approaching, the portion is sufficient, the packaging is beautifully tight and tight, the outer package is intact, and the delivery speed is fast. | Steamed Pork Dumplings with Corn 1kg |
| D27 | The third time to buy a thousand flavors of home, the most favorite to eat this steamed fried dumplings, used to buy pork cabbage, this time to buy a taste of corn, often in the Jingdong to buy things, delivery speed, service is also good, happen to be the activities can be stocked up on some of the daily necessities or food, the Jingdong things shelf life are good, of course, as far as possible to buy the self-supporting. | Steamed and fried pork and cabbage dumplings 1kg |
| D28 | Deep frying was salty, steaming was better, but the salt would probably be perfect with less salt. | Steamed Pork Dumplings with Corn 1kg |
| D29 | See the store promotional activities, two 15% off, and coupons can be stacked, the price is cheap to get. Received in good condition without damage, the production date can also be, is this year's February. Frozen food is to figure convenient, occasionally lazy cooking on the line. | Steamed Pork Dumplings with Corn 1kg |
| D30 | Although it is the first time to buy this brand of fried dumplings, the packaging is not bright, but whether it is steamed or fried, the taste made is still good, I personally think that the fried is better than the steamed out of the flavor, the aroma is more intense. | Steamed and fried pork and cabbage dumplings 1kg |
| D31 | Have bought once, eaten and gave five-star praise, complimented, immediately chased the order of two, today and received the goods, express time seconds, packaging intact. Last time only praised the quality, today also want to talk about this product - delicious, delicious and labor-saving, put the cake pan cooking, less oil, with the same as doing potstickers dumplings, almost cooked when less water, water dry on the pot. Save time and energy convenient and fast. | Steamed Pork Dumplings with Shepherd's Purse 1kg |
| D32 | Used to feel very good, the quality of goods is good, the delivery is also quite fast, in short, it is very praise overall is still very good, the shopkeeper service attitude is very good, and the quality is also very good, there is no odor Used to feel very good, the quality of goods is good, the delivery is also quite fast, in short it is very praise | Steamed Pork Dumplings with Corn 1kg |
| D33 | The first one I bought was the pork capon one, but neither the little one nor dad liked capon, just myself alone. At that time, I thought this brand was good, thin skin with a lot of filling, the flavor is also good. After that, I bought a few times to buy the corn and pork, and the little one named the corn? The kids asked for the corn? I've bought it many times and recommend it. | Steamed Pork Dumplings with Corn 1kg |
| D34 | The flavor is very good, used for breakfast to eat, simple and quick to heat up, the packaging is perfect, the value is very high, I feel that the value of money! Customer service is also very patient and meticulous, fast delivery logistics, will come back later? | Steamed and fried pork and cabbage dumplings 1kg |
| D35 | Shipping is fast and BOE delivery is guaranteed. This favorite fried dumplings, bought a lot of hoarding during the epidemic. It is easy to do, good-looking and delicious, like the most common cabbage pork, breakfast and lunch can be a, but unfortunately the family can not put too much. | Steamed and fried pork and cabbage dumplings 1kg |
| D36 | I bought a variety of them at once, they are all frozen food, very suitable for breakfast, it is very convenient to get up in the morning and put them in the pot, and then take them out to eat after washing up. The flavor is also quite good. Jingdong Express Delivery **** is good. | Steamed Pork Dumplings with Shepherd's Purse 1kg |
| D37 | Really good, match the picture, there is no difference, really good value for money, good price ah. The quality is very good, the next time will be patronized again! Express is very powerful, praise! Fast shipping cost-effective, give full praise! I buy things used to silently shoot, there is no big problem is not going to ask customer service, of course, this comment. Here I hope that the owner of the store is doing better and better, more repeat customers. Very good shopping experience , compare a number of finally chose this one, and it is very reassuring! Customer service is also very responsible, very | Steamed Pork Dumplings with Corn 1kg |
| D38 | Thousands of central kitchen only for catering Chef's Choice Mushroom Three Fresh Steamed and Fry Dumplings 1kg (total of 50) Steamed dumplings Fry dumplings Frozen dumplings Potstickers , bought to eat as breakfast, steamed in the morning, fried are very convenient, the taste is also very good, long-term hoarding models, the quality of the bar, like to eat this dumplings, hoarding ho do not blink, to buy four packages also send a couple of bags of fish stuffing, are delicious, very cost-effective, ah, will buy again! | Steamed and fried dumplings with mushrooms and three fresh ingredients 1kg |
| D39 | This caper-filled dumplings are very delicious, first of all, the first is a long time to cook or steam will not break the skin, and secondly, the filling is very sufficient, a lot of capers, the flavor is very good, whether it is boiled to eat, steamed, or pan-fried to eat, are very good! | Steamed Pork Dumplings with Shepherd's Purse 1kg |
| D40 | Fast shipping and great value. Steams and cooks well, doesn't stain or rot, tastes great. Steaming for 15 minutes is fine, very good for breakfast. Not too salty, you can add some soy sauce for flavor. All five stars praise! | Steamed and fried pork and cabbage dumplings 1kg |
| D41 | I bought it again and again, the delivery is very fast. 1kg is quite a big bag, the chestnut pork is the family's favorite flavor, but they do not accept the texture of the steamed dumplings, my family just make fried dumplings for breakfast, especially popular! | Steamed Pork Dumplings with Shepherd's Purse 1kg |
| D42 | Very good ingredients are very convenient and healthy not the first time to buy the future will continue to buy especially suitable for people like me who eat irregularly can be quickly realized, eat immediately | Steamed and fried dumplings with mushrooms and three fresh ingredients 1kg |
| D43 | Jingdong delivery logistics fast, ordered last night today at noon to arrive, bought a lot at a time, the refrigerator is full. Put in the home down-to-earth, do not want to cook when you eat these dumplings? Haha convenient and quick. | Steamed Pork Dumplings with Shepherd's Purse 1kg |
| D44 | Shepherd's purse pork dumplings, breakfast is very convenient to eat, big brand, thousand flavor central kitchen quality assurance, dumpling skin long cooking does not break, can also be steamed to eat, of course, the best way to eat or pan-fried, slowly fried over low heat, very tasty! | Steamed Pork Dumplings with Shepherd's Purse 1kg |
| D45 | This pan-fried dumpling from Thousand Taste Central Kitchen is very delicious, the skin is not too thick, but it won't break even if it is boiled for a long time, steamed for a long time, or fried for a long time, the filling is sufficient and the taste is very good, it's suitable for breakfast, or to replenish the energy when you are hungry, it's very convenient. | Steamed and fried pork and cabbage dumplings 1kg |
| D46 | The first time I purchased dumplings from Thousand Flavors Central Kitchen, first of all, it is a large portion bag, the price is very sincere, the cost-effective piece is basically difficult to exceed. The more you come to the water fried, according to the cooking instructions on the bag, the taste is great. | Steamed and fried pork and cabbage dumplings 1kg |
| D47 | Six-star praise, one more star is not afraid of your pride, still hesitant friends hurry up, conscientious recommendation, really good, cost-effective and high, the future is also in this buy. In short: satisfied! Satisfaction! Satisfaction! Really super like, very supportive, the quality is very good, and the seller described exactly the same, very satisfied, completely exceeded expectations, the shipping speed is very fast, the packaging is very careful, strict, the logistics company has a very good service attitude, the delivery speed is very fast, very satisfied with a shopping it ~ ~ ~! | Steamed and fried dumplings with mushrooms and three fresh ingredients 1kg |
| D48 | Steamed dumplings repurchase many times, the filling is fresh, breakfast steam can eat, very convenient. Can also be boiled, steamed, fried dumplings, the portion is very full, the filling is also a lot of recommended to buy corn and pork steamed dumplings! | Steamed Pork Dumplings with Corn 1kg |
| D49 | After all, it is a big brand, this dumpling is very good, the head is quite big, the filling is very full, the flavor is also good, the key key is that the dumpling skin will not be so easy to break, can be boiled, can be steamed, can be fried, are very tasty, repurchase many times! | Steamed Pork Dumplings with Shepherd's Purse 1kg |
| D50 | Favorite caper dumplings? You don't usually see this product? It is very tasty steamed and fried? Especially for breakfast, it's really nutritious and easy to make, so I really like it. | Steamed Pork Dumplings with Shepherd's Purse 1kg |
| D51 | The flavor is super yummy super like? How about repurchasing again after your first purchase? Frying, steaming can be? Getting up in the morning is easy and quick to operate, enriching the breakfast varieties enriching the breakfast nutritional value | Steamed and fried dumplings with mushrooms and three fresh ingredients 1kg |
| D52 | Really like it, very fast shipping, fast delivery, very beautiful price, stuff is still good, will continue to repurchase! All five points! Ness Ness ~ very satisfied with a shopping baby, the price is very affordable, highly recommend this, super like! | Steamed and fried dumplings with mushrooms and three fresh ingredients 1kg |
| D53 | This brand of steamed dumplings taste pretty good, the family likes to eat, so if the Jingdong on the activities will buy some more, in the refrigerator inside the stock, want to eat when you can do it at any time, it is very convenient. | Steamed and fried dumplings with mushrooms and three fresh ingredients 1kg |
| D54 | Bought several bags at a time, the last time I bought corn pork, the taste is very good, this time to buy bags of mushroom flavor, delicious will buy more bags. Steam in the morning, wash up just right. No need to go out every day to line up for breakfast! | Steamed and fried dumplings with mushrooms and three fresh ingredients 1kg |
| D55 | The taste is very good ah, salty taste is just right, fried dumplings will not be salty, some brands of dumplings to do fried dumplings will be a little salty, this will not, eat up just right. The meat filling is also very good, fat and thin properly, not greasy, family members eat up very like. The size is also more appropriate, a mouthful of a quickly dry. Next time to buy. | Steamed Pork Dumplings with Shepherd's Purse 1kg |
| D56 | Dumplings are large, stuffed with enough material, pan-fried, fried, deep-fried, barbecued and other ways are particularly good, dumplings are easy to cook, do not break the skin, not stuffed teeth, easy to digest, absorb nutrients, the price is not expensive, very satisfied with the food products | Steamed and fried pork and cabbage dumplings 1kg |
| D57 | Fast shipping, cold chain delivery, well packaged, inexpensive and good value. Very good breakfast, steamed and fried are suitable, good flavor, with soy sauce sesame oil taste better. Worth repurchasing breakfast, all five stars. | Steamed and fried dumplings with mushrooms and three fresh ingredients 1kg |
| D58 | It can be, quite delicious, the child likes to eat, it is very easy to do, see Man like to eat, I am especially happy, I think it is very good, the child as long as I am happy I am happy, I hope that someday he can see what I say | Steamed and fried pork and cabbage dumplings 1kg |
| D59 | I think it's okay, the flavor is very like, the main thing is very convenient to do very quickly about 20 minutes after the child has been very enjoyable to eat, see him like to eat I'm very happy will often come back to buy also will give other friends recommended! | Steamed and fried dumplings with mushrooms and three fresh ingredients 1kg |
| D60 | very convenient or has always loved to eat, every time I do it is very happy, these are happy look, this time to buy a lot of still quite cheap, I guess I can eat for a while is mainly very convenient will continue to buy, will also recommend to other friends | Steamed Pork Dumplings with Corn 1kg |
| D61 | Good, very good, good quality, fast, strong logistics, after-sales service, good quality, will come back in the future, five-star praise, highly recommended, will come back! | Steamed Pork Dumplings with Corn 1kg |
| D62 | It's authentic and not too expensive, it's great to use, it's been working well? Great brand! Trustworthy! The effect is super good! Expensive for a reason! Great item! Affordable price, great quality, very good shopping, next time come back to the genuine store, rest assured that the purchase, customer service attitude is also very OK, the whole shopping process is very smooth! Will come back next time! Likes! | Steamed Pork Dumplings with Corn 1kg |
| D63 | I bought three bags this time, and the activity counts for convenience. Breakfast is very convenient, do not need to panic to do the morning, with milk and eggs nutrition is not bad. The point is that the children at home like to eat, especially the corn, never get tired of eating! | Steamed and fried dumplings with mushrooms and three fresh ingredients 1kg |
| D64 | It has been a repurchase many times. Kids love it. Breakfast is easy too, with milk? Eggs? Just very? The main thing is convenient, do not have to delay a lot of time to do in the morning, quite save time | Steamed Pork Dumplings with Corn 1kg |
| D65 | Quite good, do breakfast is really inexpensive, Jingdong self-supporting, order in the morning, afternoon delivery, delivery speed. Really too cost-effective, to friends recommend this purchase, a breakfast to eat two a beautiful | Steamed and fried pork and cabbage dumplings 1kg |
| D66 | Quite good, do breakfast is really inexpensive, Jingdong self-supporting, order in the morning, afternoon delivery, delivery speed. Also send dumplings, really too cost-effective, to friends recommend this purchase, a breakfast to eat two a beauty! | Steamed Pork Dumplings with Shepherd's Purse 1kg |
| D67 | At once bought six bags of Chimei Central Kitchen brand pork and corn filled steamed and fried dumplings, a bag of one kilogram, the date of production is very fresh, fine workmanship, Jingdong fresh transportation, safe and secure and fast! Dumplings? The flavor is very good! | Steamed Pork Dumplings with Corn 1kg |
| D68 | Received! Jingdong express transportation, safe and secure fast, fresh transportation, get the hand is very fresh la? Corn stuffed with pork, net content of 1 kg, a bag full of fifty, quick-freezing technology, lock fresh ingredients. | Steamed and fried pork and cabbage dumplings 1kg |
| D69 | Steamed dumplings are very good, have twice repurchased, the skin is very strong, will not break at all, there are different flavors can be replaced, as a breakfast is very suitable, the cost is particularly high. Packaging is very sturdy, delivery is very fast, when it arrives it is still frozen. | Steamed Pork Dumplings with Shepherd's Purse 1kg |
| D70 | The steamed dumplings are great, have repurchased twice, don't break at all, have a variety of flavors of fillings that can be replaced, are exceptionally cost effective, and are extremely suitable for breakfast. Express delivery is also very fast, the packaging is also very strong, when it arrived is still frozen. | Steamed and fried dumplings with mushrooms and three fresh ingredients 1kg |
| D71 | Steamed dumplings are particularly good, has been two repurchase, will not break at all, the filling is also good, as a breakfast is particularly suitable, you can also change the different flavors, cost-effective particularly high. Express especially fast, well packaged. | Steamed Pork Dumplings with Corn 1kg |
| D72 | Jingdong self-supporting products to buy assured, after-sales service is also quite very good, self-supporting products quality is really good, the price is also cheaper than the supermarket to buy, logistics is also very good, are delivered to the home! Dumplings are very tasty, bought several times | Steamed and fried pork and cabbage dumplings 1kg |
| D73 | Corn and pork steamed dumplings, a very large bag, the portion is sufficient, two ways to eat, no need to thaw, eat very convenient, the taste is also very good, corn kernels fragrant, well-packed, delivery is very fast, the Spring Festival every other day! | Steamed Pork Dumplings with Corn 1kg |
| D74 | Jingdong bought, are eaten in the morning, it is very convenient, usually buy a big brand, the taste is very good, used to be about 50% off, now I do not know whether it is the epidemic or New Year's Eve, a good discount is about 75% off. | Steamed Pork Dumplings with Corn 1kg |
| D75 | Always buy this, this time to buy 15 bags, the refrigerator can not put it, eat for many years, or that flavor, this brand filling, thin skin, the ingredients are the best quality, eat very assured! | Steamed and fried dumplings with mushrooms and three fresh ingredients 1kg |
| D76 | This fried dumplings is also too delicious, easy to operate and fast, usually want to eat at any time from the refrigerator to fry a little, the bottom of the crispy flavor inside the material is strong, with the sauce is great, belong to the home of the regular preparation. | Steamed Pork Dumplings with Shepherd's Purse 1kg |
| D77 | It's the second time I bought the steamed and fried dumplings from Thousand Taste Central Kitchen, it's a good deal for the quantity, and the dumplings are good, big and stuffed, they are really tasty, I'll buy them again when I'm done! Steam a pot in the morning, it only takes a few minutes, a breakfast is ready. | Steamed and fried dumplings with mushrooms and three fresh ingredients 1kg |
| D78 | The dumplings are very good, you can try to buy them, whether boiled or steamed or pan-fried, the overall flavor is very good, uh, and the meat filling is also very sufficient. | Steamed Pork Dumplings with Corn 1kg |
| D79 | Pan-fried dumplings are really delicious, the morning or evening do not want to cook, see a child adults like the price is affordable, especially catching up with the activities of the time of the brutal reduction of special power, Jingdong delivery especially fast during the New Year is also very powerful, recommended to buy. | Steamed and fried dumplings with mushrooms and three fresh ingredients 1kg |
| D80 | The steamed dumplings with capers are really good, I've bought them twice, they are not greasy, have a light flavor and are relatively refreshing. It's easy to make, just fry them in a little oil in a pan, add some water and cover the pan with a lid to dry them out. | Steamed Pork Dumplings with Shepherd's Purse 1kg |
| D81 | Things received, very satisfied! Really super good seller, answering questions relentlessly, meticulous and serious, the key is that the quality of the stuff is really good, and the goods are sent super fast, carefully packaged, trustworthy! | Steamed and fried dumplings with mushrooms and three fresh ingredients 1kg |
| D82 | Goods received, especially like, once received, can not wait to open, made potstickers, corn particles clearly visible, less meat, basically satisfied, this shopping experience is good! | Steamed Pork Dumplings with Corn 1kg |
| D83 | Baby especially like, the value of high-quality good, in the look and use, there is a need for friends do not hesitate, and finally wish merchants prosperous business! Received it, very good, very much like, worth buying, (*^__^*) hee hee &hellip; &hellip; | Steamed and fried pork and cabbage dumplings 1kg |
| D84 | Baby is really great, personally very much like, friends also say good to come, recommended to buy worth buying the product, workmanship is very fine, better than imagined, recommended to try! | Steamed and fried dumplings with mushrooms and three fresh ingredients 1kg |
| D85 | Received it and tried it out, it's great, great quality and worth having! Five-star praise! Finally received the goods, very very much like, exquisite and beautiful workmanship, has been praised by many people, really too happy, is already a loyal fan of his family, will continue to buy buy buy! | Steamed and fried pork and cabbage dumplings 1kg |
| D86 | Mustard flavor or can, but corn pork tastes like a strange flavor, in short, do not recommend, but also bought other brands of corn pork, the taste is okay, this brand of corn pork can not, mustard pork flavor is barely passable, well, fried dumplings, in fact, with the dumplings is almost the same, just he wrapped in a different shape, pan-fried out of the effect is still quite good! | Steamed Pork Dumplings with Shepherd's Purse 1kg |
| D87 | Fast delivery, order in the morning courier delivery in the afternoon, from the insulation box out of the dumplings are still frozen, preferential activities buy two get one free, the price is friendly, steaming can be 18 minutes, with dumpling vinegar will be more flavorful. | Steamed Pork Dumplings with Corn 1kg |
| D88 | The dumplings from Thousand Flavors Central Kitchen were a first time purchase. Steamed and fried dumplings with chestnut and pork. I think the ingredients are still quite good. The texture is also quite good. Not too oily and not too salty. Either steamed or fried, there is a simple introduction on the bag. Convenient to operate. | Steamed Pork Dumplings with Shepherd's Purse 1kg |
| D89 | Thousand Taste Central Kitchen, the quality is good, the taste is great, is slightly salty, you can steam ah fry, do dumplings is also good, more salty I eat as dumplings, thin skin and not easy to rot, thawed and will not be sticky, very good! | Steamed and fried pork and cabbage dumplings 1kg |
| D90 | This brand of fried dumplings can be, the child quite like, every day to work no time, come back at night to the baby to get down, it can be dinner, fast food era, everything to save time, the third purchase. | Steamed and fried pork and cabbage dumplings 1kg |
| D91 | Steamed dumplings with mushrooms and three kinds of mushrooms. There are fillings made of mushroom type and pork etc. Overall the flavor is okay and on the light side. It can be steamed or fried. The fried one is recommended for better flavor. It is recommended to try it. | Steamed and fried dumplings with mushrooms and three fresh ingredients 1kg |
| D92 | This dumpling breakfast processing is more convenient, a practice is to steam for fifteen minutes a practice is fried can be, a kind of dumplings a variety of ways to eat, work a more suitable breakfast choice, special good food. | Steamed Pork Dumplings with Shepherd's Purse 1kg |
| D93 | Last time, I bought a bag of corn and pork steamed dumplings, and it was quite tasty. After eating it, I hurriedly bought a few more bags, and this time I bought a bag of mushroom flavor. Wake up every morning to steam, wash up just right, do not have to go out every day to buy breakfast. | Steamed Pork Dumplings with Corn 1kg |
| D94 | One size smaller than the king dumplings from Bipin Kaku, they are similar in flavor, appearance, and fabric, and are perfect for making fried dumplings. Fry them a little, pour half a bowl of starchy water over them, cover and simmer for 7 or 8 minutes, and you've got yourself a breakfast in 10 minutes. | Steamed Pork Dumplings with Shepherd's Purse 1kg |
| D95 | I grew up love to eat fried dumplings ah fried bread ah, the old man of the family used to be at home, now there is no longer, the only way to buy frozen, but so many brands are not suitable for frying, this is just the right time, I have bought two of the flavors that I love to eat, slowly taste! | Steamed Pork Dumplings with Corn 1kg |
| D96 | I haven't eaten this one yet, it's looking at good reviews and good sales, and the dumplings? The shape of the package is just right for me to fry and eat, I like to eat fried dumplings, the previous home-packed is good to fry, this is like home-packed, good! | Steamed Pork Dumplings with Shepherd's Purse 1kg |
| D97 | I've repurchased this dumpling many times? , as a convenient meal is quite a good choice, fried to eat boiled can be eaten it, I'm lazy are boiled to eat. Buy a few bags at a time to hoard do not want to cook slowly eat, is a good choice for lazy people. | Steamed Pork Dumplings with Shepherd's Purse 1kg |
| D98 | The taste is really good, frying is also quite convenient, directly brush a layer of oil, set up on the line, hot with a little water, and then smothered for a while on the finish, the cabbage pork flavor is really good, next time you can buy a few bags more | Steamed and fried pork and cabbage dumplings 1kg |
| D99 | Purchased food in this store for three years, the store honest amount, the price is not expensive flavor is good! This fried dumplings is the first time to buy, have not had time to taste, eaten and then comment. | Steamed Pork Dumplings with Shepherd's Purse 1kg |
| D100 | I've bought their dumplings many times, it's more convenient, whether it's steamed, boiled, cooked or fried. Frozen dumplings, still taste good.  I've bought some of this brand, and everything else, it's okay. | Steamed and fried dumplings with mushrooms and three fresh ingredients 1kg |
| D101 | Arrived very quickly, a bag is very large, 2 pounds, you can steam or make fried dumplings, but the ingredients are not very good, there is margarine, there are a lot of seasoning additives, have not eaten, the arrival is quite fast. The flavor is not yet known. | Steamed and fried dumplings with mushrooms and three fresh ingredients 1kg |
| D102 | This mushroom dumplings from Thousand Taste Central Kitchen are really delicious. The filling is big and flavorful, when you don't want to cook, you can eat it right away, the lazy man's gospel, a bag of more than 50 family is enough to eat, repurchase many times! | Steamed and fried dumplings with mushrooms and three fresh ingredients 1kg |
| D103 | Jingdong logistics is fast The next day it arrived Activity to buy the price is right Cheaper than the supermarket and do not have to go out How fast and good savings This fried dumplings are quite tasty Eat and come back! | Steamed and fried pork and cabbage dumplings 1kg |
| D104 | Thousand Taste Central Kitchen Pork and Cabbage Steamed Pan Fried Dumplings 1kg (Total 50) Steamed Dumplings Pan Fried Dumplings Potstickers Breakfast Ingredients, very tasty. I grew up not eating dumplings, pork cabbage ginger, eat 3 will vomit, but fried dumplings are delicious, with ginger is not afraid, strange, right? | Steamed and fried pork and cabbage dumplings 1kg |
| D105 | Very good, buy 2 bags and get a free bag, cheap, sufficient portion, good quality, date is guaranteed, Jingdong Fresh packaging is good, fast logistics, good service, very satisfied! | Steamed and fried dumplings with mushrooms and three fresh ingredients 1kg |
| D106 | First time I bought this brand of steamed/fried dumplings, one packet is a very sufficient portion, weighing a kilogram. Ordered a couple of packs on offer and got two large packs of fried dumplings free, enough to last a couple of months in the freezer! Very good value for money! Highly recommended! | Steamed Pork Dumplings with Shepherd's Purse 1kg |
| D107 | This brand of dumplings is good quality, details, quality, taste are very good, shipping thief fast, express delivery is also strong, great ~????? Visualize the double eleven activities will be more powerful, want to eat don't miss it! | Steamed Pork Dumplings with Shepherd's Purse 1kg |
| D108 | Delicious, not expensive, affordable, good value, new date, multiple purchases, trustworthy | Steamed Pork Dumplings with Corn 1kg |
| D109 | Good value, affordable, new date, great flavor, tasty, not expensive, multiple purchases, trustworthy | Steamed and fried dumplings with mushrooms and three fresh ingredients 1kg |
| D110 | I'm a little late in evaluating, I've been busy lately, but my son ate it all long ago, it's very good with thin skin and big stuffing. | Steamed Pork Dumplings with Shepherd's Purse 1kg |

| serial number | Word-of-mouth text (Hot pot meatballs E) | offerings |
| --- | --- | --- |
| E1 | This is closer to authentic beef meatballs, I've bought it many times, it's basically a hot pot, it's also good with fried rice and noodles. | 200g Chaoshan Style Beef Balls*1bag |
| E2 | Received, well packaged, very fast logistics, customer service attitude is very positive, questions answered in a timely manner! Overall very satisfied! The quality of the stuff is very good, and the seller described exactly the same, very satisfied The quality of the stuff is very good, and the seller described exactly the same, very satisfied! | 200g Chaoshan Style Beef Balls*1bag |
| E3 | Bought this batch of frozen ingredients, due to ill health can not go to pick up the goods in time, the courier refused to send to the door of the house on the ~ ground, a day after going to pick up the goods found that all the ingredients melted. | 200g Chaoshan Style Beef Balls*1bag |
| E4 | Quality is very good, received the first time to open the goods to see, it is very good, very satisfied with the good value for money, logistics is strong, worth repurchasing cost-effective, very affordable, the use of the feeling is very good, very perfect . ^? ^. | 200g Chaoshan Style Beef Balls*1bag |
| E5 | Very trustworthy brand, quality is guaranteed, the price is also friendly, quite satisfied with the big brand! Trustworthy! The effect is super good! Expensive has the reason! Great item! Very trustworthy brand, quality is guaranteed, the price is still affordable, quite satisfied! | 200g Chaoshan Style Beef Balls*1bag |
| E6 | Shipping was fast, things were well packaged, great price, I'm very happy? and will buy again! | 200g Chaoshan Style Beef Balls*1bag |
| E7 | Extra extra extra extra good, nothing broken and looks good, good review! | 200g Chaoshan Style Beef Balls*1bag |
| E8 | Very satisfied with a shopping, good business service, fast delivery, logistics is also very powerful, baby received very much like, buy assured, with peace of mind! | 200g Chaoshan Style Beef Balls*1bag |
| E9 | Yummy tired, express me quickly, taste good, if there is an event will repurchase, the | 200g Chaoshan Style Beef Balls*1bag |
| E10 | The quality of this is really unexpected, the physical beauty, touch is very good, very recommended Oh~ | 200g Chaoshan Style Beef Balls*1bag |
| E11 | Really super like it, very good quality, as described by the seller! | 200g Chaoshan Style Beef Balls*1bag |
| E12 | The big brand is trustworthy, and it is said that a penny is worth a penny. Often buy, delicious and tasty flavor, hot pot essential shabu shabu, the portion is very full, eat healthy, eat worry, happy, eat, drink and play well! Give good reviews! | 200g Chaoshan Style Beef Balls*1bag |
| E13 | Second time I've purchased, the brand is still more reliable!!!! | 200g Chaoshan Style Beef Balls*1bag |
| E14 | The flavor is very good, always buy his pills | 200g Chaoshan Style Beef Balls*1bag |
| E15 | Cost-effective is very high, you can rest assured to buy, very exquisite workmanship, worth buying! | 200g Chaoshan Style Beef Balls*1bag |
| E16 | Baby received, very surprised, the quality is very good, very satisfied with a shopping. I will continue to buy back, the store is trustworthy! | 200g Chaoshan Style Beef Balls*1bag |
| E17 | Packaging is very good, the quality is very great, the baby is completely in line with the seller's description, you can rest assured that you can buy? Thank you? | 200g Chaoshan Style Beef Balls*1bag |
| E18 | The packaging was very good and the logistics speed was a pleasant surprise. This tender fish ball one is also tasty but my kids don't like it. Taste is different from person to person. | 200g Chaoshan Style Beef Balls*1bag |
| E19 | It tasted good, the kids loved it and ate it in the evening when it arrived in the morning! It was slightly thawed when it arrived in the morning! Would have been better with an extra ice pack | 200g Chaoshan Style Beef Balls*1bag |
| E20 | Jingdong goods logistics is guaranteed, very good. Has been in the Jingdong above to buy daily necessities, commodities are quite complete, and the quality is quite good, the price is very suitable, much cheaper than the supermarket, meet the activities to buy more cost-effective a penny, a penny, the key is the delivery speed is very fast, direct delivery downstairs, do not have to carry their own, it is very convenient, very convenient. | 200g Chaoshan Style Beef Balls*1bag |
| E21 | You must have croquettes to eat hot pot, and to eat croquettes must be Yasui's, which is really delicious ~ you can eat hot pot, spicy hot pot, and spicy hot pot. | 200g Chaoshan Style Beef Balls*1bag |
| E22 | A particular favorite locking pill? , not comparable to other bulk packs. Used to buy a pack at the supermarket is the original price, not much discount. Jingdong activities when stock up some, really good value. | 200g Chaoshan Style Beef Balls*1bag |
| E23 | It's a great buy for the event, and it's great for fondue lovers! | 200g Chaoshan Style Beef Balls*1bag |
| E24 | Quick dish, make a spicy hot pot, make a sample to go down a noodle are very fast and delicious! | 200g Chaoshan Style Beef Balls*1bag |
| E25 | The fish balls were delicious, came over a little melted but okay not all melted. | 200g Chaoshan Style Beef Balls*1bag |
| E26 | Favorite food is this croquettes, baked or boiled, both are delicious! | 200g Chaoshan Style Beef Balls*1bag |
| E27 | Stock up every time there's an event. The beef meatballs are very solid, tasty, and popping. | 200g Chaoshan Style Beef Balls*1bag |
| E28 | I bought it for an event and it was a great deal, and Yasui's stuff is delicious. | 200g Chaoshan Style Beef Balls*1bag |
| E29 | I received the fish roe croquettes, they were delicious, I loved them and will be back next time. | 200g Chaoshan Style Beef Balls*1bag |
| E30 | Very good, will repurchase and recommend to friends | 200g Chaoshan Style Beef Balls*1bag |
| E31 | Quite delicious, this before buying bought, my father bought. Inside the supermarket is quite expensive, quite delicious. | 200g Chaoshan Style Beef Balls*1bag |
| E32 | The croquettes are better, I like them. Tastier than others of the same price! | 200g Chaoshan Style Beef Balls*1bag |
| E33 | Quality assurance is Yasui's greatest strength and I hope it can be maintained. | 200g Chaoshan Style Beef Balls*1bag |
| E34 | It was fast, during the epidemic. The fish tofu at Yasui's was really good. After eating Yasui's, I don't want to eat anything else. | 200g Chaoshan Style Beef Balls*1bag |
| E35 | These croquettes are really good, everyone in the family loves them! | 200g Chaoshan Style Beef Balls*1bag |
| E36 | I've bought it again, my son loves it! | 200g Chaoshan Style Beef Balls*1bag |
| E37 | Yasui is an old favorite and has always been a favorite. The packaging was great, with additional ice packs to ensure freezing. | 200g Chaoshan Style Beef Balls*1bag |
| E38 | It's good. I've always had it. | 200g Chaoshan Style Beef Balls*1bag |
| E39 | Fishballs received, good quality, fast logistics | 200g Chaoshan Style Beef Balls*1bag |
| E40 | Both the stir fry and the fondue were great! | 200g Chaoshan Style Beef Balls*1bag |
| E41 | It's delicious. I buy Yasui's hot pot meatballs now, so I'm sure they're delicious. | 200g Chaoshan Style Beef Balls*1bag |
| E42 | I bought a lot of them at the event, it's convenient and delicious! | 200g Chaoshan Style Beef Balls*1bag |
| E43 | The beef meatballs were delicious and the kids loved them! | 200g Chaoshan Style Beef Balls*1bag |
| E44 | Take advantage of the offer and repurchase again, a must have for spicy hot pots and hot pots. | 200g Chaoshan Style Beef Balls*1bag |
| E45 | Fish balls are very delicate and delicious, will buy again! | 200g Chaoshan Style Beef Balls*1bag |
| E46 | Received, very good, very like, worth buying, (*^__^*) hee hee ...... | 200g Chaoshan Style Beef Balls*1bag |
| E47 | This one is the best, so let's evaluate it separately | 200g Chaoshan Style Beef Balls*1bag |
| E48 | Yasui's meatballs taste good, and I remember there was a review that Yasui met all the standards, so I feel more comfortable eating them. | 200g Chaoshan Style Beef Balls*1bag |
| E49 | The real deal tastes good and the delivery is fast | 200g Chaoshan Style Beef Balls*1bag |
| E50 | Yasui food often buy, delicious! | 200g Chaoshan Style Beef Balls*1bag |
| E51 | I've purchased it many times, my daughter loves it, and Yasui's is still more reliable! | 200g Chaoshan Style Beef Balls*1bag |
| E52 | Very good and tasty, repurchased several times | 200g Chaoshan Style Beef Balls*1bag |
| E53 | I've always eaten this brand, and the balls are delicious, kudos! | 200g Chaoshan Style Beef Balls*1bag |
| E54 | Good flavor, popping and smooth texture | 200g Chaoshan Style Beef Balls*1bag |
| E55 | Chewy, good flavor,,. | 200g Chaoshan Style Beef Balls*1bag |
| E56 | Third time I've bought it and the stuff tastes great | 200g Chaoshan Style Beef Balls*1bag |
| E57 | My coworkers say that Chaoshan Beef Meatballs are delicious and have an old family flavor! | 200g Chaoshan Style Beef Balls*1bag |
| E58 | Q-bouncy, lubricated fish flavorful, just the right amount | 200g Chaoshan Style Beef Balls*1bag |
| E59 | This is the most expensive and best tasting meatball in the Yasui lineup. It is chewy and tender, and has a lot of meat content and flavor! | 200g Chaoshan Style Beef Balls*1bag |
| E60 | Solid beef with a really nice, chewy texture! | 200g Chaoshan Style Beef Balls*1bag |
| E61 | Very good pills, branded with quality assurance, very good, pretty good | 200g Chaoshan Style Beef Balls*1bag |
| E62 | Hesitated for a long time before deciding to order and I wasn't disappointed! | 200g Chaoshan Style Beef Balls*1bag |
| E63 | Tasty, inexpensive, convenient and affordable products | 200g Chaoshan Style Beef Balls*1bag |
| E64 | Yasui products, must be genuine, worth buying! | 200g Chaoshan Style Beef Balls*1bag |
| E65 | Always buy this brand of croquettes, the family loves them! | 200g Chaoshan Style Beef Balls*1bag |
| E66 | The flavor is pretty good. The quality is good. | 200g Chaoshan Style Beef Balls*1bag |
| E67 | The more authentic flavor of Yasui's Locked Fresh Dumplings | 200g Chaoshan Style Beef Balls*1bag |
| E68 | The stuff is delicious, will buy again next time | 200g Chaoshan Style Beef Balls*1bag |
| E69 | Very tender fishballs, only Yasui for fishballs! | 200g Chaoshan Style Beef Balls*1bag |
| E70 | It's good. I've bought it several times. It's good. | 200g Chaoshan Style Beef Balls*1bag |
| E71 | It's good, kids love it! It's good. | 200g Chaoshan Style Beef Balls*1bag |
| E72 | Delicious, recommend it, will keep buying back | 200g Chaoshan Style Beef Balls*1bag |
| E73 | Sturdy packaging, tasty. Not bad. | 200g Chaoshan Style Beef Balls*1bag |
| E74 | Always buy Yasui's stuff, it tastes good? | 200g Chaoshan Style Beef Balls*1bag |
| E75 | The flavor is very good oh, is genuine, is a little bit expensive, later can more activities, discount a bit of chanting | 200g Chaoshan Style Beef Balls*1bag |
| E76 | Yummy, my husband is a very picky eater and when he says this beef meatballs are good, they are really good. | 200g Chaoshan Style Beef Balls*1bag |
| E77 | Shipped quickly, packaged well, tastes great, great companion for cooking. | 200g Chaoshan Style Beef Balls*1bag |
| E78 | Kid's favorite Stock up on a few more packs when there's an event Tastes good. | 200g Chaoshan Style Beef Balls*1bag |
| E79 | I ate it all, didn't bother to comment, it was quite tasty. | 200g Chaoshan Style Beef Balls*1bag |
| E80 | Fast shipping, cook noodles and dumplings put a few to enrich the taste, very tasty! | 200g Chaoshan Style Beef Balls*1bag |
| E81 | It's delicious, I'll buy it again. | 200g Chaoshan Style Beef Balls*1bag |
| E82 | Bought it at the event price, which is fine for the price. | 200g Chaoshan Style Beef Balls*1bag |
| E83 | Great quality, multiple purchases, would buy again | 200g Chaoshan Style Beef Balls*1bag |
| E84 | The flavor is very good, always buy it,,. | 200g Chaoshan Style Beef Balls*1bag |
| E85 | Q-bomb, Yasui brand is still good, more cost-effective than supermarkets | 200g Chaoshan Style Beef Balls*1bag |
| E86 | This is a croquette that kids love to eat. Recommended to buy. | 200g Chaoshan Style Beef Balls*1bag |
| E87 | Yasui Foods, trustworthy and delicious | 200g Chaoshan Style Beef Balls*1bag |
| E88 | Yasui is a reputable brand, good flavor, will repurchase | 200g Chaoshan Style Beef Balls*1bag |
| E89 | Delicious, flexible, will repurchase | 200g Chaoshan Style Beef Balls*1bag |
| E90 | Fast logistics, good freshness of goods, good taste | 200g Chaoshan Style Beef Balls*1bag |
| E91 | Delicious, good portion, affordable, always buy, recommend! | 200g Chaoshan Style Beef Balls*1bag |
| E92 | The croquettes are delicious and worth the price. | 200g Chaoshan Style Beef Balls*1bag |
| E93 | Repurchase often, buy when there is an event, love it! | 200g Chaoshan Style Beef Balls*1bag |
| E94 | Everyone in the family says it's delicious. | 200g Chaoshan Style Beef Balls*1bag |
| E95 | Yummy. Not bad. Fondue, for soup, it's all good. | 200g Chaoshan Style Beef Balls*1bag |
| E96 | Goods received very satisfied, the business is very careful, with foam box and ice pack packaging, goods received very fresh. My first purchase, eat well and come back to buy. | 200g Chaoshan Style Beef Balls*1bag |
| E97 | It's not bad. I've always had Yasui's croquettes. | 200g Chaoshan Style Beef Balls*1bag |
| E98 | It's still Anjin's good, pure flavor | 200g Chaoshan Style Beef Balls*1bag |
| E99 | My son has always loved this brand | 200g Chaoshan Style Beef Balls*1bag |
| E100 | Loved the Yasui and will be back for more! | 200g Chaoshan Style Beef Balls*1bag |
| E101 | I've bought the beef meatballs many times, love them! | 200g Chaoshan Style Beef Balls*1bag |
| E102 | I tried other brands and the flavor and ingredients are really different from this one, so I'd rather eat this brand without worrying about it | 200g Chaoshan Style Beef Balls*1bag |
| E103 | Yasui's beef meatballs are really good. | 200g Chaoshan Style Beef Balls*1bag |
| E104 | Sturdy packaging and delicious. | 200g Chaoshan Style Beef Balls*1bag |
| E105 | Great brand that I've been buying for a long time | 200g Chaoshan Style Beef Balls*1bag |
| E106 | It's good. I'll buy it again. | 200g Chaoshan Style Beef Balls*1bag |
| E107 | It's delicious. It's the real deal. | 200g Chaoshan Style Beef Balls*1bag |
| E108 | Great taste, very tasty will buy more in the future | 200g Chaoshan Style Beef Balls*1bag |
| E109 | The texture of the meatballs is exceptionally good, oily but not greasy, the flavor is very fresh and tasty must buy it, have repurchased it many times! | 200g Chaoshan Style Beef Balls*1bag |
| E110 | The texture of the croquettes is something I haven't experienced on other places, and it was really, really good. | 200g Chaoshan Style Beef Balls*1bag |

| serial number | Word-of-mouth text (Pickled Fish F) | offerings |
| --- | --- | --- |
| F1 | Very good, bought many times? Affordable and convenient, the fish and chips taste very good, silky and delicate, lips and teeth, Jingdong self-supporting delivery is very fast, the next day, the courier brother delivery, eat and then repurchase! | Sauerkraut 419g |
| F2 | The flavor of this pickled fish is very good and it's also a quick dish especially suitable for me as a lazy person. The meat of the fish fillet is also very tender and the flavor is very authentic. Will continue to repurchase praise, praise. | Sauerkraut 419g |
| F3 | Very convenient and quick dish, second purchase already, you can add your own bean skins and enoki mushrooms, kitchen hacks can make it, this is a good choice when you don't have time to cook. | Sauerkraut 419g |
| F4 | I've bought this many times, and this is the most unique pickled fish in Yasui's fast food. The flavor is really good, and the sauce is very good, so it's not much different than what you'd find outside, so it's good for home cooking! | Sauerkraut 419g |
| F5 | This brand is needless to say, it is for this brand to come, at least rest assured. It's much more affordable than the supermarket! The taste is also very awesome, like me these kitchen little white can also easily manage. | Sauerkraut 419g |
| F6 | Fish fillets have been received, quick dish to do up multiply is very convenient, for me a person without culinary skills can also be convenient to get started, fish fillets delicate and soft, taste good, after eating the aftertaste, Jingdong self-supporting delivery is very fast, the next day to, courier brother delivery. | Sauerkraut 419g |
| F7 | Wow, only you can not think, no you can not buy, this pickled fish is too convenient, two people points enough flavor, delicious, fast cooked dishes, do not have to test the technology, white people can easily get started, seconds to become a chef at this moment, the meat is delicious and tender. | Sauerkraut 419g |
| F8 | Occasionally a can also open the meat, this convenient and simple, looking quite good. Jingdong Mall discount offers and five bags, enough to eat a while, really quite good, mainly to see the cost-effective. It is very easy to do, ten minutes on the table to eat. The quality of goods is very good, the express service is also a great. Customer service enthusiasm are to. Arrived, pickled fish received, tried to eat. | Sauerkraut 419g |
| F9 | Received, very good, very like, worth buying, (*^__^*) hee hee ...... very satisfied? , material workmanship is very good, and versatile, feel great, very nice, very practical, praise? Shipping speed is very fast, things are well packed, the price is favorable, I am very satisfied? , will buy back | Sauerkraut 419g |
| F10 | Cheap and affordable, buy a lot, baby as always? , cost-effective and very high, big brand trustworthy?  Repurchase many times, pickled fish or this good. But after all, strange, 618 price is not low | Sauerkraut 419g |
| F11 | The second time in the Jingdong purchase, the taste is not bad fish enough fresh pickle flavor authentic enough, the whole family like. During the Spring Festival in the mall bought once a little expensive, inadvertently saw the Jingdong supermarket also has the exact same also cheaper a few dollars! | Sauerkraut 419g |
| F12 | The fish fillets are freshly sliced and already marinated with salt and other condiments, so you can just shabu-shabu them directly, which is convenient and delicious. | Sauerkraut 419g |
| F13 | We all know that Anjing is a big and trustworthy brand in China. No matter adults and children, it is safe to eat it! I've eaten Yasui's meatballs at Hot & Spicy, and this time, I bought the pickled fish at a Jingdong event! No problem. | Sauerkraut 419g |
| F14 | Mr. Anjing Frozen Pickled Fish is a regular and quick dish at home, the fish is fresh and odorless, and the accompanying seasoning packet is easy to use. Jingdong Express is fast, ordered in the morning and received in the afternoon. Will continue to repurchase pickled fish. | Sauerkraut 419g |
| F15 | It can be, quite delicious, lazy man talisman, will not put the seasoning can also be, chatting about the package are ready, convenient and convenient, put together a single to buy, unexpected surprise, not worse than the outside flavor, delicious? | Sauerkraut 419g |
| F16 | Last time, my sister-in-law brought a package of pickled fish, and after trying it, I felt good, and it was not too far from eating in the restaurant? So, this time, I also bought my own to try it, and it can be used as a regular dish in the future. | Sauerkraut 419g |
| F17 | I've purchased the fish and chips many times, it's convenient, no bones and no thorns, so I'm not worried about eating it, I bought the pickled fish packet myself and cooked it for a few minutes, then you can eat it. | Sauerkraut 419g |
| F18 | The quality of Anjing products is still very good, purchased several packages of fish and chips, the taste is okay, is that the taste of pickled fish is very spicy, the seasoning packet is a bit heavy, you can put a little less good! | Sauerkraut 419g |
| F19 | The stuff was great. The fish was easy to cook and put a lot of side dishes on it. Very delicious. Jingdong is often active, cheap, fast delivery, good value. The deliveryman is also quite good. | Sauerkraut 419g |
| F20 | Frozen: Yasui's no-slurry fillets are very convenient. Especially the Jingdong logistics, cold chain packaging, ordered before 12:00 today and received it after 17:00 in the afternoon. Kudos! | Sauerkraut 419g |
| F21 | This pickled cabbage fish flavor is correct, fresh, Anjing do fast food is very bad, the family like to eat, usually buy in the supermarket, Jingdong activities, much cheaper than the supermarket ah, cost-effective! | Sauerkraut 419g |
| F22 | Anjing fish fillets often buy, no thorns elderly children can eat, the production date of the recent, 250 grams of a meal of soup just good, 618 activities are very powerful, Jingdong shopping happy service are very good, like shopping in Jingdong | Sauerkraut 419g |
| F23 | "This sauerkraut prep is such a hit! It was unbelievable that it was snapped up as soon as it was released. I also scrambled to place an order and was lucky enough to get a copy. The taste is excellent, the seasoning is authentic, and it's like tasting authentic Sichuan and Hunan flavors in your home kitchen. | Sauerkraut 419g |
| F24 | Flavorful and fresh, this Mr. Yasui Frozen Pickled Fish is a quick weekly family favorite for the elderly and children. Sauerkraut has an authentic flavor and is easy to cook. Jingdong logistics speed is very powerful, order in the morning and received in the evening. | Sauerkraut 419g |
| F25 | This fish fillet fried to eat very fragrant, very tender, very fragrant, has been adjusted sauce, open without having to get their own, put the pan fried on it, bought a lot to put in the refrigerator to freeze, Jingdong express delivery is also very fast, satisfied, the | Sauerkraut 419g |
| F26 | First, thaw the pickled fish preps naturally, then add 500 ml of water to the pot and bring it to a boil, add Ingredient Pack 1 and Ingredient Pack 2 in that order, mix well and bring to a boil again; then add the pickled fish slices, stir and cook for 90 seconds before removing from the pot; put into a bowl, sprinkle with dried chili peppers and sesame seeds, and use boiling oil to enhance the aroma. A bowl of delicious pickled fish is ready!  Comments: This Mr. Yasui Frozen Pickled Fish Prep can be broiled to rival a restaurant, with four and a half stars out of five for deliciousness; and two and a half stars for ease of broiling. It's still not very convenient for outdoor camping when you burn it yourself, as you need to use an open fire or an induction cooker, and there are too many steps. | Sauerkraut 419g |
| F27 | Sauerkraut fish is super tasty, the base material is great, and outside the sale of sauerkraut fish a flavor, fresh aroma and taste delicious Road good, the family are very like to eat, a few minutes on all eaten, spicy fish will also repurchase, buy less ha ha ha, delivery is also fast, to the home of the ice packs have not been melted, sauerkraut fish is very addictive, love love love! | Sauerkraut 419g |
| F28 | Watching the ads know this brand, buy this pickled fish eaten once feel very good, home to buy a little stockpile, the weekend when their own home can be done, the operation is also convenient, the taste is very good. | Sauerkraut 419g |
| F29 | Customer service enthusiasm are to. The first time to buy Anjing's pickled fish, before eating a good, his family's goods throughout the fresh delivery, basically the next day to reach, is the express fee is too expensive. In short: satisfied! Baby received, the seller shipped very quickly, logistics is also very strong, customer service attitude is excellent, very patient, give a sense of intimacy, very like. I really like it very much, very supportive of it, the quality is very good, exactly as described by the seller, I am very satisfied. Sauerkraut received and tried it. | Sauerkraut 419g |
| F30 | After comparing several different brands, Mr. Freeze's tastes the most authentic. Those who are still on the lookout can get their hands on it without hesitation! The sauerkraut has a great flavor, the fish is fine with very few spines and a crunchy sweet taste. You can see that the seller is very careful. Quite good, satisfied. This is a good review generic version. | Sauerkraut 419g |
| F31 | Sauerkraut fish production is simple, clear steps, sit up easy super tasty, fish is a little less, two packages to put together a package, the rest of the separate and then buy black fish to eat, soup is very fresh, never get tired of eating. Jingdong Express, home delivery, convenient and fast! | Sauerkraut 419g |
| F32 | Sauerkraut is delicious! Summer weather is hot, and sour and spicy is just right, can hang the taste, otherwise can not eat. The main thing is to buy things fast on top of the Jingdong, and work hard for the delivery boy! | Sauerkraut 419g |
| F33 | Received the goods cleaned can be directly cooked, very convenient, pickled cabbage packets are very full of flavor, spicy and refreshing family are very like, the follow-up will continue to repurchase, Jingdong logistics is super strong! | Sauerkraut 419g |
| F34 | Can't remember how many times I've repurchased it, every weekend I make it for my son to eat, the young man who is growing up, every time he eats it very happily, add your favorite dishes in it, it's easy and quick to do, and the taste is not worse than the Szechuan restaurant, it's awesome! | Sauerkraut 419g |
| F35 | It's good, it's great, it tastes great,, it's easy to make, it's delicious, it's also exceptionally fresh, it's cheap and affordable, it's good quality, it arrives quickly, the courier worked hard,, thanks for that. | Sauerkraut 419g |
| F36 | It's already countless times of repurchase, bought for my mom, very tasty and fresh, high quality and low price, value for money, very fresh and easy to make, very good business, wishing business prosperity. | Sauerkraut 419g |
| F37 | The mouth rice was great, a little spicy, pretty full of flavor, bought another single serving of fish and chips to add to the mix and it still tasted fine. I'll be back for more, next time I'll try to put in two more portions of fish and chips to try. | Sauerkraut 419g |
| F38 | Delicious, one bite, can not stop, good taste, has been drinking, the effect is good, next time to buy back, five-star praise, next time to continue to patronize, the price is cheap, a penny a penny it. | Sauerkraut 419g |
| F39 | This Mr. Frozen Pickled Fish is very good, it has been purchased many times, the pickled fish base is fresh and spicy, the fish fillets are tender, and then put some of your favorite side dishes, it is very easy and quick to make, very satisfied! | Sauerkraut 419g |
| F40 | Good pickled fish, bought several times, each time did not let me disappointed, very satisfied. I will continue to buy back later, I hope more activities. Will often stock up, and finally praise the Jingdong logistics. | Sauerkraut 419g |
| F41 | The quality of a good mess, some were surprised, even more than many outside restaurants do pickled fish is very good, very surprised, I did not expect with the cure dish has been done to this extent in the future will often buy back! | Sauerkraut 419g |
| F42 | Buy, on the end, this flavor absolute absolute son, to the home of the mother to buy a copy of the day sister-in-law is also in, to the side and put the balsa fish, broad noodles, shrimp slip, dripping oil when put some cilantro, taste thief great, sister-in-law huge like, come back to buy their own, put the fungus, enoki mushrooms, cauliflower, put a catty of balsa fish, seasoning only a separate added salt, the taste is very good, ate the super favorite, buy, buy, buy, buy! ...stock up! | Sauerkraut 419g |
| F43 | Definitely the happiest shopping, the store has a very good service attitude, after-sales is also a very trustworthy brand, the quality is guaranteed, the price is also pro-people, quite satisfied with has been many times back to buy, recommended! | Sauerkraut 419g |
| F44 | It's a very convenient dish that allows even those who can't cook to become chefs, it tastes just as authentic as outside, it's delicious, it's easy to make, and this is much more affordable compared to what's out there. Maybe the ones outside are similarly half-baked | Sauerkraut 419g |
| F45 | Fish fillets received, business services carefully and seriously, the activities of the concessions, Jingdong logistics delivery speed, Jingdong express brother is very strong throughout the cold chain delivery to the home, here a praise, always eat this brand of products, fresh flavor taste good, need to continue to buy back. | Sauerkraut 419g |
| F46 | I've purchased this product many times, and the main thing is that it's easy and convenient. The fish are cut and mixed, directly according to the order of the package of ingredients into it, and then add a little bit of their favorite side dishes, a plate of delicious food is ready. Really super convenient. Full cold chain logistics, taste really good? | Sauerkraut 419g |
| F47 | Mr. Yasui's Pickled Fish is called a "quick dish". In fact, it is simple, easy to operate, and requires no cooking skills. Moreover, it saves time and labor. The fish fillet is strong and smooth, the quality of sauerkraut is reliable, and the soup has a good flavor. Give praise. | Sauerkraut 419g |
| F48 | The family has been repurchasing the fish fillets, do sour soup fish fillets really too suitable, no pulp, convenient and hygienic, Anjing's big brand, Jingdong express delivery is also good, the little brother sent over when it is still frozen, the | Sauerkraut 419g |
| F49 | Prepared dishes are very convenient and fast, Jingdong logistics is very fast, I like to eat pickled fish, and lazy to spend too much time to do, this kind of semi-finished products is very suitable for me, soon to eat, lazy people must have! Recommended! | Sauerkraut 419g |
| F50 | Usually super love pickled fish, but takeout is too expensive. Friends recommended this first time to buy, shipping to the goods are quite fast, the Jingdong own courier brother also delivered to the door, five-star praise! Look at the tutorial on the package to do it is still quite convenient! Good if you buy again! | Sauerkraut 419g |
| F51 | The quality of the big brand is assured, the flavor is delicious, and it has been purchased many times. The whole family loves it. The big platform of Jingdong is trustworthy, and the quality and service are satisfactory. The logistics guy is super praise, enthusiastic service. The first day of the order, the next day to receive. Super praise. | Sauerkraut 419g |
| F52 | I've had it once before and it tastes good, almost as good as in a restaurant. And it's very easy to heat up! The broth is enough that you can add your own sliced tofu, fungus, enoki mushrooms, bok choy, etc... A hard dish, done in minutes? | Sauerkraut 419g |
| F53 | The fast food was convenient and the pickled fish was a classic flavor. Comparing several different brands, Mr. Frozen's tastes the most authentic. My daughter loves it, and it's a must-have for stocking up on discount promotions these days. | Sauerkraut 419g |
| F54 | Genuine, good quality, low price, high sex-ratio, good value, value for money, friends look good. Shipping is fast, has received the goods, goods and description of the same, the quality is no problem, the key price is also appropriate, buy here or very assured, praise! Occasionally a can also open a meeting, this convenient and simple, looking quite good. Customer service enthusiasm are to. Packaging is very good, courier force, the seller is very reputable, delivery speed is also very fast. See people say Anjing pickled fish is delicious, really, fragrant me, which also has a hot pot store to buy fish fillets, not as good as the self-serve fish fillet package, I also added bean sprouts and tofu skin and bok choy, too delicious, but the taste is a little salty Oh, to put more water. | Sauerkraut 419g |
| F55 | This pickled fish is still quite good, the packaging is very tight, the instructions are also quite adequate, the fish is also very fresh, hot and sour degree can be, not too spicy quite tasty, the portion is very full, a family of three people enough! | Sauerkraut 419g |
| F56 | This pickled fish is very beautifully made,,, the flavor is very good, the texture is delicate and unbeatable, the price is also very fair, something very good, colleagues with free fish fillets, recommended to buy, hahahahahahahahahaha! | Sauerkraut 419g |
| F57 | Mr. Yasui's Pickled Fish is called a "quick dish". In fact, it is simple, easy to operate, and requires no cooking skills. Moreover, it saves time and labor. The fish fillets are strong and smooth, the sauerkraut is of reliable quality, and the soup has a good flavor. Give a favorable comment. | Sauerkraut 419g |
| F58 | Arrived, received the sauerkraut and tried it. Sauerkraut flavor is very full, the fish is fine, very few spines, crisp and sweet. It is very easy to do, ten minutes on the table to start eating. Overall a bit on the spicy side, the amount of fish is a bit small, overall it is still good! | Sauerkraut 419g |
| F59 | Sauerkraut fish in the confirmation of sauerkraut, you can also add some other dishes to eat together, put some beanskin what, Anjing brand taste better, a variety of dishes are not bad, eat at ease, at home in the kitchen whites can also get up on their own | Sauerkraut 419g |
| F60 | The unlimited repurchase pickled fish series is also one of the unlimited repurchase Yasui series. After the epidemic it has become harder to get under 20, so stock up appropriately when you come across a cheap price for convenience. | Sauerkraut 419g |
| F61 | I received the sauerkraut and tried it. Sauerkraut flavor is very full, the fish is fine, very few spines, crisp and sweet. It is very easy to do, ten minutes on the table to eat. Overall a bit on the spicy side, the amount of fish is a bit small, overall still good! | Sauerkraut 419g |
| F62 | I do not remember how many times I bought, I do not remember how many little friends I recommended, anyway, continue to love to eat, this material, for me not so much can eat spicy people, it is true that a little spicy, but the flavor of the bar, eat and hurry to hoard, the price is affordable, from now on to fall in love with pickled mustard fish, is that the fish fillets have thorns, is not perfect, if there is no thorns, it's even more perfect! | Sauerkraut 419g |
| F63 | Sauerkraut fish purchased in honor of the brand name of Anjing, read the reviews that the amount of fish fillets, sauerkraut is quite good have not yet opened to eat, as for the taste of how, personally cooked and then come back to the review! | Sauerkraut 419g |
| F64 | This is the second time I've purchased it, and the flavor is quite good, comparable to restaurants, and the quantity is quite good, but it's a bit spicy.  The BOE delivery in this area has been good, delivered on time, to the floor, with cold chain delivery. | Sauerkraut 419g |
| F65 | I read a lot of pickled fish on the internet, and there are a lot of bad reviews inside the review section, only this brand has good reviews, so I placed an order to try it out. I hope everyone's reviews are real and credible. Good food will continue to buy. | Sauerkraut 419g |
| F66 | Unlimited repurchase of pre-made dishes! Cooking this sauerkraut fish is not much better than takeout ah ...... It's delicious and convenient, and our whole family loves it. With this I never order pickled fish takeout again, hahaha ...... You can also add some bean sprouts and other side dishes, completely restaurant output. | Sauerkraut 419g |
| F67 | Express delivery is still frozen, the flavor is okay, the amount of fish in a bag is a little less, and added a bag of fish to cook together, but also add their own side dishes, even if you do not know how to cook can also be completed, cooking is very convenient | Sauerkraut 419g |
| F68 | The ingredients are complete, chili peppers, pepper and sesame seeds, only need to prepare a little green onion can be, the fish is very sufficient, the only shortcoming is that the fish slices are not too meticulous, there will be a lot of broken thorns, but the overall taste is very good, the practice is very simple | Sauerkraut 419g |
| F69 | Repurchase too many times, this year the New Year and bought 3 copies , simple and fast Easy to operate the basic 5.6 minutes on the good.  This for no need to thaw directly out to see a good step 1.2.3  1.2 together into the pot, the water does not have to be particularly much, and so open the pot for 1.2 minutes to add 3, yes that's right is frozen fish fillets, the whole into it, do not have to worry about direct cover.  About 1.2 minutes to open the lid, you can see the fish is cooked, take a spoon to pick and gently stir, the fish will be separated from the fillets will not be broken at all.  I like to eat big ramen I put some, like to eat other can also be put.  The operation is simple and suitable for New Year's Eve and usually do not have time to prepare meals for the family, or guests come to the preparation of a short time can also be immediately operated. | Sauerkraut 419g |
| F70 | Super yummy. This brand has a good portion size and authentic flavor. Adults and children eat all, healthy and nutritious. Hey, hey, hey, buy two bags at once. Inside the chili package, you can add according to their own tastes, my family is not too much love chili, this flavor can be less to put the package. | Sauerkraut 419g |
| F71 | It's easy and convenient to make, the steps are printed on the back of the pouch, just follow the steps to make 10 minutes and you can have pickled fish with the same flavor as outside. The small bag inside is also very carefully printed on the first few steps. You can also first put the oil to the chili peppers, pickles fried, and then add water to boil under the fish fillets. | Sauerkraut 419g |
| F72 | These two are always at home, tried many kinds, these two have the best flavor, those who like it can try, absolutely no rollover. These two are always at home, tried a lot of kinds, these two have the best flavor, like people can try, absolutely no cartwheel. | Sauerkraut 419g |
| F73 | Sauerkraut fish portion is very sufficient Oh, is the portion of 2 people, you can also add your favorite side dishes, and fragrant and spicy, it is simple fast food, delivery speed is also very fast, delivered over or ice. | Sauerkraut 419g |
| F74 | This sauerkraut is absolutely perfect. Very convenient. Just follow his steps 1-2-3-4. The gospel of lazy people. Just how to cook will not turn over, comparable to many offline network red store. Favorable comments ah | Sauerkraut 419g |
| F75 | This Mr. Frozen Pickled Fish is really so good? Through many comparisons, I feel that his flavor is still good! Tender and deliciously smooth! The main ingredient fish fillet is very fresh, and the side dishes of sauerkraut and small ingredients are also very delicious? , more than enough for two people to eat! Production is also very simple, do smooth ten minutes to get done, can be accompanied by soybean sprouts lettuce? The flavor is very satisfactory! Recommended to buy! | Sauerkraut 419g |
| F76 | Anjing pickled fish is well packaged, delivered quickly and comes with all the ingredients. Easy to make and easy to eat. The flavor is good, sour, spicy and fragrant, the fish fillets are fresh, smooth and tasty, the whole family loves it. If you can't eat spicy, put less seasoning. Five stars! | Sauerkraut 419g |
| F77 | It's pretty good, with bacon together more flavorful. Feeling quite good, quite convenient, you can point point eyes, the portion is not too big, the price and when a little discount to come, you are good, quite good. Occasionally come one can also open a meeting, this convenient and simple, looking quite good. Still can not say good to eat. A year is big enough and appetizing. | Pickled Fish 419g/box |
| F78 | This is a pre-made dish, the main ingredients and toppings are made up, buy it and make it slightly, basically no need to add seasoning. There are four packets in a box: sauerkraut seasoning sauce (1), old-fashioned sauerkraut (2), fish fillets (3), chili peppers and sesame seeds. Each small packet has a reminder of the order of preparation on the bag, and the ones that don't have an order reminder are the ones that go down last, which is very thoughtful. Sauce packet, sauerkraut and pepper chili according to personal taste, you can pour all of them, you can also put in a moderate amount. The fish fillets are added to the pot and cooked, only about a minute and a half is enough, do not need to cook for too long. The finished product comes out of the pot and tastes fantastic and can be served with rice or noodles. Recommended~ | Sauerkraut 419g |
| F79 | During the New Year's Eve mom let buy pickled fish, taste very good, sour and spicy flavor, I like, there is a packet left, ready to buy a few more packets, Anjing's assured to eat, which goes back to the purchase. | Sauerkraut 419g |
| F80 | It's easy to do.  And you can add some of your favorite dishes to it, it's quite delicious, the flavor is okay, but I feel that the sauerkraut is not sour enough!  And the ingredients and so on is also very complete, do it quickly, very convenient ~ | Sauerkraut 419g |
| F81 | Since I bought Anjing pickled fish in Jingdong, the family never ordered pickled fish takeout, it is really convenient and affordable, good taste, easy to operate, adults and children love to eat. | Sauerkraut 419g |
| F82 | Anjing's products, no matter what, are more assured, this quick dish, is pickled fish, very convenient, heating that is eaten, very simple and convenient, will not cook the white man can also make the chef's delicious! | Sauerkraut 419g |
| F83 | Mr. Anjing Frozen Pickled Fish, Jingdong this time the price is very strong, cheaper than RT-Mart 1/3, the production is also convenient, do not want to cook, take out a bag of defrosted, a few minutes to the table to enjoy. | Sauerkraut 419g |
| F84 | Yummy tastes really good. Not expensive and delicious. The big platform of Jingdong is trustworthy, shopping is assured, the quality is guaranteed, and the service is good. The logistics guy is super awesome, and the service is enthusiastic. Give you a recommendation! Buy no regrets. | Sauerkraut 419g |
| F85 | Anjing, Mr. Frozen Pickled Fish, the taste is good, a meal to eat a packet is just right, the fish fillets are particularly tender and fresh, there is not much fish spines, the children can also eat, the Jingdong logistics is very fast, and is very satisfied with an online purchase! | Sauerkraut 419g |
| F86 | Mr. Frozen Pickled Fish, direct heating can be eaten, convenient and fast, the taste is okay, bought a few times, the overall good, New Year's home to prepare some, it is very good, good value for money and convenient and fast quite good. | Sauerkraut 419g |
| F87 | Convenient convenient very convenient! Yummy yummy yummy very yummy! I relied on it during the epidemic, I couldn't even pour out the soup, it all dried up! You can see how it tastes, right? You have to buy it if there's an event, and you have to buy it if there's no event! | Sauerkraut 419g |
| F88 | It's the second time to buy Mr. Frozen, the taste is really good, and it's really convenient, after defrosting, fry the ingredients with oil to make them more flavorful, and then drizzle some hot oil on it at the end, it's perfect. | Sauerkraut 419g |
| F89 | This pickled fish is really awesome as a quick dish. It doesn't take too much effort to operate, it's a delicious flavor that you can enjoy in a few minutes, and the fish is tender and the portion size is enough that I'll serve it with vegetables inside every time. | Sauerkraut 419g |
| F90 | The packaging is consistent with the picture display. The courier boy delivered promptly and quickly, the flavor was great, the package was exquisite and the food looked fresh. This is my favorite, I've had it many times, it's tasty and clean and hygienic. Honestly, this is the most satisfied order since I ordered from the platform. I was very happy with the speed of delivery by the courier, the beautiful packaging and the delicious food. There really aren't many affordable stores like this one. | Sauerkraut 419g |
| F91 | Cost-effective, I like it, next time will come back with the entity store no difference, but also cheap, like! Baby received, logistics is very fast, the description matches, more satisfied, praise, recommended goods in line with the description, the main activity is cheap, can save a lot of money! | Sauerkraut 419g |
| F92 | It has been many times to buy this Mr. Frozen fast food, pickled fish is very authentic, as a Sichuanese feel very delicious and full of flavor, and with a packet of fish fillets, very satisfying, will continue to buy at the event! | Sauerkraut 419g |
| F93 | Mr. Frozen's pickled fish has been repurchased many times, the taste is good, very authentic, the fish fillets are very fresh, no fishy smell, the seasoning is very adequate, the degree of spiciness can be adjusted by yourself, convenient! | Sauerkraut 419g |
| F94 | Tasty and inexpensive, bought on New Year's Day, feel very cost-effective, taste really good, cook a pickled fish hot pot, taste no comment, fast shipping, well-packed, timely delivery, very satisfied, full five praise! Will buy back. | Sauerkraut 419g |
| F95 | Delivery speed is fast, and the date is fresh. Jingdong logistics delivery speed is very fast, the courier boy has a good service attitude. Believe in the Jingdong self-supporting goods, has been buying, buy nothing wrong, need to evaluate too many things, not one by one evaluation, habitually all give favorable comments. | Sauerkraut 419g |
| F96 | One bite into the soul! This pot of sauerkraut is even soupy!  I love sauerkraut fish so much, and recently I discovered a sauerkraut fish that any kitchen novice can make, Anjing Sauerkraut Fish. From opening the bag to serving, it's ready in a few minutes, the fish fillets are white, tender and smooth, spicy and fresh, sour and appetizing! | Sauerkraut 419g |
| F97 | I have bought several kinds of pickled fish, compared to this Mr. Frozen Pickled Fish, the taste is really good, quick dish, especially convenient, easy to operate, the taste is also good, the fish fillets are quite fresh ~ | Sauerkraut 419g |
| F98 | Mr. Yasui Frozen Pickled Fish is very delicious, the fish fillet is very tender, and it is easy and convenient to cook, there is an illustration on the box with instructions in four step-by-step steps, and everyone in the family loves to eat it, it is very good! | Sauerkraut 419g |
| F99 | Tastes good, easy to prepare, mildly spicy, good for dinner, tender fish fillets, very tasty, fast shipping, good quality fish, fresh, good texture, nice base, good value for money, good price, worth the money. | Sauerkraut 419g |
| F100 | Super awesome, thawed directly into the pot, water boiled and put the ingredients, the taste and eat in the restaurant pickled fish, but this cost-effective, a bag is probably enough for a person to eat, the picture is another extra bag of sashimi delicious recommended! | Sauerkraut 419g |
| F101 | The first time to buy pre-made vegetables pickled fish, really too delicious, did not think that actually will be so delicious, do not know what additives more or less, but really too delicious, will be unlimited repurchase hehe? | Sauerkraut 419g |
| F102 | I can't remember how many times I've repurchased it, I haven't eaten any other pickled fish, I just think that Yasui's must be hygienic and tasty, and it's very convenient to process it, and you can add your favorite ingredients as you like, so you don't need to order takeout anymore. | Sauerkraut 419g |
| F103 | Spicy and refreshing, sauerkraut is also very tasty, affordable, logistics is also very fast, affordable, put in the pot to cook two minutes to cook, suitable for not wanting to cook when a quick to create a beautiful meal. | Sauerkraut 419g |
| F104 | Pickled fish quality first-class, packaging is also good, logistics is fast, and the price is reasonable, very affordable, very much like every time is very satisfied, this time to choose the baby is even more satisfied, the future will be the same as in the future to buy. | Sauerkraut 419g |
| F105 | I bought the stuff, looks good, I wanted to do it last weekend, who knows something did not have time to get. Very love to eat boiled fish, see this review is good, buy ready to taste. Express logistics is good, praise, delivery is fast. | Sauerkraut 419g |
| F106 | Jingdong logistics delivered to the door, do not have to go out to eat pickled fish, really cool ah, and the taste is also so good, tear open the package to the pot a cook, save time and money, very delicious! | Sauerkraut 419g |
| F107 | Originally very worried about such a hot day to receive will not be bad, delivery is very timely inside is not all melted, packaging is very heartfelt, this in the fish fillet is more delicious than others, meat thicker! You can simply cope with it at home, but it's also delicious. Enjoy! | Sauerkraut 419g |
| F108 | This sauerkraut is delicious, tart and flavorful, easy and simple to make, and the whole family loves it. It has been repurchased many times and is a must-have pre-made dish at home. Jingdong logistics is very powerful, delivery is very fast. | Sauerkraut 419g |
| F109 | Packaging is very sturdy, things are well protected, worthy of praise shipping very quickly, has received the goods, goods and description of the same, the quality is no problem, the key price is also appropriate, buy here or very assured, praise! Baby received, logistics is very fast, the description is consistent, more satisfied, praise, recommend! | Sauerkraut 419g |
| F110 | Also have to add a little chili and garlic and then splash oil together to have that kind of feeling, eat or very tasty, sauerkraut quality is very high, much better than the kind of material sold at the fish stalls, that is, the fish is very easy to eat out of the frozen. | Sauerkraut 419g |

| serial number | Word-of-mouth text (shrimp G) | offerings |
| --- | --- | --- |
| G1 | Jingdong sold on the emerald prawns, cheap shrimp and big and good, really good. Express delivery to the home, insulation is done very well, the bag of ice did not melt, well packaged. The courier is very enthusiastic and responsible Thank you! | Crystal Jade Raw Shrimp(XL) 1kg |
| G2 | Guolian's emerald shrimp and black tiger shrimp bought two packages each, do different dishes, use different shrimp, once a package of 1kg, just right, basically nothing ice, very cost-effective!!!! | Crystal Jade Raw Shrimp(XL) 1kg |
| G3 | Very good shrimp, the quality of the big brand is reliable, consumption of peace of mind, buy two bags at a time, there are promotional activities, cost-effective, good taste, will buy again in the future, logistics and distribution is also very fast, thoughtful service! | Crystal Jade Raw Shrimp(XL) 1kg |
| G4 | Shrimp is very delicious, large tender, intact, remove the shrimp line, thawed can be cooked, save a lot of trouble, I was fried, fried out of the child loves to eat! Jingdong self-supporting delivery fast service is trustworthy! | Crystal Jade Raw Shrimp(XL) 1kg |
| G5 | This GUO LIAN Jade Raw Shrimp, 1kg net weight without ice, 58-66pcs (BAP certified) domestic, is big enough and has a very good taste, rich in nutrients. | Crystal Jade Raw Shrimp(XL) 1kg |
| G6 | National Union Emerald Raw Shrimp, good quality, very fresh, firm meat, good flavor, have bought several times, cleaned up well, even size, good for stir fry, produced in April 2023! | Crystal Jade Raw Shrimp(XL) 1kg |
| G7 | Emerald prawns are very delicious. The official website of the Jingdong self-owned flagship store is authentic. The old people and children all like it. All kinds of practices can be. Our family holds several times a week. Satisfaction satisfaction satisfaction. Next time to buy again. | Crystal Jade Raw Shrimp(XL) 1kg |
| G8 | Jingdong 618 is having an event, and the Emerald Jumbo Shrimp is $50 off. It was really cheap. Our family loves this jumbo shrimp, so we ordered it while the event was going on. Express delivery to the home, well packaged frozen shrimp does not melt at all. Courier service was great thanks! | Crystal Jade Raw Shrimp(XL) 1kg |
| G9 | Jingdong self-supporting Guolian emerald shrimp, cold chain delivery, full vacuum packaging, freshness is guaranteed. Ice-free net weight shrimp, clean and hygienic worthy of a big brand. Such an easy to make shrimp, thanks to Jingdong and Guolian, providing us with a great platform and service! | Crystal Jade Raw Shrimp(XL) 1kg |
| G10 | Logistics is very fast, timely delivery. Shrimp is very large, tender, no ice and no sand, handled very cleanly, and large, stir-fried vegetables hot pot are a great, the family elderly children are very like to eat. | Crystal Jade Raw Shrimp(XL) 1kg |
| G11 | Guolian aquatic shrimp is very good, very fresh, especially convenient, are single-frozen, not a big pile, eat when you open the bag to pour out some can be, delivered at the time of a little bit did not thawed, the packaging is very good, or Jingdong Express is very good! | Crystal Jade Raw Shrimp(XL) 1kg |
| G12 | Guolian aquatic products of this shrimp is very good, I have even bought a few bags, because it is also very easy to eat, the taste is also very good, with his fried vegetables and so on, and convenient and delicious, the weather is very hot, eat less meat, eat more shrimp, Jingdong delivery is also very convenient, every time to send the shrimp is still very good when frozen | Crystal Jade Raw Shrimp(XL) 1kg |
| G13 | The god of express delivery on the next day to send home, emerald fresh shrimp bags sent also hard awesome, shrimp large and clean are to go to the shrimp line, cost-effective regardless of stir-fry / stir-fry / do shrimp ball soup are as delicious, the main thing is to facilitate the freezing of the hand immediately is a meat Lai, adults and children like taste great! | Crystal Jade Raw Shrimp(XL) 1kg |
| G14 | This shrimp bought a number of times, whether it is to do three dumplings, marinated noodles are put very tasty, shrimp scrambled eggs are the most popular, the Jingdong cold chain transportation is worthy of praise, the first day of the order the next day to, convenient and fast! | Crystal Jade Raw Shrimp(XL) 1kg |
| G15 | It's time to restock again! When you don't know what to eat in the summer, cook some vegetables with shrimp, shrimp don't even need to be thawed in advance, they are taken out directly from the refrigerator, rinsed under water and boiled under the water, and the texture is also very good it. Bouncing teeth texture, moderate size. | Crystal Jade Raw Shrimp(XL) 1kg |
| G16 | It's a great deal for the 618 promotion with the 10 billion subsidized price of just 59.9. Before are 90 buy, then also hoarded a few packages. This shrimp is easy to use, thawed for marinating for a while can be operated, used for stir-fry, do shrimp pasta, etc. can be, the flavor is super awesome, very Q pop. | Crystal Jade Raw Shrimp(XL) 1kg |
| G17 | All National Union products are very good and trustworthy. Waterless Shrimp is certainly no exception. This shrimp is medium sized and can be cooked in a variety of dishes. Shrimp has a high nutritional value and is a high protein, low fat food that is superior to red meat. | Crystal Jade Raw Shrimp(XL) 1kg |
| G18 | The National Union Shrimp is a good size for a variety of stir-fry dishes. This time the price is particularly favorable, can come across especially happy. This shrimp basically belongs to the waterless and ice-free individual shrimp, eat well will often come back later. | Crystal Jade Raw Shrimp(XL) 1kg |
| G19 | This State Union emerald prawns, many times back to buy, mainly because the packaging is good, big head, no ice coating, shrimp is very fresh, used to stir-fry shrimp is very good, but also often with vegetables cucumbers, broccoli shoots, barbecue sprinkled with pretzels, Western-style cream shrimp balls are very tasty, each shrimp are independent of ice coating adhesion to the freezing of the simple, clean and hygienic to eat as much as you want to take as much as it is very easy to eat the Spring Festival to buy a few bags have eaten out! Jingdong 618 activities and buy two bags, if there are promotions in the near future and then buy a few bags In short, this prawns are very good, the price is cost-effective delivery speed. Five-star praise. | Crystal Jade Raw Shrimp(XL) 1kg |
| G20 | The shrimp is exceptionally large, clean and no ice, the texture of the teeth, not the kind of powdered noodles, after purchasing countless times, as always, good, Guolian's emerald shrimp children, although a little expensive, but the quality is really good, catching up with promotions on the stockpile to buy some more, vacuum divided into a few small packages for storage, freshness and save the land. | Crystal Jade Raw Shrimp(XL) 1kg |
| G21 | Shrimp is very good, large and fresh, especially convenient, each time to take out some do shrimp scrambled eggs, shrimp soup, shrimp fried rice, etc., the child loves to eat I also love to eat, is a little expensive, together with the yellowtail and scallops, are very good! | Crystal Jade Raw Shrimp(XL) 1kg |
| G22 | Has been his family's loyal fans, catch 618 when more than a few people buy a few people quite dry, not too much moisture, the main taste QQ very fresh, uniform size, cost-effective than the supermarket cheaper than a lot, continue to pay attention to the points | Crystal Jade Raw Shrimp(XL) 1kg |
| G23 | This is the first time to buy the shrimp, before the purchase of a variety of hesitation and worry, I did not expect to be very satisfied with the receipt of the shrimp, shrimp each very large, very tender, is a good shrimp, the back will be back to buy again! | Crystal Jade Raw Shrimp(XL) 1kg |
| G24 | The Emerald Shrimp from Guolian is a regular in my freezer now. I'll stock up a bit when it's on sale and freeze it to eat slowly. Anyway, it's always delicious.  A bag of 1kg, just to do a dish, shrimp fresh and tender, eat out of the satisfaction, has been recommended to a lot of small friends. | Crystal Jade Raw Shrimp(XL) 1kg |
| G25 | The Black Tiger Shrimp from Guolian is a regular in my freezer now. When there is a sale, I stock up a little bit and freeze it to eat slowly. Anyway, it tastes good any way.  A bag of 1kg, just to do a dish, shrimp fresh and tender, eat out of the satisfaction, has been recommended to a lot of small friends. | Crystal Jade Raw Shrimp(XL) 1kg |
| G26 | The Emerald Shrimp from Guolian is a regular in my freezer now.  I'll stock up a bit when it's on sale and freeze it to eat slowly. Anyway, it's always delicious. A bag of 1kg, just to do a dish, shrimp fresh and tender, eat out of the satisfaction, has been recommended to a lot of small friends. | Crystal Jade Raw Shrimp(XL) 1kg |
| G27 | This Guolian shrimp has been repurchased many times, shrimp size is just right, eliminating the need to peel shrimp every time you eat shrimp, the appearance of the ice coating is only a thin layer, whether it is used for stir-frying, or how to eat it has to be very good, I usually like to stir-fry with tomatoes and eat noodles, especially tasty! Jingdong self-supporting goods delivery is very fast, quality is guaranteed, the price is very affordable! | Crystal Jade Raw Shrimp(XL) 1kg |
| G28 | Baby received it, the physical really good-looking, the price is very affordable, highly recommended this, super like the big brand, with a very reassuring, very good quality, catching up with the activities of the purchase is very cost-effective! | Crystal Jade Raw Shrimp(XL) 1kg |
| G29 | Guolian aquatic jade raw shrimp received, this shrimp packaging is very good, more trust this brand. After the opening of the shrimp is still very large, eat up the texture is also very good, fried to eat super like, a bag inside dozens of it, the head of a large number of. Guolian aquatic emerald raw king-size increase ice-free shrimp positive comments: Baby received very satisfied, taste is also very like, pick a good number of, really did not look at the wrong, repurchase! | Crystal Jade Raw Shrimp(XL) 1kg |
| G30 | Each piece of this frozen shrimp is individually frozen and does not stick to each other. The shrimp are larger in size and the threads are removed by hand. Once thawed, they can be used directly as an ingredient, for cooking. This shrimp is just the right size for stir-frying. Jingdong transportation can keep the whole cold chain to carry out, and the shrimp is still frozen very solid when it arrives home. | Crystal Jade Raw Shrimp(XL) 1kg |
| G31 | Shrimp is good, the flavor is also very tasty, overall value for money, used to scramble eggs fried rice fried vegetables are quite suitable. The child ate quite fragrant, logistics is also very fast, Jingdong is still reassuring, good! | Crystal Jade Raw Shrimp(XL) 1kg |
| G32 | It's a small but ok size, the meat is tender and cooks for 3 or 4 minutes. Convenient and nutritious!  Habitual favorable comments. Baby quality is good, very like it. Thanks to the shopkeeper. Praise a fast shipping, the baby is almost the same as the picture. | Crystal Jade Raw Shrimp(XL) 1kg |
| G33 | Shrimp is good, the head is very large, and there is not much ice, after melting the head is almost the same, catching up with the activities to buy a lot, a total of seven bags, to make shrimp for the baby to eat, the babies love to eat. | Crystal Jade Raw Shrimp(XL) 1kg |
| G34 | The price, it's beautiful. The quality, too, is stellar. The ingredients are fresh, nutritious, delicious and healthy. Stir-fried vegetables stew soup hot pot, what are suitable, full of a big bag, can eat several meals. Jingdong cold chain is also powerful, sent to the ice, pleasant. | Crystal Jade Raw Shrimp(XL) 1kg |
| G35 | Goods praise big quality fresh full of flavor first-class emerald raw shrimp (plus size)  Emerald Jumbo Shrimp, inexpensive shrimp is big and nice, really good. Add to cartBuy Now | Crystal Jade Raw Shrimp(XL) 1kg |
| G36 | Very tasty shrimp, handled cleanly too, more even in size, good quality hmmm, ice is thin too hmmm, will be back again, very good stuff, worthy of kudos | Crystal Jade Raw Shrimp(XL) 1kg |
| G37 | Jingdong logistics is as fast as ever, received ice or bang bang, purchased many times, shrimp is as high quality as ever. It's easy to make a reduced-fat meal, thaw it in cold water for a few minutes, fry it in the pan, delicious Ψ(￣∀￣)Ψ | Crystal Jade Raw Shrimp(XL) 1kg |
| G38 | Meet the activities, the family will buy some more, good quality, Jingdong service is very good, the family children ravioli inside can also put more, daily cooking is also much more convenient! Home refrigerator inside a variety of frozen food, convenient and affordable! Convenient! Jingdong service is very good! | Crystal Jade Raw Shrimp(XL) 1kg |
| G39 | The National Union Emerald Jumbo Shrimp was very tasty, large and fresh. The date is into the period. Steamed egg cake with shrimp, fried shrimp with broccoli, and shrimp dumplings with chives and pork are very tasty. Purchased year round. Very good quality. | Crystal Jade Raw Shrimp(XL) 1kg |
| G40 | Guolian emerald shrimp is very delicious, large, fresh, date into the period, the quality is very good. Steamed egg cake shrimp, broccoli fried shrimp, leek meat shrimp dumplings, very tasty. Purchase year-round. | Crystal Jade Raw Shrimp(XL) 1kg |
| G41 | The size of the shrimp is more uniform, the base of the shrimp so big or so, it is very easy to do, thawed a little bit can be, change the method for the child to do, but also more like to eat, has been many times to repurchase the product. | Crystal Jade Raw Shrimp(XL) 1kg |
| G42 | This Guolian large shrimp meat bought many times, clean and tidy convenient to eat, fresh delivery to the doorstep is also frozen very well, Jingdong self-support convenient and fast delivery to the doorstep, the morning order, the afternoon received. | Crystal Jade Raw Shrimp(XL) 1kg |
| G43 | GUO LIAN Guolian Aquatic Black Tiger Shrimp without ice net weight 200g de-veined Large 13-23 Seafood Aquatic, shrimp full of meat fresh, do activities at a good price, worth buying. Express is also very powerful, quickly delivered. | Crystal Jade Raw Shrimp(XL) 1kg |
| G44 | This shrimp fried to eat or eat hot pot Temei, shrimp is very large, but also the United States has a lot of water, full pounds and full two, to the children to eat is very good, eliminating the need to buy live shrimp to do. Jingdong self-supporting cold chain delivery is superb. | Crystal Jade Raw Shrimp(XL) 1kg |
| G45 | Guolian brand black tiger shrimp shrimp, meat thick and tender, a large neat and tidy, fried shrimp flavor, excellent taste, the price meets a great deal, buy a very good value. | Crystal Jade Raw Shrimp(XL) 1kg |
| G46 | Shrimp size is uniformly full, very fresh, fresh flavor, clean and convenient food,, good quality, too much surprise, sex price and higher than high, really out of my expectation, raw delivery to the home, it is worth to rest assured to buy. | Crystal Jade Raw Shrimp(XL) 1kg |
| G47 | We have eaten two packages of shrimp, very fresh. The big ones are better for stir-frying, and the small ones are especially good for wrapping dumplings, we ate one packet each, the big ones fried cucumbers, and the small ones wrapped in veggie boxes and ate them, they were all quite fresh and tasty. | Crystal Jade Raw Shrimp(XL) 1kg |
| G48 | The family is now eating shrimp are purchased on Jingdong, has always chosen this black tiger shrimp, the head is very large and chewy very tasty, each time a packet of 1kg is just right for a family meal. | Crystal Jade Raw Shrimp(XL) 1kg |
| G49 | Bought many times the prawns, Guolian brand is trustworthy: always adhere to the very high quality of goods, every time I buy back has not called me disappointed, made the stir-fried shrimp sharp, elastic, worth repurchasing! | Crystal Jade Raw Shrimp(XL) 1kg |
| G50 | This is a small shrimp, suitable for making buns, shrimp scrambled eggs, porridge put some: delicious flavor, popping power! Just take them out a few minutes in advance, they defrost especially fast, contain less water, and are worth having! | Crystal Jade Raw Shrimp(XL) 1kg |
| G51 | Shrimp is what I always buy: good freshness, less ice, defrosted lumps, good texture and elasticity. It's a great accompaniment to sauteed shrimp and put some in congee. My family loves it and will always buy it! | Crystal Jade Raw Shrimp(XL) 1kg |
| G52 | This shrimp bought for the second time, it is really good, the head is quite big, the taste is very good, very clean, the shrimp threads are removed, it is very convenient to eat, the portion is very large, cost-effective, and the delivery speed is very fast. | Crystal Jade Raw Shrimp(XL) 1kg |
| G53 | Guolian big brand, already do not know how many times to repurchase, shrimp head is large, ice is very little, the taste is very good, the number of quite a lot can eat a few meals, while the price is right to Tuen a few more bags, I hope that the back will have activities. | Crystal Jade Raw Shrimp(XL) 1kg |
| G54 | Baby received a very fast courier, good service, domestic emerald raw shrimp shrimp full of large, shrimp line removed very clean very like, my children love to eat, big brand trustworthy, the date is very recent very fresh | Crystal Jade Raw Shrimp(XL) 1kg |
| G55 | The shrimp is still good! First of all, the price is very advantageous, and the shrimp is also very large, the texture is still relatively good, still very tasty! Logistics is also very fast, the courier boy is very strong, delivery to the home! | Crystal Jade Raw Shrimp(XL) 1kg |
| G56 | Bought several times, took advantage of the activities and bought a few bags, Jingdong activities are very strong ah, too affordable! Shrimp is relatively clean, the ice coating is not too thick, the size can also be, their own family daily stir-fry, do stuffing can be. | Crystal Jade Raw Shrimp(XL) 1kg |
| G57 | The National Union Aquatic Store has a wide variety of frozen meats that you usually buy in superior quality. I've bought this - dry grams of shrimp a number of times, medium size, not shrinking at all, also very clean, very tasty. | Crystal Jade Raw Shrimp(XL) 1kg |
| G58 | The ice coating of this shrimp is not too thick, thawing quickly, than their own hand-peeled shrimp tastes more Q-bouncy, shrimp line removal is also relatively clean, the shrimp back has a shallow cut mark, after thawing can be directly marinated into the flavor, easy to | Crystal Jade Raw Shrimp(XL) 1kg |
| G59 | It is said that the quality of Guolian emerald shrimp is very good, just in time for the event price, received a very large package. Shrimp weight loss and fat loss must, high protein and low fat, take a few in the morning and eggs with pancakes, especially fragrant! | Crystal Jade Raw Shrimp(XL) 1kg |
| G60 | Their shrimp is quite delicious, the family has children recommended to buy, full of a big bag, enough to eat for a long time, whether it is fried vegetables put inside or white water boiled to eat, the taste is very fresh. | Crystal Jade Raw Shrimp(XL) 1kg |
| G61 | Their shrimp is frozen, but the size is still big after melting, no shrinkage, we often fry cucumber shrimp for our children, and the flavor is also very fresh, always buy theirs, recommended to buy if you have children. | Crystal Jade Raw Shrimp(XL) 1kg |
| G62 | Real big shrimp, slowly a big bag, many times back to buy. Each shrimp has opened the back to take the shrimp line, it is very convenient to eat, the texture is also very strong, the child very much like to eat. Jingdong's cold chain transportation is too awesome, to the home is still frozen solid. | Crystal Jade Raw Shrimp(XL) 1kg |
| G63 | Guolian Jade Raw Shrimp was received long ago, shipped quickly and the courier guy was a force to be reckoned with. This product has been purchased many times, beautifully packaged, clean and tidy, sufficient portion, first-class flavor, excellent taste, rich in nutrients. Big brand is trustworthy, next time continue to patronize. | Crystal Jade Raw Shrimp(XL) 1kg |
| G64 | This shrimp is really good, good packaging, new date, fast logistics, small brother to the hand is still frozen, although the small size, feel quite big, taste Q pop, tender, slippery egg scrambled vegetables are very tasty, the activities of the price is not expensive, will buy back! | Crystal Jade Raw Shrimp(XL) 1kg |
| G65 | National Union Aquatic Products is very good quality, always buy this brand of shrimp, shrimp is fresh and clean, a little clean up and ready to cook, convenient. Buy frozen food on a hot summer day, well packaged. Good review. | Crystal Jade Raw Shrimp(XL) 1kg |
| G66 | Often in the Jingdong to buy fresh food, a variety of shrimp bought many times, Jingdong on the fresh food is very fresh, this time to buy the Guolian shrimp, medium size, received also frozen well, the child likes to eat cucumber fried shrimp, simple and easy to have nutritional | Crystal Jade Raw Shrimp(XL) 1kg |
| G67 | This shrimp from National Union is of good quality. Nicely packaged, generous portions, the shrimp are large, de-threaded, clean (no grit) and fresh. Processing is relatively simple and the texture is bouncy. Not bad! | Crystal Jade Raw Shrimp(XL) 1kg |
| G68 | The logistics was very fast, ordered on the same day and delivered on time the next day. The shrimp was a big packet, fresh and the kids loved it! Put on one side looks good and delicious. The quality is exceptionally good, very good value for money, will repurchase again da! | Crystal Jade Raw Shrimp(XL) 1kg |
| G69 | Shipped very quickly, Jingdong cold chain transportation is very good, to the home a little not painted, shrimp? Good, a single frozen, not too have ice ice, a big bag a lot, enough to eat a good while, the quality is also very good, the price is also good. | Crystal Jade Raw Shrimp(XL) 1kg |
| G70 | Great shrimp, great fit, great value for money. The flavor was so good that I bought it and used it to make shrimp dumplings. All gone in one second, a bit better than any other brand I've bought before, will continue after these are finished. | Crystal Jade Raw Shrimp(XL) 1kg |
| G71 | This shrimp is also a repeat purchase. The quality is over the top. Very good. It didn't shrink after freezing, it's about the same size and the meat is Q-tastic. Often make shrimp sliders for the kids and eat them underneath and they are great. Satisfaction. | Crystal Jade Raw Shrimp(XL) 1kg |
| G72 | I'm going to run out of this prawns if I don't rate it again, this prawns are very large and the threads are cleanly handled. Neat and good quality, not a lot of ice, it's all real, you can eat a bag for a long time | Crystal Jade Raw Shrimp(XL) 1kg |
| G73 | Multiple purchases Shrimp head is very large, steamed and stir-fried boiled are very tasty, the flavor is very fresh, shrimp meat is very tender, the price is also very beautiful, the arrival speed is fast, very satisfied, praise praise? | Crystal Jade Raw Shrimp(XL) 1kg |
| G74 | Very delicious? The price is very, very beautiful, much more delicious and affordable than the cafeteria 1 10 dollars for a few shrimp, arrived very quickly, overall a very satisfying shopping experience! | Crystal Jade Raw Shrimp(XL) 1kg |
| G75 | Multiple repurchase models, 6.18 activities cheaper than the super cheap a lot of shrimp, shrimp meat thick texture full, shrimp line has been removed, thawed without shrinking, practice versatile fried fried shabu shabu cold mix can be, the texture of crispy and tender elastic teeth shrimp flavor, packaging date new, businessmen shipments in a timely manner, the Jingdong distribution speed, goods received without thawing and water deformation, will be repurchased. | Crystal Jade Raw Shrimp(XL) 1kg |
| G76 | 已经吃的有一半了 满满的一袋 做汤做粥清炒都非常好吃 口感也特别好 Q弹Q弹的 主要是不用用手剥了 吃起来比较方便 还会在回购 | Crystal Jade Raw Shrimp(XL) 1kg |
| G77 | Recently in the weight loss, I heard that eating shrimp to reduce fast, this time to buy a lot. Cooked once especially delicious, shrimp Q bullet, Jingdong fresh logistics is also very fast, received frozen very good, eat at ease! | Crystal Jade Raw Shrimp(XL) 1kg |
| G78 | Especially especially satisfied, the quality is particularly good, no odor, the price is also affordable Shipping is very fast, things are well packaged, the price is good, I am very satisfied? , will buy back and will continue to buy back. | Crystal Jade Raw Shrimp(XL) 1kg |
| G79 | I bought it during the June 18th sale, and bought 4 packages at once, which was a really good deal! My family has always eaten this brand of shrimp, the head is very large, full of pounds and two, it is worth repurchasing a shrimp. | Crystal Jade Raw Shrimp(XL) 1kg |
| G80 | Multiple purchases of goods, really very good, especially when doing specials, simply too good value, this time is a super subsidy time to buy, affordable price, things are still the same as before, good value for money! | Crystal Jade Raw Shrimp(XL) 1kg |
| G81 | I've bought this brand of shrimp many times and the quality is pretty good. It's easy to stir fry a piece with assorted vegetables for a quick dish. It's also tasty to put some on fried rice. The shrimp freeze together and don't completely clump together and are easy to pick up! | Crystal Jade Raw Shrimp(XL) 1kg |
| G82 | Shrimp is very good, the size is also appropriate, Jingdong shopping assured, the cold chain is very good, a bag of shrimp a lot of ice bags, and the logistics is very fast. Affordable, very satisfied with a shopping. Eat very fragrant, very fresh, our family likes to eat. | Crystal Jade Raw Shrimp(XL) 1kg |
| G83 | Bought it many times, the shrimp is very big! Very good, 618 activities down very cheap, delivery is also fast, frozen goods packaging is also in place, good good. Waiting for double ten again to continue to buy, preferential buy and then look at the usual price do not want to buy. | Crystal Jade Raw Shrimp(XL) 1kg |
| G84 | Repeatedly repurchased numerous times. It says smallest size, but it's big enough. Go to the shrimp line, thin ice, about the same as no ice. Children especially love to eat, qq bouncy, the texture is particularly good. Take advantage of the big promotion to stock up on a few more bags. Punch it. | Crystal Jade Raw Shrimp(XL) 1kg |
| G85 | Guolian shrimp repeat purchase many times, mainly do shrimp scrambled eggs, shrimp fried rice, shrimp are very large, the outside of the ice coating is not thick, thawed water is not too much, dehydrated weight is also 1 kg, shrimp eat popping teeth and crisp, very tender. | Crystal Jade Raw Shrimp(XL) 1kg |
| G86 | Always buy this store's products, shrimp? The head is full and clean, thawed and slightly cleaned can be placed in the pot, burned out of the shrimp does not shrink, fresh shrimp flavor, very tasty, will continue to buy after eating! | Crystal Jade Raw Shrimp(XL) 1kg |
| G87 | This one Guolian jade raw shrimp is especially great, crystal clear, one grain is very big, there is no ice in it, and it is very convenient to eat as much as you can and pour out as much as you can. Shrimp scrambled eggs, shrimp fried mixed beans, super awesome, on the Jingdong shopping trustworthy. | Crystal Jade Raw Shrimp(XL) 1kg |
| G88 | This is really very good head is very large, it is well worth it in the event of a few packets at once can eat a month, will buy again in the future this brand of aquatic products are very clean and very hygienic and fresh | Crystal Jade Raw Shrimp(XL) 1kg |
| G89 | This shrimp is very convenient to buy and put in the refrigerator, sometimes when you want to eat, you can take out and fry a few, high protein, low fat, but also very healthy and delicious, basically do not need to put too much other seasonings ~ ~ | Crystal Jade Raw Shrimp(XL) 1kg |
| G90 | Shrimp is particularly great, before all the time to buy, 6.18 and bought two packages, very cost-effective, recommended that everyone buy, you can give the children stir-fried vegetables, wrapped dumplings eat very good? The best thing is that you have to be able to get the best out of them. The best thing about it is that it's a very good idea to buy it. Great? | Crystal Jade Raw Shrimp(XL) 1kg |
| G91 | My home refrigerator is full of Jingdong fresh, since the cold chain in the Jingdong buy fresh after a hair, get the hand are hard, the family's frozen food is now all in the Jingdong purchase, good value, fast delivery, service | Crystal Jade Raw Shrimp(XL) 1kg |
| G92 | The second purchase, the head is very large, no shrimp line 618 to engage in activities the price is very powerful, shrimp is very fresh, shrimp meat is elastic, cost-effective quite high, fried food to eat the baby is very favorite, will buy back! | Crystal Jade Raw Shrimp(XL) 1kg |
| G93 | Packaging is very good, delivery speed is very fast, and is the cold chain transportation morning order, received the goods in the afternoon delivery service attitude is very good, always put the error delivery to the door, on this point of things very satisfied! | Crystal Jade Raw Shrimp(XL) 1kg |
| G94 | Very good very good, especially good, 618 price is super cheap, a bag is only 60 dollars. Once a bag of brine, you can eat a few meals, very tasty, the taste is very good, will be back to buy back, I hope to do more activities. | Crystal Jade Raw Shrimp(XL) 1kg |
| G95 | Baby received, and the description of the same, like it is genuine, and the same as the entity store, the price is also favorable, decisive turn powder? Baby received, very surprised? , the design is reasonable, stylish and generous, continue to pay attention to, and later will buy back received immediately opened to look at it, very good, superb quality, value and high, recommend recommend recommend | Crystal Jade Raw Shrimp(XL) 1kg |
| G96 | The black tiger prawns were fresh and large and the portion was generous. Fried a cucumber is very delicious. The delivery is strong, the ice did not melt when it was delivered. The activity to buy very cost-effective. I hope that often have tempted me, hehehe. | Crystal Jade Raw Shrimp(XL) 1kg |
| G97 | More chewy, texture popping teeth, flavorful, slightly salty, meat is very firm, with some sweet, seafood in the delicious dishes, how to match the dishes are delicious. | Crystal Jade Raw Shrimp(XL) 1kg |
| G98 | Big head, stir-fry is more fragrant, reddish color, delicate meat, tender, delicious, rich in protein and vitamin A. It is very suitable for this season, which is good for replenishing calcium in the body. | Crystal Jade Raw Shrimp(XL) 1kg |
| G99 | This shrimp product is just great quality, fresh and flavorful. I have been buying and eating it and give a big shout out. Great product will keep on buying more and more. | Crystal Jade Raw Shrimp(XL) 1kg |
| G100 | I bought two bags of Emerald Raw Shrimp yesterday and felt that the quality was very good, so I ordered another bag of the smaller size, which is easy to eat and affordable. The quality and freshness of National Union Seafoods is guaranteed, and I will buy again in the future. | Crystal Jade Raw Shrimp(XL) 1kg |
| G101 | The order placed yesterday evening arrived this morning, the Jingdong express is really trustworthy. Open the foam box inside the ice is still hard shrimp, intact, the head is very large, the price is very affordable. | Crystal Jade Raw Shrimp(XL) 1kg |
| G102 | 618 activities have promotions, but also first look at the evaluation, the size is really not small, a big bag full of two pounds, fried vegetables or mixed salad are very good, Jingdong quality assurance, summer more white, winter melon fried shrimp | Crystal Jade Raw Shrimp(XL) 1kg |
| G103 | Shrimp arrived, very satisfied, steamed fried shabu shabu stew are good. Not in vain I risked being sprayed by the other four teammates of the king, to evaluate the baby, no more, I have to go, my teammates are still waiting for me to order again. | Crystal Jade Raw Shrimp(XL) 1kg |
| G104 | I've repurchased it many times and stocked up on a big bag in time for the 618 event. It's very convenient and the shrimp are clean. The threads on the back of the shrimp have been picked clean. Just melt it before eating, the meat is very firm. | Crystal Jade Raw Shrimp(XL) 1kg |
| G105 | Guolian is a famous aquatic brand in China, the quality is trustworthy, our family has always chosen Guolian aquatic products, including this enlarged black tiger shrimp, the head is really big, the taste is very good, the children also love to eat, we recommend everyone to buy. | Crystal Jade Raw Shrimp(XL) 1kg |
| G106 | Shrimp is very good, quite large, cooking is very convenient, can not always go to the market to buy, shrimp line is also quite clean, no lumps, only only clear, too convenient, while the activities of the purchase of a more cost-effective | Crystal Jade Raw Shrimp(XL) 1kg |
| G107 | Super large, very good, in addition to a little expensive, the bag has a seal, very convenient, bought a lot of things on Jingdong, I hope to do more activities, the price is more favorable! Express delivery is also very good | Crystal Jade Raw Shrimp(XL) 1kg |
| G108 | Yesterday's order to buy the National Union aquatic shrimp, a large package of a small package, received in kind, feel the small shrimp cost-effective some more, the National Union aquatic brand over the top, the quality is trustworthy, praise, praise! | Crystal Jade Raw Shrimp(XL) 1kg |
| G109 | This Emerald Raw Shrimp still tastes like it's coming together, crispy and full of flavor. It's easy to cook a handful at a time. buy it at 6.18 with coupons and red packets for a great price, and if you have a lot of red packets, you can get it for nothing, which is a super deal. | Crystal Jade Raw Shrimp(XL) 1kg |
| G110 | 618 have activities when the order, cheaper than usual a lot, for office workers, cooking is very convenient, cucumber fried shrimp, easy to save. There are also his shrimp dumplings are also very good, together with the order can also save shipping costs. | Crystal Jade Raw Shrimp(XL) 1kg |

| serial number | Word-of-mouth text ([Basa catfish](https://baike.baidu.com/item/%E5%B7%B4%E6%B2%99%E9%B1%BC/5731436) H) | offerings |
| --- | --- | --- |
| H1 | Split into two separate packages, a package of 600 grams, eat a meal just right. Jingdong fresh cold chain transportation is very reassuring, received or hard. Cost-effective during the promotion, all five stars! | Basa Fish Fillet 600g |
| H2 | My family has purchased Guolian Balsa Fish for many times, and I have stocked up in the refrigerator. The fish is not only of good quality, good taste, new date, but also scale enough, each bag of two fish net weight> 700 g. In short, this e-commerce business integrity business, and at the same time is the Jingdong logistics delivery, which is enough to make my family locked Guolian continue to repurchase Oh! | Basa Fish Fillet 600g |
| H3 | Balsa fish is very good, clean, easy to take care of, want to eat in advance of frozen, cleaned, fried, braised, steamed, how you want to eat can be, the flavor is delicious, fresh, has been many times back to buy. | Basa Fish Fillet 600g |
| H4 | Has received the balsa fish, packaging is very good, a bag with 2 balsa fish, is frozen, foam box also has dry ice, balsa fish is gutted, want to eat the fish as long as a little bit of washing, can be processed to do dishes to eat, very convenient, my family has been purchased many times, this time it is to buy back. | Basa Fish Fillet 600g |
| H5 | Very good balsa fish product product is a big brand The quality and quality of the product is very guaranteed, the product is well packaged, good quality, frozen packaging is more able to increase the shelf life. | Basa Fish Fillet 600g |
| H6 | Have purchased this frozen balsa fish many times. Packaging is very good, when the foam box is delivered, the fish inside is still frozen, not melted at all, this is three to go balsa fish, cleaned up very well, when you want to eat the fish, just clean and clean and then do the braised fish can be, very convenient, save time. Express delivery is quite fast. | Basa Fish Fillet 600g |
| H7 | This fish is a regular food in my freezer, fresh, good taste, thawed and simply cleaned, eliminating the need to scale and pull out the gills, as the cleanup is already very clean! | Basa Fish Fillet 600g |
| H8 | Very satisfied with a shopping, very tasty, good taste, the most satisfied with the praise of a shopping, recommended to eat fish friends to buy, and will return to buy, very tasty, well packaged, the courier sent very quickly. | Basa Fish Fillet 600g |
| H9 | Reliable, product quality is great logistics is very fast and powerful, a one-time buy a lot of product quality is also good, great and reliable, packaging is also good, the price is good value for money! | Basa Fish Fillet 600g |
| H10 | This balsa was exceptionally fresh and then the portion was generous. The packaging is very good. What about the balsa fish, it was very fresh. The balsa is wider and the meat is tender. Very delicious. This time there is a promotion, the price is more favorable, fast delivery. | Basa Fish Fillet 600g |
| H11 | Balsa fish fillet is often purchased, the elderly and children like to eat, the fish meat is delicate, pure flavor. Use it to make steamed fish, fried fish is a good choice, the child lunch box often with some, rich in nutritional value | Basa Fish Fillet 600g |
| H12 | The factory date is February 6, 2023 and the factory shelf life is 18 months. The date is not bad. The quality of Guolian Balsa Fish Fillet has been very stable. The elderly also particularly like it, basically there are no thorns, and it is very good for both pan frying and making pickled fish. The quality is definitely solid. Jingdong logistics is great. The order was placed at 12 o'clock at night, and basically arrived in the afternoon. Jingdong activities of the preferential strength reduction, straight resistance ah, the refrigerator is full, but also can not resist the purchase. All five-star praise. | Basa Fish Fillet 600g |
| H13 | First time buying this brand of balsa fish. Hope it is very tasty. The price is still good, the packaging is good, and the balsa fish looks neat and wide. Express delivery is very strong, cold chain delivery, worth buying. | Basa Fish Fillet 600g |
| H14 | Authentic East China Sea balsa fish, well, the head is very large, after unwrapping is two small packets of individually wrapped, full of pounds and two, very fresh, the taste is very good. The old people and children in the family all like it very much. | Basa Fish Fillet 600g |
| H15 | Baby received, the quality is very good, exquisite packaging, excellent material, better than expected, gift for self-use is very suitable, next time continue to buy! Good shopping experience! Thank you very much for the Jingdong self-support! | Basa Fish Fillet 600g |
| H16 | It is the ideal product? , the quality is good, the logistics is also very fast, worthy of praise! Big brands are trustworthy, good things everyone loves, thanks to the very good shopping experience brought about by Jingdong Self-support! | Basa Fish Fillet 600g |
| H17 | I've been using a variety of ingredients from Guolian Aquatics in my house, and they're quite good. This East China Sea balsa fish has firm, garlic clove meat and is very fresh. If you use a pressure cooker even the bones can be pressed su, it is worth buying! | Basa Fish Fillet 600g |
| H18 | This balsa fish has been repurchased many times, it should be authentic wild balsa fish, the texture is very, southwestern, and then the flavor is very good, it has been repurchased many times, next time continue to buy. | Basa Fish Fillet 600g |
| H19 | Bought many times this balsa fish, fresh and delicious, and three to go very clean, trust the Jingdong self-supporting products, attentive service, quality assurance, buy cold chain on the Jingdong self-support, well-packed, good quality! | Basa Fish Fillet 600g |
| H20 | GUO LIAN Guolian East China Sea boutique balsa fish is very good, remove the head and tail, uniform size, good value for money, a word good, meat thick and tender, packaging is very delicate, to the home or frozen hard, logistics is very fast! | Basa Fish Fillet 600g |
| H21 | Self-flagship store official website authentic, this balsa fish bought several times, eating is very convenient, wash it can be cooked and eaten, relatively clean, balsa fish flavor is very strong | Basa Fish Fillet 600g |
| H22 | Bought a few times this balsa fish, really thick meat, a lot of meat. Quite good, like to eat fish, every time you do the braised, sweet and sour Feel good about it, the next time you make a finished fish, to follow up on it, now Jingdong old fish, think the quality is good it, received the goods are very fresh, the packaging is also tight Worthy of buying | Basa Fish Fillet 600g |
| H23 | This pomfret is quite good. And the price of Jingdong 618 is also very suitable, to be much cheaper than the supermarket to buy their own, and delivered to the home packaging is still very good, not frozen at all. Very worth buying. | Basa Fish Fillet 600g |
| H24 | This is the second time I've purchased this balsa, it's basically a medium and the quality is quite good. It's especially good for dry frying or braising. Jingdong's packaging is also very good, arrived home completely unfrozen, the quality of the barramundi praise! | Basa Fish Fillet 600g |
| H25 | Jingdong's fresh food is great, yellowtail is much cleaner than we buy in the market Also cost-effective Catch the event price is very cheap If not for the refrigerator can not put high and low and then a few boxes Commend the logistics, which is a lot of dry ice a lot of frozen after receiving a very solid | Basa Fish Fillet 600g |
| H26 | Things are good, there are discounts, you can rest assured to save money to buy, do not hesitate Baby value for money, very much like, the next time will come back to the baby is really great, I like it very much, my friends also say good to come to, recommended to buy! | Basa Fish Fillet 600g |
| H27 | This time 6.18 to take advantage of the event to buy, meat quality is very good, fried balsa fish is very fresh. The price is also very affordable, cheaper than buying in the supermarket, the balsa fish is also very fat, very good, recommendable! | Basa Fish Fillet 600g |
| H28 | Taking advantage of the Jingdong activities to buy, the price is too too good value, bought three bags, has eaten a bag, the family elderly teeth can not, so I bought this kind of fish fillet without thorns, very good! And not much ice, the meat is quite thick. | Basa Fish Fillet 600g |
| H29 | Very good and good value. Often buy household goods on Jingdong Grocery. The price is cheaper than the supermarket mall when it comes to activities. Quality is guaranteed and can be delivered to the door of the neighborhood. Jingdong express speed is also fast enough Express brother attitude is very good. Come come come continue to buy buy buy up. | Basa Fish Fillet 600g |
| H30 | I feel that this balsa fish is a better buy, the quality is good and the price point is reasonable. The key is that the fish is fresher and the balsa fish has fewer spines, making it safer to eat, especially for those in a hurry to eat! | Basa Fish Fillet 600g |
| H31 | Not a small balsa fish, a meal just one, specially bought three to go balsa fish, cooking up a lot more convenient, Jingdong delivery is very fast, the family fresh food are all bought by Jingdong, and so on after eating will also buy back, thank you Jingdong. | Basa Fish Fillet 600g |
| H32 | This balsa fish taste very good, there is a strong flavor, has been purchased a number of times, each time very satisfied, the whole family especially like to eat, especially the children, logistics courier service is very fast! | Basa Fish Fillet 600g |
| H33 | This swordfish is good, very thin very fresh, there are two small packages, each inside is 600 grams, eat up more convenient steamed ah, stewed are quite good, Jingdong activities when buying some inventory | Basa Fish Fillet 600g |
| H34 | Once the goods received, the delivery degree is very fast, Jingdong delivery, service attitude is very good, the price is cheap, affordable, arrived and did not melt, the quality is very good, the fish is very fresh, the price is also very cheap, we recommend that everyone buy! | Basa Fish Fillet 600g |
| H35 | Guolian three go balsa fish, domestic East China Sea yellowtail, very good. Fish for lunch, the fish is fresh, fatty and flavorful. Three to go balsa fish, very convenient, from the refrigerator out of the slippery finish can be used, save time. Sufficient portion, honest business, will go back to buy. | Basa Fish Fillet 600g |
| H36 | Guolian aquatic products are also bought many times, this three to go yellowtail especially like, a pound, the right size, steamed braised are delicious, tender meat, fresh, and a little cleaning can be used, convenient and quick. | Basa Fish Fillet 600g |
| H37 | Bass carp are very large, burned once feel the quality is particularly good, not fishy, the meat is also very firm, Guolian home seafood and aquatic products are particularly good, sea bass is also often buy, not stepped on mine, will support more | Basa Fish Fillet 600g |
| H38 | Goods have been received, the packaging is very tight, foam box, inside the freezer accounted for half of the big summer, outdoor temperature is so high, the fish frozen hard, looks very appetizing. Merchants are attentive! | Basa Fish Fillet 600g |
| H39 | Today received the courier, opened to see, feel truly super like, super cost-effective ~ cost-effective is also very high! Friends said I bought a super value, all want to buy this it, has been recommended to good friends, haha, next time will come to buy .... Friends who are still hesitating to get down quickly, conscientious recommendation oh! This time to do activities is also very good ~ ~ cost-effective is very good. Logistics, the seller's shipping speed is also very fast, the service is also very in place, fast ~ good service! Previously also bought this, but genuinely not as good as this time to buy, this time to buy genuinely earned, haha! | Basa Fish Fillet 600g |
| H40 | Great brand from National Union, have been buying for years. The fish is very fresh, the cold chain is also strong, the arrival is still frozen bonbon hard. The three go balsa fish, but also still have to scrape the fish scales, the whole family's favorite steamed yellowtail, cooking is not easy to turn over, do it simple, have a hand on it, the finished product is simply delicious. | Basa Fish Fillet 600g |
| H41 | This is a great quality balsa fish for braising, and steaming as well! The meat is very firm and has a nice texture! Catch the event price is still very affordable! Jingdong self-support, cold chain is guaranteed, rest assured to buy! | Basa Fish Fillet 600g |
| H42 | The big yellowtail is good, three to go, but also relatively clean, simple processing can be done to eat, meat is also good, the head is not small, a catty or so, quite good. Jingdong self-supporting logistics is very fast, packaging is tight and intact. | Basa Fish Fillet 600g |
| H43 | Jingdong Self-supporting store goods are genuine and trustworthy to buy. Guolian Aquatic Jingdong Self-supporting Store Balsa fish, meat is delicate and fresh, easy to eat, no head and no tail. Really good! | Basa Fish Fillet 600g |
| H44 | Three to go balsa fish received, the quality is very good, delivered to the home is still hard stick, did not melt at all, very fresh, logistics is also very fast, the future will return to buy, give five-star praise. | Basa Fish Fillet 600g |
| H45 | Packaging is very good, the value is good, the quality is not a problem to engage in activities to buy, the appearance and description of the same, beautiful and practical, the material feel good, this color I like, cost-effective, very nice | Basa Fish Fillet 600g |
| H46 | The quality is very good, completely exceeded expectations, the delivery speed is very fast, the packaging is very careful, strict, very satisfied with a shopping, this is a repurchase, three to go balsa fish is really convenient. | Basa Fish Fillet 600g |
| H47 | Very large. Also very convenient. All washed up. Opened and ready to go. Additional comments. Got it and made the date is also very fresh, for fast food also said very convenient. Recommended for everyone. | Basa Fish Fillet 600g |
| H48 | Balsa fish and shrimp from Guolian has always been my repurchase of food, must stock up during the event, three to go balsa fish handled cleanly, frozen state is very good to ensure that the food is fresh and nutritious, family members are very much like this one, the big brand quality is guaranteed! | Basa Fish Fillet 600g |
| H49 | Guolian Fine East China Sea Basa Fish, have bought it many times, the product is well packaged and the Basa Fish is fresh and tender. Very delicious. This time there are promotional activities, the price is more favorable, fast delivery. | Basa Fish Fillet 600g |
| H50 | This balsa is especially fresh. The rainbow curtain kid on top. There was quite a lot of it and then the portion size was adequate. Packaging is very good. What about the balsa fish, very fresh. The balsa fish child is wider. Then. The logistics of Jingdong is also very fast. And the deliveryman this. Also very good! Jingdong sales of this product's are trustworthy. | Basa Fish Fillet 600g |
| H51 | This is one of the best balsa fish I've had lately, I usually don't have time to make it, make it once and eat it, it's genuinely delicious, love it, next time I'll buy some more and put it in the fridge and wait for the weekend to make it, it's very good! | Basa Fish Fillet 600g |
| H52 | This is a very good balsa fish product. The product is a big brand, the quality and quality of the product of a big brand is very guaranteed. The packaging of the product is good, the product is packed in frozen packaging, the quality and quality of the product in frozen packaging is more guaranteed, the shelf life is longer, the product is a very good balsa fish product of a big brand suitable for daily consumption and suitable for daily storage. | Basa Fish Fillet 600g |
| H53 | Bought several fish at once, has been repeatedly re-purchased many times The elderly at home like to eat It tastes good It's fresh and tasty It's good It will continue to buy It's very good It's also very fast logistics | Basa Fish Fillet 600g |
| H54 | The three going balsa fish netted 1 pound a piece, quite a big one. It was cleanly handled as well. Very fresh! Easy to take out and thaw and wash for the pan. Bought a lot of fish at once during the event, super good deal! | Basa Fish Fillet 600g |
| H55 | This brand of flatfish bought a few times, very fresh braised flavor is very good taste like, in the Jingdong buy affordable quality assurance, packaging tight express delivery to the home is also frozen very good also want to come back to buy. | Basa Fish Fillet 600g |
| H56 | This balsa fish is the family perennial in the repurchase of products, very good, every time did not let me disappointed, delicious, will continue to repurchase, there are activities when the price is more affordable, you can rest assured that the purchase! | Basa Fish Fillet 600g |
| H57 | The yellowtail is good, better than what I bought in the market before, and it has been processed cleanly, a few more times to wash it will be able to steam on the pot. Logistics is very fast, and packed with ice packs, the ice packs did not melt when it arrived, strong. | Basa Fish Fillet 600g |
| H58 | I really like this brand's products, good quality at a good price, and it's a great deal to buy when you come across a promotion. This product has a fresh date of production, easy to make, tasty, recommendable good product. | Basa Fish Fillet 600g |
| H59 | Time passes quickly, the old family has a custom, every time to the Dragon Boat Festival will be sent to the elders of the family blessings and small gifts, this time to buy dumplings brother yellowtail, the courier is still relatively fast, eat and then make up the comment! | Basa Fish Fillet 600g |
| H60 | Although the price seems to be higher than the supermarket, but the ice content is much less, no fishbone trouble, easy to use, good price-performance ratio, will continue to repurchase, if you can often do activities would be very good! | Basa Fish Fillet 600g |
| H61 | It is a repurchase buy for a long time, eat and then come back to comment. Balsa fish is very fresh, are in the middle, very good, fish fry, and then add the carrot boil flavor, the taste is beautiful, occasionally change the taste, spice spice, or very good, Jingdong fresh really help me solve the problem of eating, because it is a lazy person na, oh, sincerely thank Jingdong! Will, as always, support drops! | Basa Fish Fillet 600g |
| H62 | ? I received the baby, the color is very nice, the texture is also very good, the look is simple!!!? It is what I want! Will come back next time!!!? Never thought such an affordable price,? can buy such a good baby,? Cost-effective is very high, I like it very much oh? Do not hesitate to buy wow! No cheap feeling, very high-end, especially great! Shipping speed is fast, the seller's service is also good, so far very satisfied! | Basa Fish Fillet 600g |
| H63 | Bought a few times this small balsa fish, activities cheaper than the seafood store, thawed and cleaned very well, fried to eat meat is very tender, the family likes to eat, I hope that more promotions, will often pay attention to. | Basa Fish Fillet 600g |
| H64 | Although the price seems to be higher than the supermarket, but contains much less ice, no fishbone trouble, easy to use, cost-effective, 618 activities, equivalent to the right discount, a one-time purchase of a lot of Tuen Mun | Basa Fish Fillet 600g |
| H65 | Hot pot everyone loves to eat, we like the whole pickled fish pot base, and then some balsa fish fillets eat too delicious, before the supermarket will choose the hot pot ingredients, occasionally Jingdong have activities on the Internet to buy, Jingdong Express is very powerful, the first night to order, the next morning, the weekend continue to stock up, the next time you eat will not have to run to the supermarket, convenient and fast, point of praise! | Basa Fish Fillet 600g |
| H66 | Always like to buy a variety of goods in the Jingdong self-support, many brands, quality is guaranteed! | Basa Fish Fillet 600g |
| H67 | This balsa fish is an activity to buy, often buy the products of the State Union , the price is quite suitable, logistics is very fast attitude is also very good, the fish to see is also very fresh, the packaging is also very good, Jingdong loyal fans, just this year, the activities of less. | Basa Fish Fillet 600g |
| H68 | Jingdong 618 is here, do not buy something, are sorry for such a favorable price. The Spring Festival to now, the refrigerator tuned things are almost eaten, all kinds of daily necessities to big purchase. This balsa fish, flavorful, suitable for deep-frying, the family loves to eat, has been a number of repurchase. | Basa Fish Fillet 600g |
| H69 | It is a very affordable product for the price, there are two large pieces inside a bag, which is not so convenient if you have a small family as it will seem too much. The serving method is very simple, it is frozen, cut into strips, and then make pickled fish with pickled vegetables and vermicelli! | Basa Fish Fillet 600g |
| H70 | Balsa fish fillet, steamed braised with air fryer baked, how to do are delicious and nutritious high-protein food, recommended, but also recommended for regular consumption to ensure that the body is strong and invulnerable to all diseases, the fish should be eaten more Oh. | Basa Fish Fillet 600g |
| H71 | Balsa fish is very fresh, quite delicious, wearing an ice jacket, thawed fish scales do not fall off, is a little small, suitable for frying, braised feel small, packaging, logistics, delivery to the hand of the whole frozen, all five stars it. | Basa Fish Fillet 600g |
| H72 | Bought a number of times the product, it can be, the flavor is okay, more palatable it, the price is also okay, especially when there are activities is still good, Jingdong logistics is also fast, the next day arrived! | Basa Fish Fillet 600g |
| H73 | This time I bought two kinds of fish yellowtail and golden pomfret yellowtail has been eating yellowtail Guolian aquatic brand yellowtail bought a few times still more satisfied with the fish is fresh, fast delivery after-sales service is also good activities to buy the price is very good | Basa Fish Fillet 600g |
| H74 | Like to eat fish, especially balsa fish, freshwater fish technology and ruthless live too much, or more saltwater fish is relatively safe, this balsa fish is domestic, delicious, just do a little trouble, first scrape the scales, and then clean up the viscera, and then add the flour to fry a little, and finally on the braised. | Basa Fish Fillet 600g |
| H75 | This Jingdong Mall to buy this balsa fish is a good big big one, full of 500 grams, it looks relatively fresh, although it is farmed fish, the price is a little more affordable, sautéed and steamed are good Oh! | Basa Fish Fillet 600g |
| H76 | Express very timely, very satisfied, basically are still frozen it, did not melt, but the bag written on the dragon bar, is a sub-brand, I do not know how, I hope it is delicious, look at the handling of the pour is still relatively clean | Basa Fish Fillet 600g |
| H77 | GUO LIAN Guolian East China Sea Fine Balsa Fish 1.2kg Head and Tail Removed Domestic Deep Sea Fish: speed of delivery, cold chain shipping, fresh products; 2 large packs of individually wrapped inside, easy to eat. 618 promotional purchase at a discounted price. | Basa Fish Fillet 600g |
| H78 | Kok Luen Balsa Fillet, boneless and spineless, with very little ice (water) and very fresh, this is probably the best of the many kinds of commercially available balsa fish, unfortunately, the price is a little higher and can only be purchased when there is a special offer. | Basa Fish Fillet 600g |
| H79 | Balsa fish is a regular food fish at home, three go into the nest is very convenient, cooked out of the flavor and no fishy taste. The price of this purchase is not bad, I feel that the fish is still a little thin, may be due to the season. | Basa Fish Fillet 600g |
| H80 | It's been a while since I've done this before I thought to comment, it's been a while since I've had such a good balsa, it tastes good and I hope the good price comes back again before I stock up on a few more. The quality is also really good, feel free to buy can | Basa Fish Fillet 600g |
| H81 | Guolianli this swordfish quality is good, it seems to be divided into two small packages, each package has 600 grams to eat more convenient, balsa fish is also quite wide, the activities of the time the price is also more beautiful, buy a little more | Basa Fish Fillet 600g |
| H82 | The balsa fish was nice and clean, no head or tail. It does follow what the description says above, head and tail removed. I haven't eaten this purchase yet, but it mainly just looks good. Should come back to buy this one in the future | Basa Fish Fillet 600g |
| H83 | Tough luck with the courier guy, delivering first thing in the morning.  Took advantage of the last 6.18 frenzy to stock up on another order of yellowtail. Yellowtail is how to eat will be delicious fish. The meat is delicious, no fine spines, steamed, braised, fried and grilled are delicious, lazy to cook a bowl of yellowtail noodles, soup fresh flavor and nutrition. | Basa Fish Fillet 600g |
| H84 | This is the fifth time I think I've had it, and lately the texture of this fish is not as firm as it was at the beginning. I hope it's just me and that my aunt's cooking salty issues are clouding my judgment of this fish, the price has still always been very much to my liking. | Basa Fish Fillet 600g |
| H85 | This 618 event bought, the portion is full, full of discounts. I hope merchants have more of this kind of event, the merchants can quickly recoup their money and the customers can get a good deal. This fish is big and substantial, saves the trouble of breaking fish | Basa Fish Fillet 600g |
| H86 | This is one of the balsa fish that we have repurchased several times, they are all mid-range, easier to pack and save time. Basa is a deep sea product and sometimes you can catch it fresh. But none of the frying and braising is too demanding either. All in all, good reviews! | Basa Fish Fillet 600g |
| H87 | Jingdong Fresh this speed, ordered that night, the next morning was delivered, the date of production is February, the shelf life of 18 months, balsa fish quality is still good, happen to be the activities of the stocking point. | Basa Fish Fillet 600g |
| H88 | This product says it's triple removed, but you actually have to remove the guts of the balsa fish yourself. The width of the balsa fish was ok and the meat was ok. I hope there will be more events in the future. Benefit to the general consumers. Believe in the quality of the products of Guolian Aquatic. | Basa Fish Fillet 600g |
| H89 | 618 Jingdong Mall activities, this yellowtail is particularly cost-effective, cheaper than the original purchase of N times, if not the refrigerator can not put, that must also buy more. Yellowtail is three to go, a little sober can be convenient. | Basa Fish Fillet 600g |
| H90 | Balsa fish is really big, the whole 2, the price is not expensive, 618 to buy things is cheap, buy a big pile of fresh food, the big refrigerator at home can not put, can eat for a long time it, happy. | Basa Fish Fillet 600g |
| H91 | Yay for writing every time. This time I bought a few bags and the freshness is average average. National Union Aquatic three go very clean, packing is very save this point is very good. In addition or price has a large change, the purchase can choose. | Basa Fish Fillet 600g |
| H92 | I've bought this yellowtail before. Taking advantage of the current 618 to do activities, the price is favorable, and the brand is superior, plus the platform of Jingdong, and then get down to buy it again. Three to go yellowtail, easy and simple to cook, recommendable. | Basa Fish Fillet 600g |
| H93 | Great praise, given to the Jingdong Direct and this item, there is a picture of the truth, I'm a multi-year plus member, a lot of discounts, quite suitable, like friends, you can order, fast delivery, quality is guaranteed! | Basa Fish Fillet 600g |
| H94 | Balsa fish is large, viscera fish scales have been removed to eat very convenient, but in fact there are still some corners of the fish scales are not scraped clean, need to deal with it again, otherwise eating affects the texture, I usually used to fry and steamed, convenient and delicious. | Basa Fish Fillet 600g |
| H95 | Catch up with the activities of the Jingdong to buy a few balsa fish, this three to go is still relatively trouble-free, chemically open a simple treatment can be, every time there is an event will purchase a few balsa fish, affordable price is not bad! | Basa Fish Fillet 600g |
| H96 | I have bought a lot of balsa fish in Jingdong, compared to the best quality of balsa fish in Guolian, and then buy balsa fish only choose Guolian, uniform size, new date, good packaging, fast delivery, basically the same day delivery! | Basa Fish Fillet 600g |
| H97 | Perennial purchase of the video is particularly suitable for the elderly children to eat did not eat, do it is also relatively simple, you can do tomato fish can also be steamed, you can have you look for a can taste okay this quality than the other to buy the price is slightly more expensive is also appropriate is also very suitable, recommended to all of us | Basa Fish Fillet 600g |
| H98 | Bass head and balsa fish body are bought, taste the different body parts, which meat is more like, in general, balsa fish thawed and like to eat steamed or air fryer fried to eat, are more good! | Basa Fish Fillet 600g |
| H99 | This balsa fish is very good, it is already three to go, processed, very clean. Cleaned can eat the price is very cheap than there is that test certificate, so it is very reassuring to eat the things of the Jingdong, are big brands, so very assured, direct purchase can be, it is indeed very good, this brand is not added any additives, this balsa fish is the original strip of three has been processed and cleaned the balsa fish is very good, the price is very affordable, and has been repurchasing. | Basa Fish Fillet 600g |
| H100 | Multiple purchases, this 618 activities and purchase, the elderly and children at home are more like, steam and stew can be washed a few times can be used directly, the viscera processing is very clean, Jingdong to buy something more convenient and assured | Basa Fish Fillet 600g |
| H101 | This balsa fish is very good is the whole has three go is already processed clean wash can eat the price is also very cheap uh now do not have that fish fillet only this balsa fish he this best is not add any other things, did not add the potion whatsoever, just the fish ingredients, only fish nothing, so it is indeed very good, I just this do not buy that kind of soak through the potion. | Basa Fish Fillet 600g |
| H102 | My daughter's favorite, makes superb soup, no spines, easy to take with meals. It is two strips in a bag, thawing to two strips? together, this is not good ???? If it's individually wrapped, it's perfect! | Basa Fish Fillet 600g |
| H103 | I have bought many kinds of balsa fish, finally got a cozy one, overall very good, good price, fast delivery, all the middle section, some a little soft but does not affect the whole,,, convenient and quick, thanks to the seller, should come again in the future ah | Basa Fish Fillet 600g |
| H104 | Delivered to my door, the quality is very good, really out of my expectation, the packaging is very careful, thank you very much, good luck with business! It's very convenient to have things kept fresh with ice packs and frozen hard. Bought before or good. | Basa Fish Fillet 600g |
| H105 | Multiple repurchase, a regular ingredient in your home refrigerator. It's great for making pickled fish, no fish spines, and it tastes great. It can also be diced or sliced as needed, and it is very convenient to add some side dishes and fry it. Logistics and packaging are satisfied! | Basa Fish Fillet 600g |
| H106 | The first time I bought small balsa fish to eat, after thawing the internal organs those are very good to clean, made fried small balsa fish to eat, it was very successful, the skin of the fish is crispy, except for the fish head and the middle of the fish bone, the rest of the fish can be eaten, the meat is very tender, not fishy. | Basa Fish Fillet 600g |
| H107 | I've bought balsa fish many, many times, it's really good, the recommended way to eat it is steamed, and the freshness of the fish needs no introduction ~ this time I bought it for a friend, who was especially happy, and I hope to do more promotions haha! | Basa Fish Fillet 600g |
| H108 | The factory date is February 5, 2023, and the factory shelf life is 18 months, which is still plenty of time. Like in the case of Jingdong Fresh. It's best to buy from local warehouses. Like this Guolian aquatic balsa fish quality is particularly good, basically nothing ice, balsa fish has no thorns, the elderly especially like to eat, nutrition and particularly rich, and will not grow fat. Cooking is also particularly convenient, very good. Should seem to be this morning's order. It arrived in the afternoon, and this speed local warehouse is really convenient. Unlike a senior citizen machine I purchased for my child, actually shipped from the Chengdu warehouse, yesterday's order, actually today is not sent over, so troublesome. You have to especially thank the Jingdong logistics Xiaowei, the service attitude is particularly good. All five-star praise. | Basa Fish Fillet 600g |
| H109 | Guolian this fish is great, have bought it many times. Very convenient to arrive at home and wash can be done to eat, braised steamed are very tasty. Jingdong logistics is also very fast. Service is also very good, the next time there are activities will buy. | Basa Fish Fillet 600g |
| H110 | Activity price stock up, especially cost-effective ~ people in middle age eat less red meat, eat more fish, good for the cardiovascular and cerebral blood vessels ~ the date is quite new, but also in accordance with the agreed time of delivery, very satisfied ~ fresh food in the Jingdong purchase is very assured ~ | Basa Fish Fillet 600g |
